# Exploring viscosity sensitivity of π-extended coumarin fluorogen-based PRPGs for precise photorelease of valproic acid: detection and defibrillation of TDP-43 aggregation

**DOI:** 10.1039/d6sc03258c

**Published:** 2026-08-12

**Authors:** Subham Pal, Sagarika Das, Suchhanda Biswas, Sri Dinesh Murugan, Nihar Ranjan Jana, Asoke Prasun Chattopadhyay, N. D. Pradeep Singh

**Affiliations:** a Department of Chemistry, Indian Institute of Technology Kharagpur Kharagpur 721302 India ndpradeep@chem.iitkgp.ac.in; b Department of Bioscience and Biotechnology, Indian Institute of Technology Kharagpur Kharagpur 721302 India; c Department of Chemistry, SRM University Chennai 603203 India; d Department of Chemistry, University of Kalyani Kalyani 741235 India

## Abstract

Alteration of cellular microenvironment viscosity by protein aggregation plays a crucial role as a biophysical parameter that reflects abnormal cellular behaviour, leading to neurodegenerative diseases such as Parkinson's disease, Alzheimer's disease, amyotrophic lateral sclerosis (ALS), frontotemporal lobar degeneration (FTLD), *etc.* Herein, we report the design and development of a series of coumarin fluorogen-based photoremovable protecting groups (PRPGs, 5a–d) with variations in substitutions tethered with a π-extended linker that integrate viscosity sensing with visible-light-triggered release of bioactive molecules. By introducing π-extended conjugation and systematic substitution, the coumarin fluorogen-based PRPGs exhibit twisted intramolecular charge transfer (TICT)-based fluorescence modulation in response to microenvironmental viscosity. Comprehensive photophysical and photochemical investigations, supported by theoretical calculations, identified PRPG 5d as the most sensitive viscosity-responsive system with green-light absorption. Under viscous conditions, restricted bond rotation suppresses nonradiative decay and photoisomerization, enabling efficient photorelease of the neuroprotective agent valproic acid. The versatility of PRPG 5d was demonstrated in biologically relevant *in vitro* models, including TDP-43 protein aggregation and Parkinson's disease induced SH-SY5Y neuroblastoma cells. In both extracellular and intracellular neurodegenerative environments, increased viscosity was effectively sensed, triggering light-mediated valproic acid release and subsequent defibrillation. Overall, this work establishes coumarin fluorogen-based PRPGs as a promising platform for viscosity-guided, spatiotemporally controlled drug release, offering potential applications in the diagnosis and targeted therapy of neurodegenerative diseases.

## Abstract 2

Exploring viscosity sensitivity of π-extended coumarin fluorogen-based PRPGs for precise photorelease of valproic acid: detection and defibrillation of TDP-43 Aggregation.

## Graphical abstract

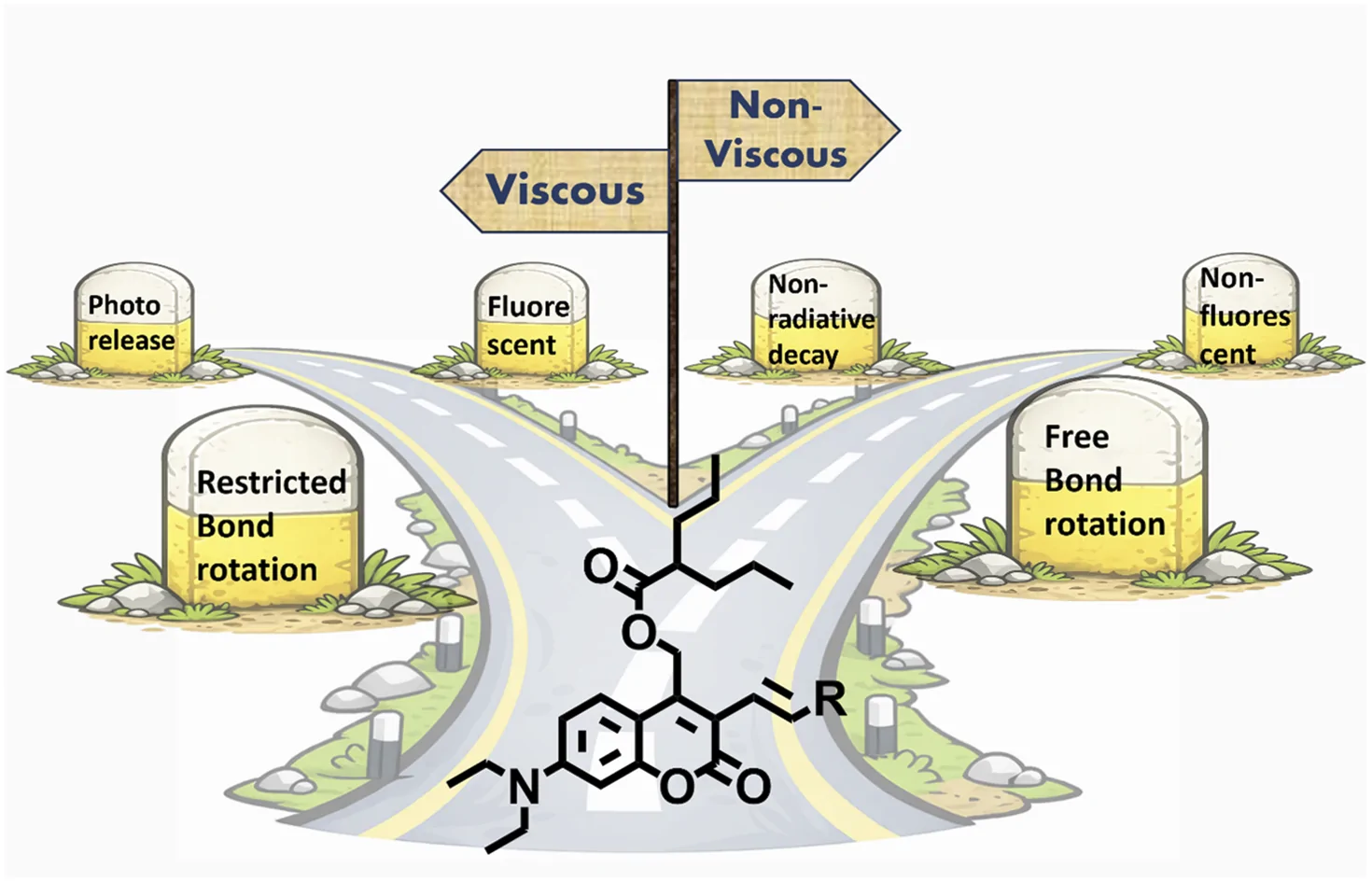

## Introduction

The viscosity of the intracellular microenvironment reveals cellular conditions by reflecting changes in molecular crowding, metabolic activity, and structural organization. The increase in viscosity in the cellular microenvironment causes dysfunction, leading to diseases such as cancer, neurodegenerative diseases, and inflammatory diseases.^1^ Therefore, developing viscosity-sensitive organic fluorophores capable of controlled and precise release of bioactive molecules can enable targeted and adaptive therapeutic interventions for diagnosis and treatment.^2,3^ Neurodegenerative diseases such as Alzheimer's disease, Parkinson's disease, Amyotrophic lateral sclerosis (ALS), and frontotemporal lobar degeneration (FTLD) are caused by misfolding or aggregation of respective proteins like Amyloid-beta (Aβ),^4^ tau,^5^ Alpha-synuclein (α-syn),^6^ TAR DNA-binding protein (TDP).^7^ This protein misfolding or aggregation changes the cellular microenvironment, resulting in restricted flow of cellular fluid and causing an enhancement of the viscosity of the intracellular environment in neuroblastoma cells.^8^ Although alterations in cellular viscosity offer potential for early diagnosis of neurodegenerative diseases, accurately sensing these subtle changes at the cellular level remains a significant challenge. The organic chromophores with a free rotatory bond can be ideal to overcome the challenge. Researchers are now applying the molecular rotor-based fluorophores (RBFs) with twisted intramolecular charge transfer (TICT) property for cellular imaging and viscosity sensing, as this property amplifies the sensitivity of those chromophores towards the microenvironment.^9–12^ RBFs contain an electron donor, an electron acceptor, and a π-extended linker that facilitates the charge transfer. In solvent systems, this extended π-linker can undergo rotation or isomerization (upon photoexcitation) to initiate the charge-transfer process that causes rapid non-radiative decay and quenches fluorescence. In a viscous medium, non-radiative decay is minimized due to restricted bond rotation or isomerization, resulting in enhanced fluorescence.

Understanding the mechanism for incurable neurodegenerative diseases, such as Parkinson's disease, ALS, FTLD and their treatment is crucial for leading a healthy lifestyle.^11^ Sensing the alteration of cellular viscosity change and the precise release of bioactive molecules are promising strategies for both the diagnosis and therapeutic intervention.^3,13^ Photoremovable protecting groups (PRPGs) are light-responsive chromophores that are widely used for the release of drugs or bioactive molecules with high spatiotemporal precision.^14–16^ Hence, fluorogen-based PRPGs can meet these requirements by integrating viscosity–sensitivity with light-triggered, on-demand release of bioactive molecules. This class of PRPGs can sense various environmental factors, such as viscosity,^3^ pH,^17^ oxidative stress,^18,19^*etc*,^20^ and release bioactive molecules precisely on demand. This property facilitates the targeted release of therapeutic agents.

During the design of fluorogen-based PRPGs, certain points should be considered for efficient photorelease of bioactive molecules and biological applicability: (1) the fluorogen-based PRPG should have absorption in the visible to NIR region to minimize cell damage and for better cell and tissue penetration by light,^21–24^ (2) low singlet oxygen generation during the course of photolysis of fluorogen-based PRPGs is crucial to prevent deleterious reactions with singlet oxygen that can compromise photorelease efficiency.^21^ Considering these factors, we selected coumarin as the core moiety for the fluorogen-based PRPG, as it exhibits singlet excited state-based photochemistry,^25^ thereby minimizing the possibility of singlet oxygen generation. We also incorporated an extended π-conjugated moiety and varied the substitution on the other end of the π-conjugated part to achieve a higher absorption wavelength and impart the viscosity-sensing capability.

In this work, we developed a series of coumarin fluorogen-based PRPGs (5a–d) bearing various substitutions tethered to a π-extended linker to evaluate variations in viscosity-sensing capability (Fig. 1).^26^ We varied the substitution by tuning the electron-donating and accepting properties of the tethered substitution as follows: 5a with a pyridine moiety (negligible electron donation/acceptance), 5b with an *N*,*N*-dimethylaniline moiety (electron-donating), and 5c and d with benzothiazole and benzothiazolium moieties (increasingly electron-accepting). We conducted a comprehensive photophysical study to determine viscosity sensitivity, along with a photochemical investigation to compare photorelease behaviour in normal *versus* viscous environments and were supported by theoretical calculations. To assess the broad applicability of the coumarin fluorogen-based PRPG (5d), we examined the visible-light-triggered *in vitro* release of valproic acid, a well-established neuroprotective agent. The viscosity-sensing capability of the developed coumarin fluorogen-based PRPG (5d) was evaluated under extracellular conditions by incorporating it into aggregated TDP-43 protein, responsible for the neurodegenerative diseases amyotrophic lateral sclerosis (ALS) and frontotemporal lobar degeneration (FTLD).^27–30^ In addition, the intracellular sensitivity of the PRPGs was assessed in the SH-SY5Y neuroblastoma cell line, in which Parkinson's disease-like conditions were induced by incorporating MPP^+^ (1-methyl-4-phenylpyridinium).^31^ These *in vitro* neurodegenerative conditions can cause protein aggregation that increases cellular viscosity, which is detected by the coumarin fluorogen-based PRPGs, thereby triggering the release of valproic acid upon optical excitation.

**Fig. 1:**
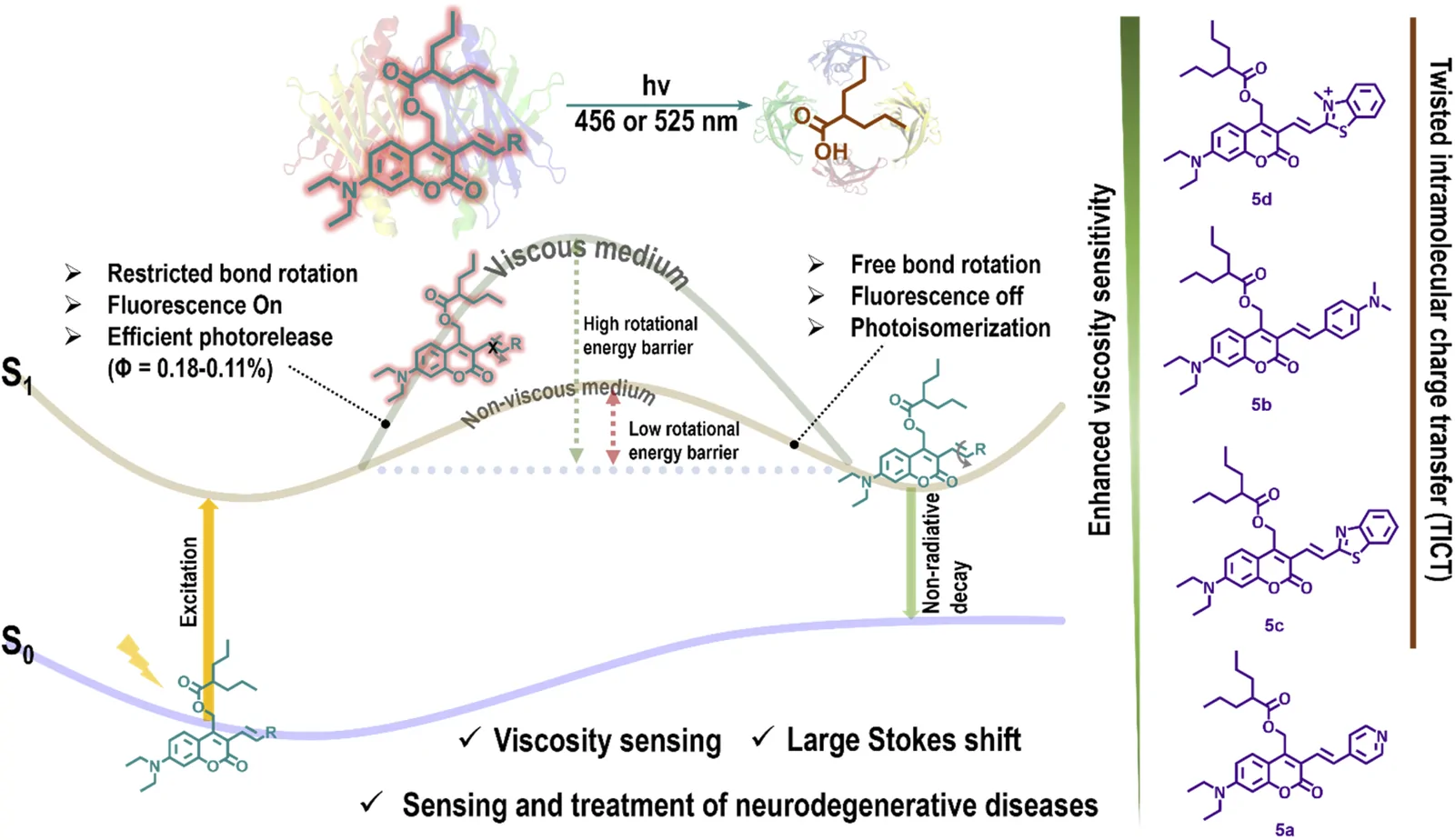
Schematic illustration of coumarin fluorogen-based PRPGs for amyloid sensing and treatment of neurodegenerative diseases.

## Experimental section

### General procedure for synthesis of coumarin fluorogen-based PRPGs (5a–c)

Heck coupling reaction was performed for the synthesis of coumarin fluorogen-based PRPGs (5a–c). To an 8 ml solution of starting material (4) (100 mg, 0.22 mmol) in dry DMF, DIPEA (0.08 ml, 0.44 mmol), Pd(OAc)_2_ (4 mg, 0.02 mmol), and tri(*o*-tolyl)phosphine (18 mg, 0.06 mmol) were added, followed by the addition of RCH = CH_2_ according to the desired PRPG. Then the reaction mixtures were purged with Ar-gas for 45 min. After that, these were refluxed at 110 °C for 2 h. After the completion of the reactions, the organic layer was extracted with EtOAc and washed 2–3 times with brine solution. Then silica gel column chromatography (EtOAc/Hexane) was performed for the purification of products.

#### Synthesis of (*E*)-(7-(diethylamino)-2-oxo-3-(2-(pyridin-4-yl)vinyl)-2*H*-chromen-4-yl)methyl 2-propylpentanoate (5a)

To an 8 ml solution of starting material (4) (100 mg, 0.22 mmol) in dry DMF, DIPEA (0.08 ml, 0.44 mmol), Pd(OAc)_2_ (4 mg, 0.02 mmol), tri(*o*-tolyl)phosphine (18 mg, 0.06 mmol), and 4-vinylpyridine (0.07 ml, 0.66 mmol) were added. Then the reaction mixture was purged with Ar-gas for 45 min. After that, it was refluxed at 110 °C for 2 h. After the completion of the reaction, the organic layer was extracted with EtOAc and washed 2–3 times with brine solution. Then silica gel column chromatography (EtOAc : hexane = 1 : 4) was performed to obtain a solid, red-coloured product. (Yield 41%). ^1^H NMR (500 MHz, CDCl_3_) *δ* 8.56 (d, *J* = 3.9 Hz, 2H), 7.65 (d, *J* = 16.0 Hz, 1H), 7.53 (d, *J* = 9.2 Hz, 1H), 7.43 (d, *J* = 16.0 Hz, 1H), 7.37 (d, *J* = 5.7 Hz, 2H), 6.63 (dd, *J* = 9.2, 2.5 Hz, 1H), 6.51 (d, *J* = 2.5 Hz, 1H), 5.40 (s, 2H), 3.44 (q, *J* = 7.1 Hz, 4H), 2.46–2.39 (m, 1H), 1.64–1.54 (m, 2H), 1.46–1.36 (m, 2H), 1.29–1.20 (m, 10H), 0.82 (t, *J* = 7.3 Hz, 6H). ^13^C NMR (126 MHz, CDCl_3_) *δ* 176.2, 160.7, 155.6, 151.1, 148.9, 146.9, 144.9, 131.3, 126.6, 126.1, 121.4, 116.5, 109.6, 108.4, 97.5, 57.8, 45.4, 45.0, 34.7, 20.8, 14.0, 12.6. HRMS (ESI) *m*/*z*: [M + H]^+^ calcd for C_29_H_36_N_2_O_4_ 476.2675; found 476.2677.

#### Synthesis of (*E*)-(7-(diethylamino)-3-(4-(dimethylamino)styryl)-2-oxo-2*H*-chromen-4-yl)methyl 2-propylpentanoate (5b)

To an 8 ml solution of starting material (4) (100 mg, 0.22 mmol) in dry DMF, DIPEA (0.08 ml, 0.44 mmol), Pd(OAc)_2_ (4 mg, 0.02 mmol), tri(*o*-tolyl)phosphine (18 mg, 0.06 mmol), and *N*,*N*-dimethyl-4-vinylaniline (0.1 ml, 0.66 mmol) were added. Then the reaction mixture was purged with Ar-gas for 45 min. After that, it was refluxed at 110 °C for 2 h. After the completion of the reaction, the organic layer was extracted with EtOAc and washed 2–3 times with brine solution. Then silica gel column chromatography (EtOAc : hexane = 3 : 7) was performed to obtain a solid, red-coloured product. (Yield 38%). ^1^H NMR (500 MHz, CDCl_3_) *δ* 7.45 (d, *J* = 10.6 Hz, 1H), 7.41 (dd, *J* = 7.9, 6.3 Hz, 3H), 7.00 (d, *J* = 16.1 Hz, 1H), 6.69 (d, *J* = 8.8 Hz, 2H), 6.60 (dd, *J* = 9.1, 2.5 Hz, 1H), 6.52 (d, *J* = 2.5 Hz, 1H), 5.38 (s, 2H), 3.42 (q, *J* = 7.0 Hz, 4H), 2.48–2.41 (m, 1H), 1.66–1.60 (m, 2H), 1.47–1.40 (m, 2H), 1.34–1.27 (m, 4H), 1.21 (t, *J* = 7.1 Hz, 6H), 0.86 (t, *J* = 7.3 Hz, 6H). ^13^C NMR (126 MHz, CDCl_3_) *δ* 176.4, 161.6, 154.7, 150.5, 149.9, 140.5, 135.7, 128.1, 126.2, 125.8, 120.2, 116.4, 112.4, 109.1, 108.9, 97.7, 58.8, 45.5, 44.9, 40.5, 34.8, 20.9, 14.1, 12.6. HRMS (ESI) *m*/*z*: [M + H]^+^ calcd for C_32_H_42_N_2_O_4_ 518.3145; found 518.3141.

#### Synthesis of (*E*)-(3-(2-(benzo[*d*]thiazol-2-yl)vinyl)-7-(diethylamino)-2-oxo-2*H*-chromen-4-yl)methyl 2-propylpentanoate (5c)

To an 8 ml solution of starting material (4) (100 mg, 0.22 mmol) in dry DMF, DIPEA (0.08 ml, 0.44 mmol), Pd(OAc)_2_ (4 mg, 0.02 mmol), and tri(*o*-tolyl)phosphine (18 mg, 0.06 mmol) were added, followed by the addition of 2-vinyl-1,3-benzothiazole (106 mg, 0.66 mmol) by dissolving in DMF. Then the reaction mixture was purged with Ar-gas for 45 min. After that, it was refluxed at 110 °C for 2 h. After the completion of the reaction, the organic layer was extracted with EtOAc and washed 2–3 times with brine solution. Then silica gel column chromatography (EtOAc : Hexane = 2 : 3) was performed to obtain a solid, red-coloured product. (Yield 26%). ^1^H NMR (500 MHz, CDCl_3_) *δ* 8.13 (d, *J* = 15.7 Hz, 1H), 7.99 (d, *J* = 8.1 Hz, 1H), 7.85 (d, *J* = 7.9 Hz, 1H), 7.76 (d, *J* = 15.7 Hz, 1H), 7.56 (d, *J* = 9.2 Hz, 1H), 7.45 (t, *J* = 7.4 Hz, 1H), 7.36 (d, *J* = 7.7 Hz, 1H), 6.63 (dd, *J* = 9.2, 2.3 Hz, 1H), 6.51 (d, *J* = 2.3 Hz, 1H), 5.45 (s, 2H), 3.44 (q, *J* = 7.1 Hz, 4H), 2.47–2.41 (m, 1H), 1.64–1.58 (m, 2H), 1.42 (td, *J* = 7.4, 2.3 Hz, 2H), 1.31–1.25 (m, 4H), 1.23 (t, *J* = 7.1 Hz, 6H), 0.82 (t, *J* = 7.3 Hz, 6H). ^13^C NMR (126 MHz, CDCl_3_) *δ* 176.2, 167.7, 160.4, 155.7, 154.3, 151.2, 145.5, 134.9, 128.9, 127.1, 126.7, 126.4, 125.3, 123.2, 121.6, 116.0, 109.6, 108.4, 97.5, 57.7, 45.4, 45.1, 34.7, 20.8, 14.1, 12.6. HRMS (ESI) *m*/*z*: [M + H]^+^ calcd for C_31_H_36_N_2_O_4_S 532.2396; found 532.2395.

#### Synthesis of (*E*)-2-(2-(7-(diethylamino)-2-oxo-4-(((2-propylpentanoyl)oxy)methyl)-2*H*-chromen-3-yl)vinyl)-3-methylbenzo[*d*]thiazol-3-ium (5d)

To the 10 ml solution of PRPG (5c) (100 mg, 0.19 mmol) in dry DCM, Meerwein's salt (83 mg, 0.56 mmol) was added, and the reaction mixture was left at room temperature for 2 h. Then silica gel column chromatography (methanol : DCM = 1 : 19) was performed to obtain a solid, violet-coloured product. (Yield 21%). ^1^H NMR (500 MHz, CDCl_3_) *δ* 8.45 (d, *J* = 14.9 Hz, 1H), 8.35 (d, *J* = 14.9 Hz, 1H), 8.07–8.00 (m, 2H), 7.84–7.76 (m, 2H), 7.66 (t, *J* = 7.7 Hz, 1H), 6.76–6.71 (m, 1H), 6.47 (d, *J* = 1.8 Hz, 1H), 5.58 (s, 2H), 4.27 (s, 3H), 3.50 (q, *J* = 7.0 Hz, 4H), 2.43–2.38 (m, 1H), 1.54 (dd, *J* = 13.6, 7.9 Hz, 2H), 1.41–1.37 (m, 2H), 1.28–1.25 (m, 6H), 1.17 (d, *J* = 7.4 Hz, 4H), 0.78 (t, *J* = 7.3 Hz, 6H). ^13^C NMR (126 MHz, CDCl_3_) *δ* 176.1, 172.8, 159.9, 157.1, 153.4, 152.4, 142.7, 142.1, 130.2, 129.1, 128.8, 127.8, 123.5, 116.5, 113.7, 111.9, 111.0, 109.0, 97.2, 77.4, 57.3, 45.5, 45.2, 36.2, 34.5, 20.7, 14.1, 12.7. HRMS (ESI) *m*/*z*: [M]^+^ calcd for C_32_H_39_N_2_O_4_S^+^ 547.2631; found 547.2632.

### Photochemical studies of 5d

1.8 mg of the Coumarin fluorogen-based PRPG 5d was dissolved in 0.7 ml of NMR solvents (ACN-d_3_ and D_2_O). The solution was irradiated with green LED (525 ± 5 nm) for 4 h, and the photorelease was monitored using time-dependent proton NMR. The photolysis was also monitored by absorption spectroscopy after irradiation of a 0.01 mM 5d solution at different time intervals. The photorelease quantum yield was calculated in a relative method by proton NMR (decrease of ester –CH_2_ peak intensity) using 1,2-dichloroethane as an internal standard (1 equivalent) and fulgide as actinometer.

### Aggregation of TDP-43 protein

4 µL of TDP-43 Protein was diluted to a 500 µL solution of PBS buffer of pH 7.4 to get the final concentration of 1 µM. Then the resulting solution was stirred at room temperature for 9 days. The aggregation of the protein was confirmed by Circular dichroism (CD) and TEM analysis.

### Cell culture

SH-SY5Y cells (Kindly provided by Dr Dibyendu Samanta, Indian Institute of Technology Kharagpur, India) were cultured in DMEM supplemented with 10% fetal bovine serum (FBS) and penicillin–streptomycin and maintained in a humidified incubator at 37 °C with 5% CO_2_. Upon reaching 70–80% confluency, cells were differentiated by treatment with retinoic acid (10 µM) for 7 consecutive days. Differentiated cells were then exposed to MPP^+^ (1 mM) for 24 h to induce a Parkinson's disease model.

### Cellular internalization study of coumarin fluorogen-based PRPG 5d in SH-SY5Y

SH-SY5Y cells and MPP^+^ treated SH-SY5Y cells were seeded at a density of 5000 cells per well one day before the treatment. Then the cells were treated with 5d (100 µM) for 5 h. After treatment, cells were subsequently washed with 1× PBS, and confocal imaging was performed before and after irradiation for 40 min with green LED (525 ± 5 nm).

### Cell viability study

Cell viability of the coumarin fluorogen-based PRPGs (5a–d) and valproic acid was measured using the 3-(4,5-dimethylthiazol-2-yl)-2,5-diphenyltetrazolium bromide (MTT) assay. 1.5 × 10^7^ cells per ml were seeded in a 96-well plate overnight. Then, the compounds were treated at different concentrations for 24 hours and were further exposed to LED light irradiation to obtain data with and without light exposure. Following treatment, MTT solution (5 mg mL^−1^) was added and incubated for 3 h. The resulting formazan crystals were dissolved in a 1 : 1 mixture of dimethyl sulfoxide (DMSO), and absorbance was measured at 595 nm using a microplate reader. Cell viability was expressed as a percentage using the formula: % viability = [(A595 (treated cells) − background)/(A595 (untreated cells) − background)] × 100.

## Results and discussion

To explore how viscosity responds to structural variation, we systematically varied substitutions connected with the π-linker with developed coumarin fluorogen-based PRPGs and examined how each modification influenced the viscosity sensitivity. To synthesise the coumarin fluorogen-based PRPGs (5a–d) (Scheme 1), we started with the bromination of commercially available coumarin aldehyde (1). Then the aldehyde group of brominated coumarin (2) was reduced to the corresponding alcohol using sodium borohydride, yielding compound (3). The esterification of the alcohol (3) by the EDC-coupling reaction with valproic acid gave the coumarin ester (4). Finally, Heck coupling reaction of coumarin ester (4) with different vinyl substitutions resulted in various substituted coumarin fluorogen-based PRPGs (5a–c). The coumarin fluorogen-based PRPG (5d) was prepared by *N*-methylation of 5c using Meerwein's salt. All the detailed synthetic methods and characterization of all the compounds using NMR spectroscopy and HRMS are provided in the SI (Fig. S1–S14).

**Scheme 1:**
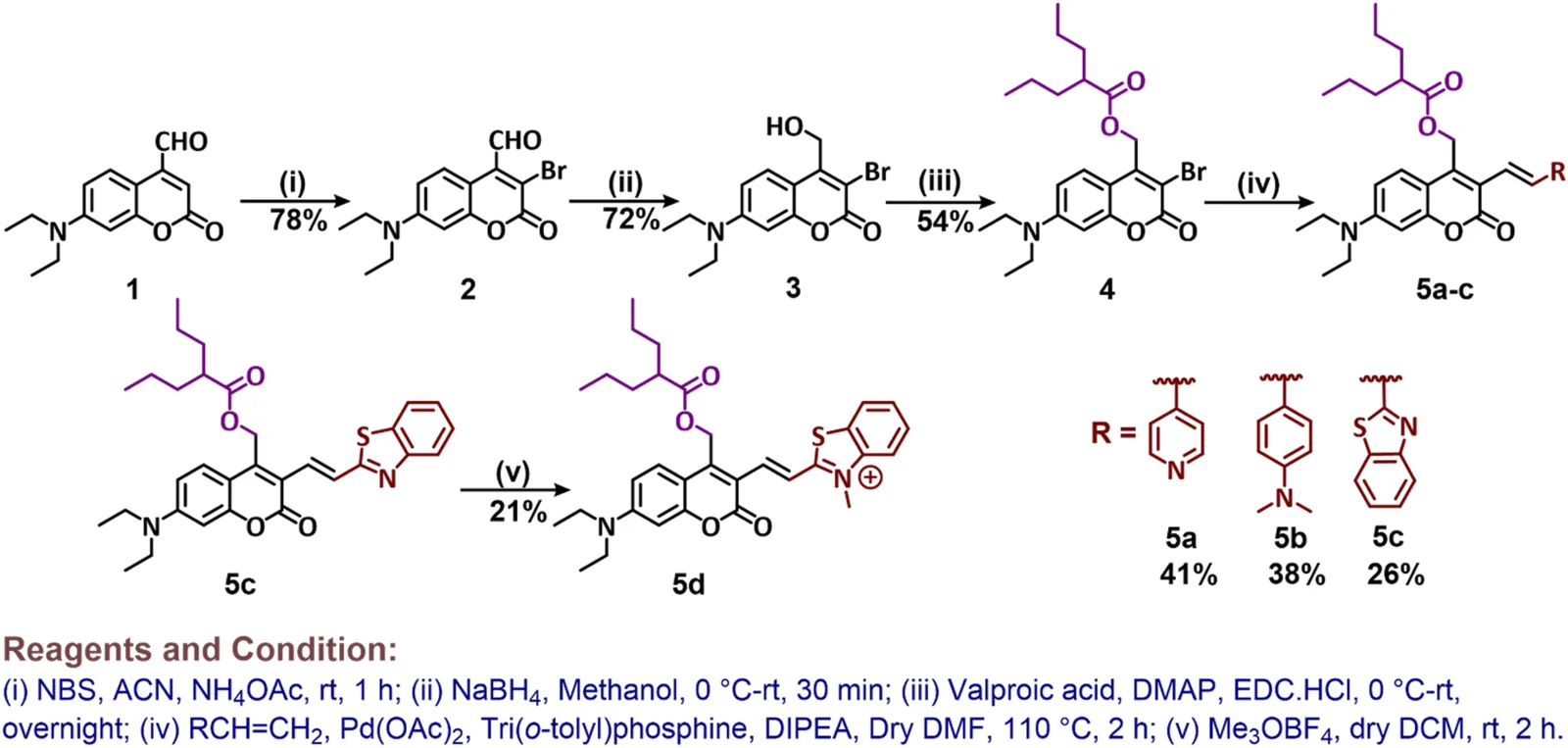
Synthetic scheme of coumarin fluorogen-based PRPGs (5a–d).

Once the synthesis was completed, we recorded the electronic absorption and emission spectra of coumarin fluorogen-based PRPGs (5a–d) in solvents with various polarity indices, *e.g.*, toluene, THF, ACN, DMSO, methanol, and H_2_O. In acetonitrile, PRPGs 5a–c showed absorption maxima in the range of 437–458 nm (Fig. S15a), whereas the presence of the benzothiozolium group, having a better electron accepting capability, resulted in a red-shifted absorption maximum for 5d (560 nm) (Fig. 2a). Also, the theoretical findings for 5d revealed improved charge separation between HOMO and LUMO, resulting in a decrease in the HOMO–LUMO energy gap, which causes this bathochromic shift (Fig. S37). The steady-state emission spectra in acetonitrile were obtained by exciting at the respective absorption maxima (Fig. 2a and S15c), and the emission maxima were found at 517 nm, 562 nm, 538 nm, and 653 nm for coumarin fluorogen-based PRPGs 5a–d, respectively, with large Stokes shifts ranging from 80–119 nm. All the photophysical data are tabulated in Table S1. These large Stokes shifts suggest the possibility of a twisted intramolecular charge transfer (TICT) process arising from D–π–D or D–π–A skeletons in the coumarin fluorogen-based PRPGs (5a–d). To further investigate the TICT property of 5a–d, solvent-dependent emission spectra were recorded across a range of solvent polarities (Fig. 2b and S16) and found that all the other coumarin fluorogen-based PRPGs (5b–d), except 5a, showed a decrease in emission intensity with increasing solvent polarity, as polar solvents stabilise the charge-transfer state.

**Fig. 2:**
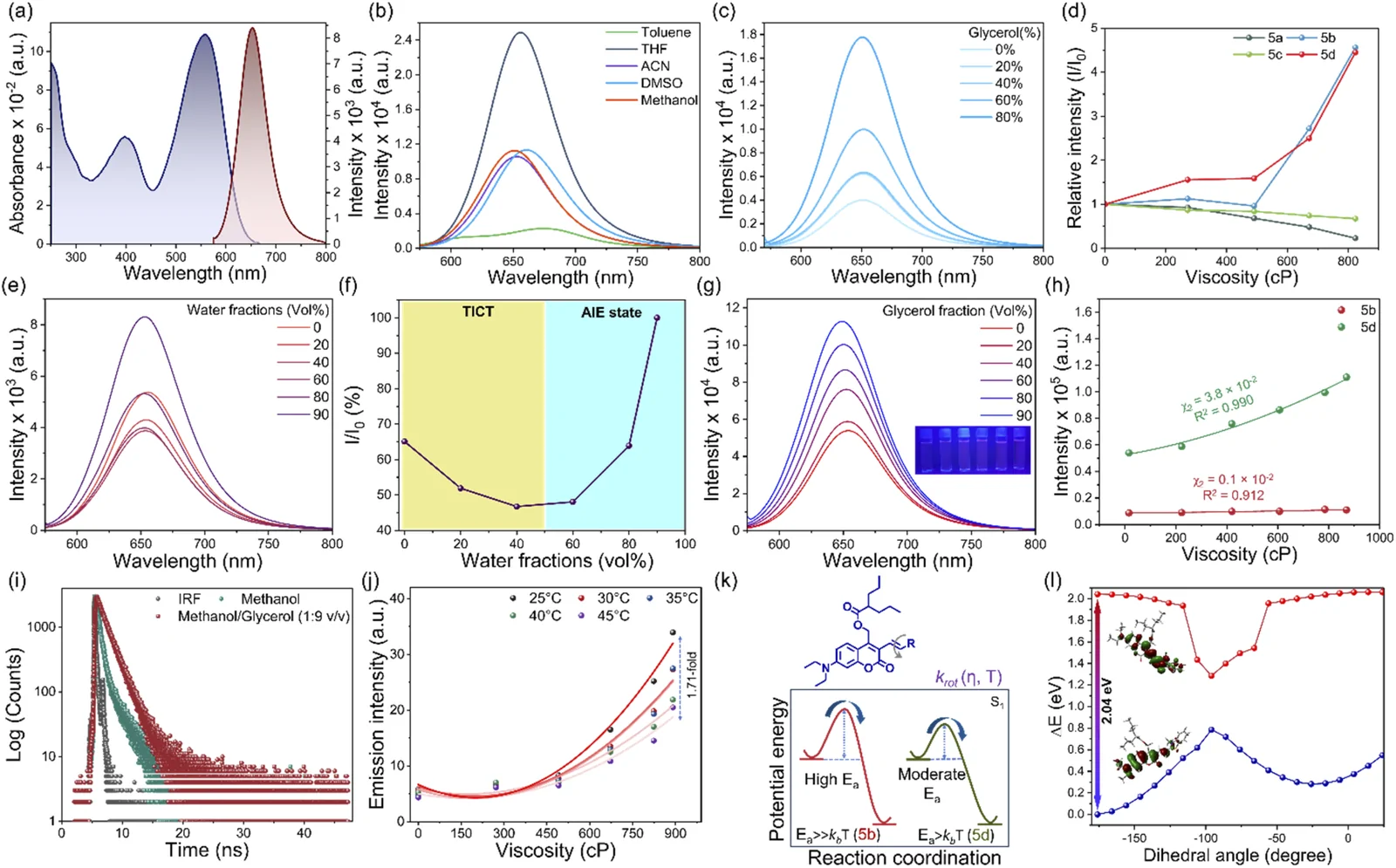
(a) Absorption and emission (*λ*_ex_ = 560 nm) spectra of coumarin fluorogen-based PRPG 5d in ACN. (b) Emission spectra of 5d in different solvents with various polarities. (c) Emission spectra of coumarin fluorogen-based PRPG 5d with varying methanol–glycerol percentage. (d) Comparison of relative fluorescence intensities among coumarin fluorogen-based PRPGs (5a–d) with an increase in viscosity. (e) Emission spectra of coumarin fluorogen-based PRPG 5d as the water percentage in the ACN/water mixture increases. (f) Plot of Relative fluorescence intensity (*I*/*I*_0_) *versus* water fraction for 5d, where I_0_ is the fluorescence intensity in pure ACN. (g) Emission spectra of coumarin fluorogen-based PRPG 5d with varying ethylene glycol–glycerol percentage. (h) Quantitative analysis of viscosity sensitivity for coumarin fluorogen-based PRPGs 5b and 5d. (i) Time correlated single photon counting (TCSPC) decay profile of coumarin fluorogen-based PRPG 5d at an excitation wavelength of 510 nm in methanol and methanol–glycerol (1 : 9 v/v). (j) Plot of emission intensity *versus* viscosity of the solvent (methanol–glycerol mixture) for 5d with temperature variation. The final probe concentration for all experiments was 10 µM. (k) Representative diagram of the rotational energy barrier models based on temperature *vs.* emission intensity in solvents with different viscosities. (l) Variation of energy gap between ground and first excited (S_1_) states for 5d with respect to the dihedral angle between coumarin and benzothiazole moiety as calculated using M062X functional and TZVP basis set (Blue-ground state, Red-excited state).

Next, to explore the viscosity sensitivity, we recorded the emission spectra of coumarin fluorogen-based PRPGs (5a–d) in methanol–glycerol mixed solvent systems by varying the glycerol percentage (0%, 20%, 40%, 60%, and 80%) (Fig. 2c and S17). The results showed a 4.4–4.7 times increase in emission intensities for coumarin fluorogen-based PRPGs 5b and 5d with increasing viscosity of the solvent medium, whereas for 5a and 5c, the emission intensities gradually decreased (Fig. 2d). These results imply that the coumarin fluorogen-based PRPGs 5b and 5d have the viscosity-sensing capability. A viscous medium caused restriction in molecular rotation that hindered faster non-radiative decay processes, resulting in enhanced emission. To investigate the restriction in molecular rotation, we carried out emission spectral measurements for coumarin fluorogen-based PRPGs with varying acetonitrile–water fraction (Fig. 2e and S19). The investigation for 5d showed that with a gradual increase in water content up to 40%, the emission intensity gradually decreased. This indicates dominance of the TICT state with an increase in solvent polarity. After that, with an increase in the water percentage, the compound (5d) started to aggregate, resulting in an enhancement in the emission intensity (Fig. 2e). This discernible trend clearly implies the existence of two competitive states, namely the TICT and the aggregated state. Initially, at water fractions up to 40%, the TICT state predominates, resulting in quenched emission intensity. However, beyond 40% water fraction, restriction of the molecular rotation stabilized the aggregated state, thereby enhancing the emission intensity (Fig. 2f). The above results were also supported by TCSPC data, where in methanol the average excited state lifetime for 5b and 5d were 0.36 ns and 0.54 ns, respectively. However, in methanol–glycerol (1 : 9 v/v), the average excited state lifetime for 5b and 5d were found to be 0.81 ns and 1.31 ns, respectively (Fig. 2i, S20 and Table S4). The lower excited state lifetime in polar solvent (*e.g.* methanol) confirms the existence of the TICT state, whereas in a methanol–glycerol mixture, a relatively higher excited state lifetime suggests the dominance of the aggregated state in a viscous medium. Further to justify the experimental findings for the TICT state, we carried out TD-DFT calculations to investigate the variation in the energy gaps between ground and first excited (S_1_) states with changes in the dihedral angle between the coumarin and benzothiazolium moieties for 5d using the M062X functional^33^ and TZVP basis set^34^ (Fig. 2l). The calculation revealed an energy gap of 2.04 eV when the dihedral angle was −175.5°. This energy gap resulted in an absorption maximum of ∼600 nm, which matches the experimental one (*λ*_max_ ∼ 560 nm).

Then, to compare viscosity sensitivity between coumarin fluorogen-based PRPGs 5b and 5d, we measured fluorescence intensity variation in binary mixtures of ethylene glycol and glycerol with viscosity indices ranging from 16 to 870 cP (Fig. 2g and S18). Here, we chose an ethylene glycol and glycerol binary mixture, having closely matched dielectric constants to minimize the polarity effects. An increase in glycerol percentage leads to an increase in fluorescence intensity for coumarin fluorogen-based PRPGs 5b and 5d, and *χ*_2_ values were derived from the second-order regression plot (Fig. 2h) to compare the viscosity sensitivity.^32^ The higher *χ*_2_ value indicates a greater change in fluorescence intensity as a function of viscosity. A higher *χ*_2_ value for 5d (3.8 × 10^−2^) in comparison to 5b (0.1 × 10^−2^) signifies greater viscosity sensitivity for 5d, which showed a 2.06-fold enhancement in fluorescence intensity as a function of change in viscosity, whereas 5b showed only 1.25-fold enhancement. These results showed that higher fluorescence turn-on for 5d was caused by suppression of the TICT state.

The above experiments indicate that increased viscosity suppresses the non-radiative decay pathway by slowing down both bond rotation and photoisomerization. To further validate our experimental findings, we conceptualized two models: (1) *E*_a_ ≫ *k*_*b*_T, and (2) *E*_a_ > *k*_*b*_T, for 5b and 5d, respectively (Fig. 2k). To clarify this, we measured the change in fluorescence intensity in methanol–glycerol (1 : 9 v/v) with the variation of temperature (25–45 °C). Here, 5d showed a 1.71-fold change in fluorescence intensity (Fig. 2j), indicating a moderate rotational energy barrier (*E*_a_ > *k*_b_*T*) for photoisomerization process. However, a 1.85-fold change in the emission intensity was observed for 5b (Fig. S21), which indicates a higher rotational barrier (*E*_a_ ≫ *k*_b_*T*) for photoisomerization. The high *E*_a_ value resulted in retention of greater fluorescence for 5b by restricting the photoisomerization and inhibiting non-radiative decay even in a non-viscous medium. This was also supported by relatively higher fluorescence quantum yield for 5b (0.14) compared to 5d (0.01), measured in ACN (Table S1). Hence, 5d with a moderate *E*_a_ value exhibited better viscosity sensitivity than 5b, where 5d showed 1.65 times higher fluorescence turn-on in a viscous medium.

After exploring the viscosity sensitivity of all the coumarin fluorogen-based PRPGs (5a–d), we monitored their photorelease capability of valproic acid by proton NMR (details are provided in the SI). The photouncaging ability of coumarin fluorogen-based PRPGs (5a–d) was monitored using a time-dependent ^1^H NMR study (Fig. S24) and HRMS (Fig. S25) under irradiation with blue and green LEDs (456 ± 10 nm and 525 ± 10 nm, respectively). Among all, we chose green-light-activated 5d as a representative example for a detailed study of photorelease. The time-dependent proton NMR study for 5d, upon green light irradiation, resulted in a shift of the ester –CH_2_ peak at 5.69 ppm to 4.47 ppm, which indicates the formation of alcohol photoproduct (5d(PP)). Also, the release of valproic acid was confirmed by the shift of peaks at 2.59 ppm, 1.55 ppm, and 1.22 ppm to 1.33 ppm and 0.93 ppm, which matched with the peaks of free valproic acid (Fig. 3a). Additionally, in the HRMS spectrum of the photolysis mixture (Fig. S26b), the found *m*/*z* value 421.1583 confirmed the formation of the corresponding alcohol photoproduct (5d(PP)). Further, the photoproduct (5d(PP)) was isolated and characterized by proton NMR spectroscopy (Fig. S26a). The photorelease quantum yield for coumarin fluorogen-based PRPGs was determined using 1,2-dichloroethane as an internal standard in the NMR spectra (Fig. S24) and tabulated in Table S5 (relative method: Fulgide used as actinometer, Fig. S22 and S23). It was found that the 5d released the valproic acid with a photorelease quantum yield of 0.11%. We also checked the spatiotemporal control over the photorelease for 5d by performing the ‘ON–OFF’ study and confocal imaging by localized irradiation (Fig. S31).

**Fig. 3:**
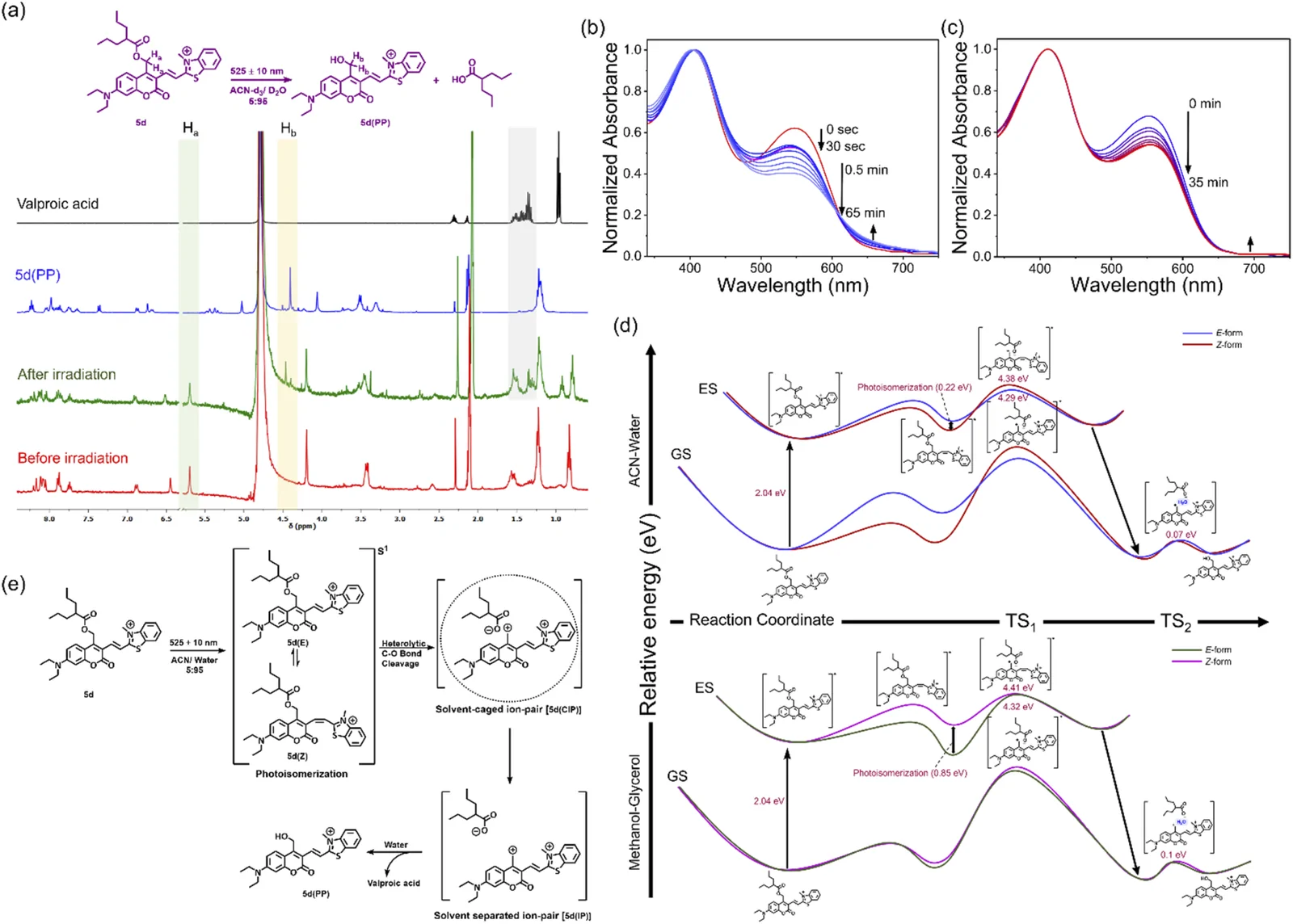
(a) Photorelease monitoring of coumarin fluorogen-based PRPG 5d by time-dependent proton NMR (acetonitrile-d_3_/D_2_O 1 : 9 v/v) when irradiated with 525 ± 10 nm LED. Photolysis monitoring of 5d by absorption spectroscopy in (b) ACN–H_2_O (1 : 19 v/v), and (c) MeOH–glycerol (1 : 9 v/v), when the final concentration of the probe was 10 µM. (d) Approximate potential energy landscape for photolysis of 5d in ACN–water (non-viscous) and methanol–glycerol (viscous) calculated using M062X functional and TZVP and SV basis sets. (e) Plausible photorelease mechanism of coumarin fluorogen-based PRPG 5d.

After confirming the photorelease, we investigated the effect of viscosity on the photorelease process by monitoring photolysis using absorption spectroscopy (Fig. 3b and c and S28–S30) at different solvent systems (ACN, ACN/water (1 : 19), and methanol/glycerol (1 : 9)). Again, we used 5d as a representative to get a better understanding of the effect of viscosity on photorelease. In absorption spectra, the *E*-form (5d(E)), *Z*-form (5d(Z)), and photoproduct (5d(PP)) should have the same absorption maximum at 560 nm with different absorbance values.^26,35^ Thus, during the course of photolysis or photoisomerization, a change in intensity was observed at the 560 nm absorption band, where, for photoisomerization the change will be faster. First, when the photolysis of 5d was performed in ACN (Fig. S28d), only a sharp decrease in the absorption intensity at 560 nm within 30 s was caused by photoisomerization. However, in ACN/water (1 : 19, v/v) (Fig. 3b), a sharp decrease in intensity at the 560 nm absorption band within 30 s is followed by a gradual decrease over 65 min during irradiation, indicating the occurrence of both photoisomerization and photouncaging processes. On the other hand, in methanol/glycerol (1 : 9 v/v), a gradual decrease in absorption intensity during irradiation indicates the photouncaging process. To support the experimental results, we did a theoretical calculation of relative energies for photoisomerization and photouncaging of 5d using the M062X functional^33^ and TZVP and SV basis sets^34^ for both the ACN/water and methanol/glycerol solvent systems (Table S6 and Fig. S38). The findings suggested that in ACN/water, the photoisomerization process has an energy gap of 0.22 eV. Whereas this energy gap in methanol/glycerol is 0.85 eV (Fig. 3d). Hence, the photoisomerization for ACN/water was more favourable. Thus, both the experimental and theoretical results suggested that an efficient photorelease of 5d in a viscous medium for restriction in bond rotation.

After confirming the photorelease and the effect of viscosity on photolysis, we looked into the photorelease mechanism for coumarin fluorogen-based PRPG 5d. First, the quenching study was performed using *N,N′*-Di-*n*-octyl-3,4,9,10-perylenetetracarboxylic diimide as a singlet state quencher and potassium sorbate as a triplet state quencher to check whether the photochemistry goes *via* the singlet or triplet excited state (Fig. S27). It was observed that in the presence of a triplet quencher, there was no change in the photolysis rate. However, in the presence of a singlet quencher, the photoisomerization and photouncaging rate became 10-times slower. This result indicates that the photochemistry goes *via* the singlet excited state. Then we performed the photolysis in a methanol/water (1 : 9 v/v) solvent system. The HRMS of the photolysis mixture confirmed the formation of the methoxy photoproduct ((5d(PP′)) (Fig. S26c). This result provided evidence of the formation of the ion pair *via* heterolytic cleavage in the excited state during the photolysis. Based on experimental evidence, literature survey, and theoretical calculations,^25,26,35,36^ we propose the possible photorelease mechanism for 5d as follows (Fig. 3e): initially, upon green light irradiation, 5d gets excited to its singlet excited state. There, it can undergo *E*–*Z* photoisomerization. Then, in the excited state, one heterolytic cleavage will take place between the ester C–O to form the ion-pair. This ion pair will further relax back to the ground state and be caged by solvent to give a solvent-caged ion-pair (5d(CIP)). After that, this solvent-caged ion-pair will get separated by solvent molecules to form a solvent-separated ion-pair (5d(IP)). Further, this solvent-separated (5d(IP)) ion-pair was trapped by solvent (H_2_O) to get the alcohol photoproduct (5d(PP)) and release the valproic acid.

After exploring detailed photophysical and photochemical properties for all coumarin fluorogen-based PRPGs, we found that viscosity sensitivity and green-light activation of 5d make it suitable for biological applications. So, we designed an *in vitro* experiment centred on the intriguing TAR DNA-binding protein 43 (TDP-43). The abnormal aggregation of TDP-43 causes several neurodegenerative diseases, such as amyotrophic lateral sclerosis (ALS), frontotemporal lobar degeneration (FTLD), *etc.* The fibril formation by aggregation of TDP-43 enhances viscosity in the cellular environment,^37^ which can be sensed by coumarin fluorogen-based PRPG 5d, and the release of valproic acid can cause defibrillation (Fig. 4a). To mimic the cellular fibrillization, we aggregated TDP-43 in PBS buffer solution (pH 7.4),^29^ and the aggregation was further characterised by circular dichroism (CD), thioflavin T fluorescence kinetic assay (Fig. S32a), DLS experiment (Fig. S32c), and TEM (Fig. S32b). To further investigate the sensing capability, the coumarin fluorogen-based PRPG 5d was incorporated into the aggregated solution. Notably, 5d showed a 1.6-fold enhancement of emission intensity (Fig. 4c), which is comparable with the gold standard thioflavin T (ThT) (1.8-fold) (Fig. 4e). A decrease in the emission intensities was observed for coumarin fluorogen-based PRPGs (5a–c) during protein aggregation (Fig. S33). This trend was consistent for 5a and 5c, but not for 5b, as it is capable of sensing viscosity. The observation can be attributed to the low protein concentration (1 µM), which was insufficient to be detected by 5b, as its viscosity sensitivity is approximately 4-fold lower than 5d. To assess the precision of the fluorometric assay, which can potentially disrupt the aggregated structure, the structural integrity of TDP-43 aggregation was monitored by CD spectrometry in the presence of different concentrations of the coumarin fluorogen-based PRPG 5d. Despite the enhancement of emission intensity, the CD spectrometric pattern remains unchanged, confirming the preservation of the aggregated structure (Fig. S34). To further investigate the direct interactions of 5d with the TDP-43 protein, we performed a molecular docking study of 5d with the human TDP-43 protein using AutoDock Vina (Fig. S40). The result indicated that 5d bound positively with the protein, primarily within or near the U-shaped cavity formed between two arms of the protein (Table S8).

**Fig. 4:**
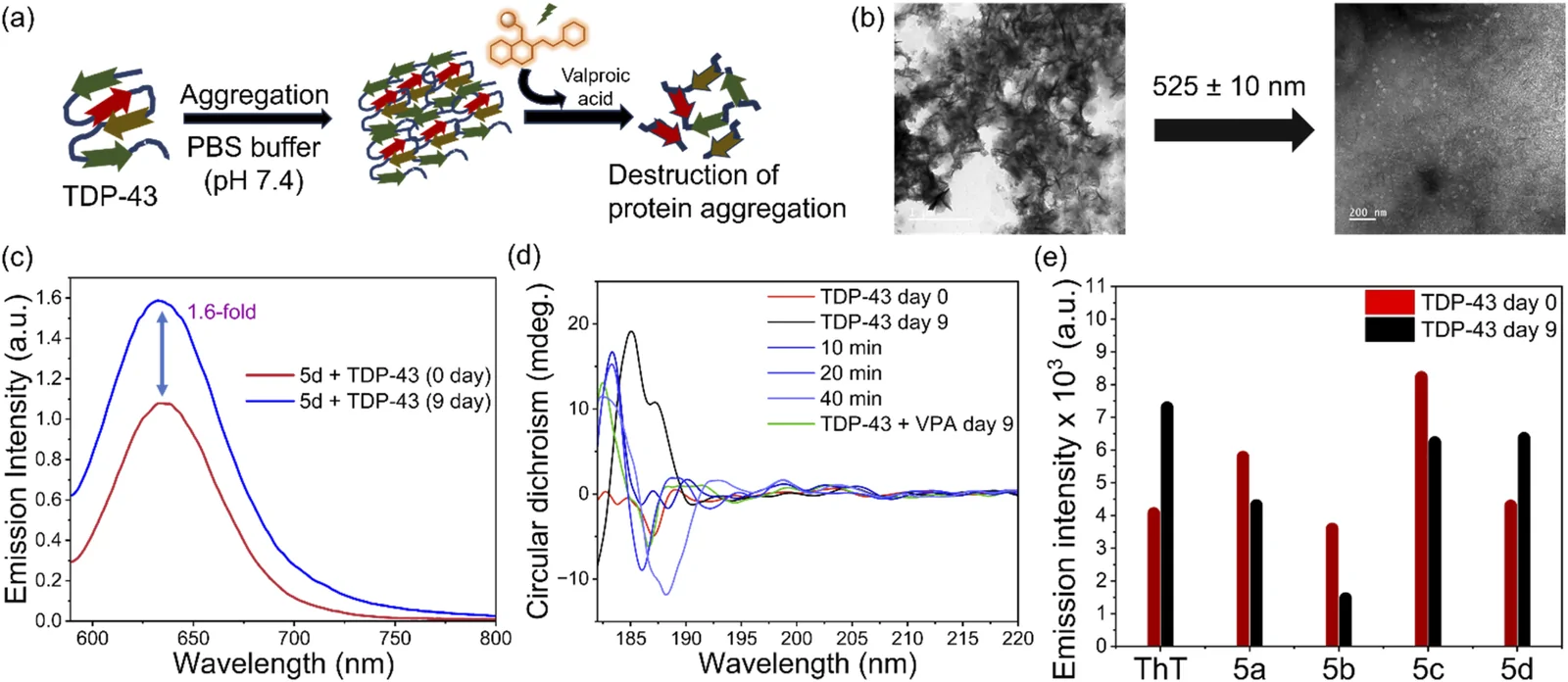
(a) Schematic representation of protein aggregation sensing by coumarin fluorogen-based PRPG. (b) TEM images of aggregated TDP-43 protein before and after irradiation. (c) Emission spectra of coumarin fluorogen-based PRPG 5d (10 µM) in the presence of TDP-43 (1 µM) on day 0 and day 9. 5d was excited at 560 nm. (d) Change in circular dichroism (CD) spectra of aggregated TDP-43 (1 µM) after irradiation with green light (525 ± 10 nm) in the presence of 5d (10 µM). (e) Change in emission intensities of ThT and coumarin fluorogen-based PRPGs (5a–d) for TDP-43 aggregation.

Furthermore, to investigate the defibrillation capability of 5d, five sets of TDP-43 solutions were prepared. In all five sets of solutions, TDP-43 was incubated for 9 days. After 9 days, one set was incubated with valproic acid and three sets with 5d. Then the three sets with 5d were irradiated with 525 ± 10 nm LED light for various time intervals (10, 20, and 40 min), followed by incubation of the samples for another 3 days, and monitored by CD spectrometry (Fig. 4d). Initially, at day 0, the TDP-43 showed a negative signal at around 187 nm in the CD spectrum. After 9 days of aggregation, the CD spectrum showed positive signals at 185 nm with a shoulder at 187 nm, indicating fibrilization of TDP-43. But for the irradiated solutions, the CD spectra began to show a negative signal at 186–188 nm, which resembles the CD spectrum of a TDP-43 solution with valproic acid after 9 days of incubation (Fig. 4d). These results clearly indicate that the controlled release of valproic acid by 5d defibrillates TDP-43. The destruction of the TDP-43 aggregated structure was also confirmed by a TEM image of before and after irradiation (Fig. 4b) and the average fibril length distribution from TEM images, which changed from 254.98 ± 29.56 nm to 83.70 ± 8.74 nm after irradiation with green LED light (525 ± 10 nm) (Fig. S32d). The result was also supported by the decrease in *Z*-average value from 222.4 d.nm to 32.35 d.nm after irradiation (Fig. S32c). The results revealed the bifunctional capability of coumarin fluorogen-based PRPG 5d, which can not only sense TDP-43 aggregation but also defibrillation protein aggregation by releasing valproic acid.

Additionally, to further extend the viscosity sensitivity of coumarin fluorogen-based PRPG 5d to the cellular level, we incubated our PRPG in the SH-SY5Y neuroblastoma cell line. Also, in another set of SH-SY5Y cells, we incubated 1-methyl-4-phenylpyridinium (MPP^+^), a potent neurotoxin,^31^ to model Parkinson's disease-like condition and treated them with 5d. We performed the MTT (3-(4,5-dimethylthiazol-2-yl)-2,5-diphenyltetrazolium bromide) assay on the SH-SY5Y cell line under normal conditions and MPP^+^-treated conditions for the coumarin fluorogen-based PRPGs 5d (Fig. 5b). The assay results showed that, before light irradiation, the IC_50_ values for normal and MPP^+^-treated conditions were >100 µM. After 20 min of light irradiation, the IC_50_ value remained unchanged for normal cells (>100 µM). Whereas, for MPP^+^ treated cells, the IC_50_ value decreased to 98.5 µM. To evaluate whether 5d combined with light irradiation can rescue MPP^+^-induced cytotoxicity and reduce disease-related cellular damage, we performed an MTT assay using three treatment groups. The first group was treated with MPP^+^ alone, the second with 100 µM MPP^+^ and 70 µM 5d, and the third with 100 µM MPP^+^ and 70 µM 5d followed by 30 min of light irradiation.

**Fig. 5:**
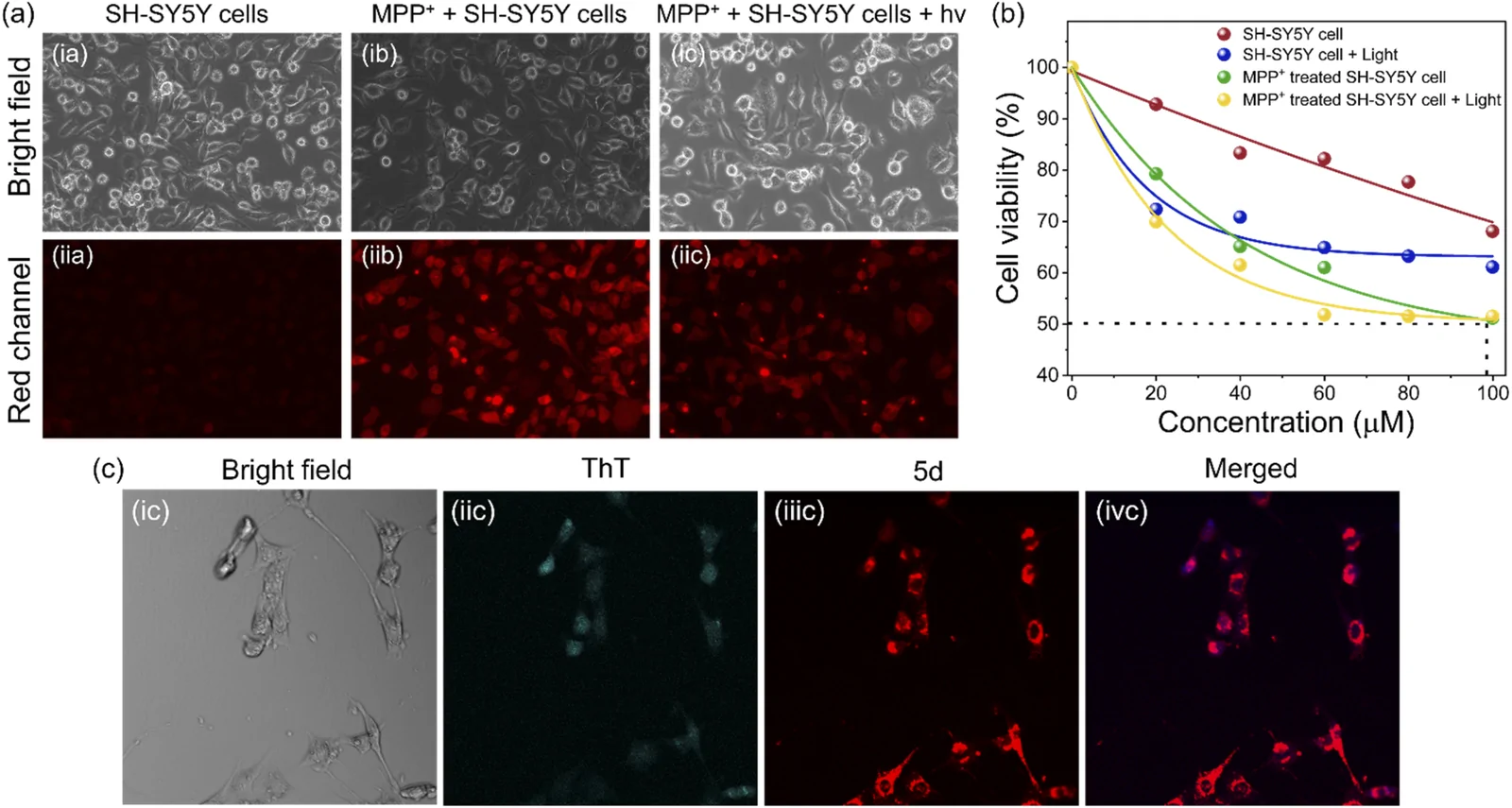
(a) Cellular internalization study of 5d in SH-SY5Y cells, MPP^+^-treated SH-SY5Y cells, and MPP^+^-treated SH-SY5Y cells after irradiation with 525 ± 10 nm LED (Scale bar: 100 µm): (i) bright field images, (ii) cells observed in red channel. (b) Cell viability studies of 5d in the SH-SY5Y neuroblastoma cell line and MPP^+^-treated SH-SY5Y neuroblastoma cell line before and after light irradiation (525 ± 10 nm) (Error limit within ± 10%). (c) Colocalization study of 5d in MPP^+^-treated SH-SY5Y cells. Commercially available Thioflavin T (blue fluorescence) was used as a standard (Scale bar: 50 µm). (i) Bright-field image, (ii) cell incubated with ThT, (iii) cell incubated with 5d, and (iv) merged image.

The MTT assay showed that cells treated with 100 µM MPP^+^ and 70 µM 5d exhibited 74.4% viability before irradiation, which remained essentially unchanged after 30 min of irradiation with a 525 ± 10 nm LED (75.1% viability) (Fig. S35e). These results suggest that valproic acid released from 5d upon light irradiation exerts a neuroprotective effect by inhibiting protein aggregation and reducing disease-related cellular damage.

The selective sensing is also supported by confocal imaging, where coumarin fluorogen-based PRPG 5d showed bright red fluorescence colour in MPP^+^ -treated cells compared to normal SH-SY5Y cells (Fig. 5a). MPP^+^-induced Parkinson's disease-like conditions in SH-SY5Y cells lead to increased intracellular viscosity, primarily due to protein aggregation. Under these conditions, the coumarin fluorogen-based PRPG 5d is able to distinguish between normal and neurodegenerative cells. Furthermore, upon activation by light, it photoreleases valproic acid, promoting defibrillation of protein aggregates, which is reflected as a decrease in red fluorescence intensity in confocal imaging (Fig. 5a). Additionally, a colocalization study using the commercially available viscosity-sensing probe thioflavin T (ThT) supports the intracellular viscosity-guided activation of 5d and defibrillation within the cell (Fig. 5c).

## Conclusion

In summary, we developed a series of coumarin fluorogen-based PRPGs (5a–d) where we chose coumarin as the core moiety with π-extended conjugation, which can sense viscosity using the TICT-AIE mechanism and release valproic acid as a neuroprotective agent in a spatiotemporal controlled manner upon light irradiation. We systematically varied the substituents on the other end of the π-extended conjugated part to explore their viscosity-sensing capability. Detailed experimental and theoretical studies revealed that coumarin fluorogen-based PRPG 5d with *N*-methylbenzothiozolium substitution exhibited higher viscosity sensitivity than 5a–c. Further photochemical studies unveiled the release of valproic acid from 5d with a 0.11% photorelease quantum yield upon exposure to green light. Experimental studies also revealed more efficient photorelease in viscous medium compared to a non-viscous medium for 5d due to restriction in bond rotation or photoisomerization process. Hence, to explore the viscosity sensing capability of 5d in cellular medium, we performed *in vitro* studies with the TDP-43 protein, and Parkinson's disease-like conditions induced in the SH-SY5Y neuroblastoma cell line. In both neurodegenerative models, the increased cellular microenvironmental viscosity was effectively sensed by the developed coumarin fluorogen-based PRPG 5d, resulting in enhanced fluorescence intensity and favouring the light-triggered release of valproic acid for therapeutic intervention. Overall, the developed coumarin fluorogen-based PRPGs offer a versatile platform for viscosity sensing and targeted, light-triggered release of bioactive molecules, with broad potential for the diagnosis and therapeutic intervention in neurodegenerative diseases.

## Author contributions

S. Pal conceptualized and designed the research project, synthesized the photocages, investigated photophysical and photochemical properties, conducted various experiments, and wrote the full manuscript. N. D. P. Singh provided supervision, validated all data, and assisted in manuscript editing. A. P. Chattopadhyay conducted TD-DFT calculations. S. Das carried out *in vitro* studies and contributed to data analysis. N. R. Jana supervised the *in vitro* studies and validated all data. S. Biswas and S. D. Murugan assisted in conducting the experiments.

## Conflicts of interest

There are no conflicts to declare.

## Supplementary Material

SC-017-D6SC03258C-s001

## Data Availability

The data supporting this article have been included as part of the supplementary information (SI). Supplementary information: synthetic details; ^1^H NMR, ^13^C NMR, and HRMS spectra; photophysical properties of coumarin fluorogen-based PRPGs 5a–d; measurement of fluorescence quantum yields; measurement of photochemical quantum yields; photorelease study of coumarin fluorogen-based PRPGs 5a–d; fluorescence lifetimes measurement; characterization of photoproduct; CD spectroscopic measurement; cell imaging; and computational data. See DOI: https://doi.org/10.1039/d6sc03258c.
