# Supplementary material for: Exploring viscosity sensitivity of π-extended coumarin fluorogen-based PRPGs for precise photorelease of valproic acid: detection and defibrillation of TDP-43 aggregation
Source: Chem Sci. 2026 Aug 12;17(38):18167–77. doi: 10.1039/d6sc03258c (PMC13464618; doi:10.1039/d6sc03258c)
Supplement: Supplementary file 1 [file SC-017-D6SC03258C-s001.pdf]

Supplementary Information (SI) for Chemical Science.
This journal is © The Royal Society of Chemistry 2026

Supporting Information

Exploring Viscosity Sensitivity of π-extended Coumarin Fluorogen-based PRPGs for Precise Photorelease of Valproic Acid: Detection and Defibrillation of TDP-43 Aggregation

Subham Pal[a], Sagarika Das[b], Suchhanda Biswas[a], Sri Dinesh Murugan[c], Nihar Ranjan Jana[b], Asoke Prasun Chattopadhyay[d] and N. D. Pradeep Singh*[a]

[a]Department of Chemistry, Indian Institute of Technology Kharagpur, Kharagpur, 721302, India. Email: ndpradeep@chem.iitkgp.ac.in

[b]Department of Bioscience and Biotechnology, Indian Institute of Technology Kharagpur, Kharagpur, 721302, India.

[c]Department of Chemistry, SRM University, Chennai, 603203, India.

[d]Department of Chemistry, University of Kalyani, Kalyani 741235, India.

Table of contents:

Sl. No.	Contents	Page No.
1	**General experimental information**	2
2	**General experimental procedure for the synthesis of compounds 2-4 and coumarin fluorogen-based PRPGs (5a-d), and Spectroscopic data**	2-4
3	**^{1}H and ^{13}C NMR spectra of compounds 2-4 and coumarin fluorogen-based PRPGs 5a-d**	5-11
4	**HRMS of compounds 2-4 and coumarin fluorogen-based PRPGs 5a-d**	12-13
5	**General photophysical characterization methods**	14-18
6	**Determination of incident photon flux (I_0) for LED lamps**	19-20
7	**General photochemical studies**	20-24
8	**TDP-43 protein aggregation and general characterization**	24-26
9	**Biological experiments**	26-27
10	**Computational studies**	27-29
11	**Docking study**	29-30
12	**References**	30
13	**Optimized Cartesian coordinates from DFT calculation**	30-53

1. General experimental information:

The ^{1}H NMR (500 MHz and 400 MHz) spectra were obtained using a BRUKER-AC 500 MHz and a BRUKER-AC 400 MHz spectrometer respectively, with chemical shifts reported in ppm (parts per million) from tetramethylsilane and the solvent resonance as the internal standard. The $CDCl_3$ solvent had a resonance of 7.26 ppm and 1.56 ppm, while DMSO-d_6 had two resonances at 3.31 ppm and 2.48 ppm. The reported data consists of chemical shifts and their respective multiplicities (s = singlet, d = doublet, t = triplet, m = multiplet), and coupling constant *J* (Hz). For the ^{13}C NMR (126 MHz and 100 MHz) spectra, a BRUKER-AC 500 MHz and 400 MHz spectrometer with complete proton decoupling were used. The chemical shifts were reported in ppm from tetramethylsilane, using the solvent resonance as the internal standard. The $CDCl_3$ solvent had a resonance at 77.23 ppm, while DMSO-d_6 had a resonance at 39.96 ppm. The Shimadzu UV-1900i UV/vis spectrophotometer was used to obtain UV-visible absorption spectra, while fluorescence emission spectra were acquired using the Shimadzu RF6000 fluorescence spectrophotometer. HRMS spectra were recorded on a JEOL-Accu TOF JMS-T100L mass spectrometer. The ester conjugates were subjected to photolysis using a Kessil Green LED. Reversed-phase high-performance liquid chromatography (RP-HPLC) was conducted using a mobile phase of acetonitrile and water with a flow rate of 1 mL min^{-1}. Detection was accomplished using UV light at a wavelength of 220 nm. The purification process involved the use of silica gel from Merck with a mesh size of 60-120 and 100-200 mesh. Precoated silica gel 60 F254 TLC sheets from the same manufacturer were utilised for reaction monitoring.

Commercially available solvents, including petroleum ether (PE) and ethyl acetate (EA), were used for workup and column chromatography without additional purification. All reagents were obtained from Sigma Aldrich and utilised without further purification. Human TDP-43 Synthetic Peptide was purchased from ThermoFisher Scientific. HPLC-grade acetonitrile and water were employed for photolysis and HPLC analysis.

2. General experimental procedure for the synthesis of compounds 2-4 and fluorogen-based PRPGs (5a-d) and Spectroscopic data:

Synthesis of 3-bromo-7-(diethylamino)-2-oxo-2H-chromene-4-carbaldehyde (2)

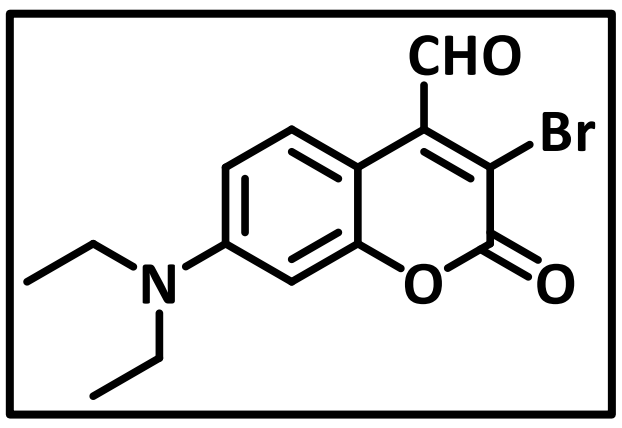

Compound (**2**) was synthesised from commercially available 7-Diethylaminocoumarin-4-carbaldehyde (**1**) according to the reported procedure. To a 20 ml solution of compound (**1**) (1.5 g, 6.12 mmol) in acetonitrile, crystalline N-bromosuccinimide (1.306 g, 7.34 mmol) and a catalytic amount of ammonium acetate were added at room temperature, and the reaction mixture was stirred for 1 h. After the completion of the reaction, the organic layer was extracted with EtOAc, and the product was purified using silica gel column chromatography (EtOAc: Hexane = 1:4) to obtain a solid, dark red-coloured product. (Yield 78%). **^{1}H NMR** (400 MHz, $CDCl_3$) δ 10.38 (s, 1H), 7.97 (d, *J* = 9.3 Hz, 1H), 6.63 (dd, *J* = 9.3, 2.6 Hz, 1H), 6.52 (d, *J* = 2.5 Hz, 1H), 3.42 (q, *J* = 7.1 Hz, 4H), 1.22 (t, *J* = 7.1 Hz, 6H). **^{13}C NMR** (101 MHz, $CDCl_3$) δ 192.9, 158.3, 155.8, 151.1, 142.0, 126.7, 110.0, 109.7, 104.7, 97.5, 45.0, 12.5. HRMS (ESI) m/z: $[M + H]^+$ calcd for $C_{14}H_{14}BrNO_3$ 323.0157; found 323.0148.

Synthesis of 3-bromo-7-(diethylamino)-4-(hydroxymethyl)-2H-chromen-2-one (3)

To a 15 ml solution of compound (**2**) (500 mg, 1.54 mmol) in methanol, $NaBH_4$ (146 mg, 3.86 mmol) was added portion-wise at 0 °C, and the reaction mixture was left at room temperature for 30 min. After completion, the reaction was quenched using a saturated solution of NH_4Cl and the organic layer was extracted with EtOAc. Silica gel column chromatography (EtOAc: Hexane = 2:3) was used to obtain a pure yellow coloured solid product. (Yield 72%). **^{1}H NMR** (500 MHz, $CDCl_3$) δ 7.69 (d, *J* = 9.1 Hz, 1H), 6.66 (d, *J* = 8.3 Hz, 1H), 6.51 (s, 1H), 4.95 (s, 2H), 3.41 (q, *J* = 7.1 Hz, 4H), 1.20 (t, *J* = 7.1 Hz, 6H). **^{13}C NMR** (126 MHz, $CDCl_3$) δ 158.6, 155.3, 151.1, 150.9, 126.7, 109.5, 107.7, 105.3, 97.4, 61.7, 44.9, 12.6. HRMS (ESI) m/z: $[M + H]^+$ calcd for $C_{14}H_{16}BrNO_3$ 325.0314; found 325.0312.

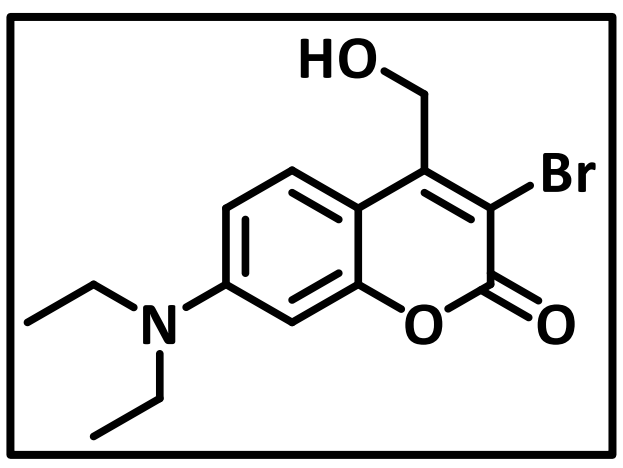

Synthesis of (3-bromo-7-(diethylamino)-2-oxo-2H-chromen-4-yl)methyl 2-propylpentanoate (4)

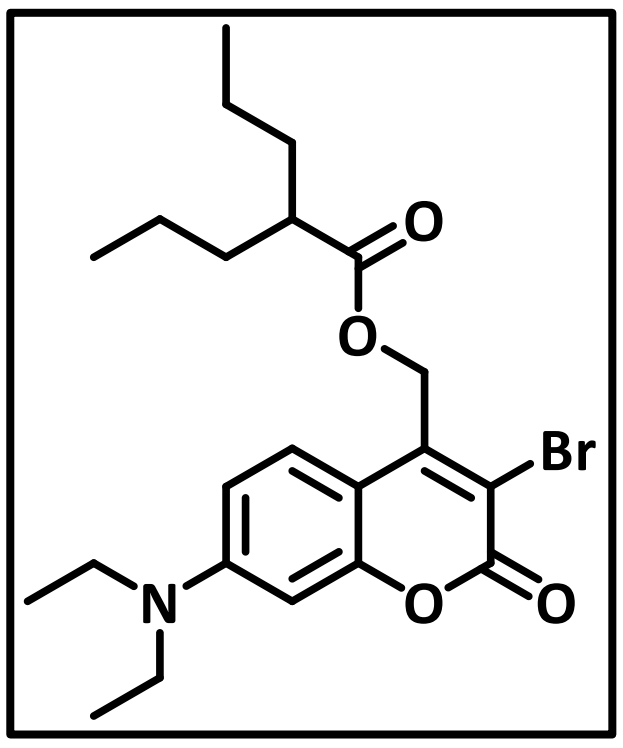

To the 10 ml solution of Valproic acid (106 mg, 0.74 mmol) in dry DCM, a catalytic amount of DMAP was added, followed by the addition of EDC.HCl (169 mg, 0.88 mmol) at 0 °C and stir the mixture for 15 min. Then compound (**3**) (200 mg, 0.61 mmol) was added to the mixture and stirred overnight at room temperature. The organic layer was extracted by DCM and purified using silica gel column chromatography (EtOAc: Hexane = 1:4) to obtain a solid, orangish-yellow coloured product. (Yield 54%). **^{1}H NMR** (500 MHz, $CDCl_3$) δ 7.41 (d, *J* = 9.1 Hz, 1H), 6.58 (d, *J* = 9.1 Hz, 1H), 6.47 (s, 1H), 5.34 (d, *J* = 2.4 Hz, 2H), 3.40 (q, *J* = 7.0 Hz, 4H), 2.44 – 2.35 (m, 1H), 1.63 – 1.52 (m, 2H), 1.45 – 1.35 (m, 2H), 1.30 – 1.22 (m, 4H), 1.19 (dd, *J* = 7.9, 6.2 Hz, 6H), 0.84 (t, *J* = 7.3 Hz, 6H). **^{13}C NMR** (126 MHz, $CDCl_3$) δ 175.9, 158.1, 155.0, 150.8, 147.2, 126.1, 109.4, 107.7, 107.5, 97.5, 61.9, 45.2, 45.0, 34.5, 20.7, 14.0, 12.4. HRMS (ESI) m/z: $[M + H]^+$ calcd for $C_{22}H_{30}BrNO_4$ 451.1358; found 451.1347.

General procedure for the synthesis of coumarin fluorogen-based PRPGs (5a-c)

Heck coupling reaction was performed for the synthesis of PRPGs (**5a-c**). To an 8 ml solution of starting material (**4**) (100 mg, 0.22 mmol) in dry DMF, DIPEA (0.08ml, 0.44 mmol), $Pd(OAc)_2$ (4 mg, 0.02 mmol), and tri(*o*-tolyl)phosphine (18 mg, 0.06 mmol) were added, followed by the addition of $RCH{=}CH_2$ according to the desired PRPG. Then the reaction mixtures were purged with Ar-gas for 45 min. After that, these were refluxed at 110 °C for 2 h. After the completion of the reactions, the organic layer was extracted with EtOAc and washed twice with brine solution. Then silica gel column chromatography (EtOAc/ Hexane) was performed for the purification of products.

Synthesis of (*E*)-(7-(diethylamino)-2-oxo-3-(2-(pyridin-4-yl)vinyl)-2H-chromen-4-yl)methyl 2-propylpentanoate (5a)

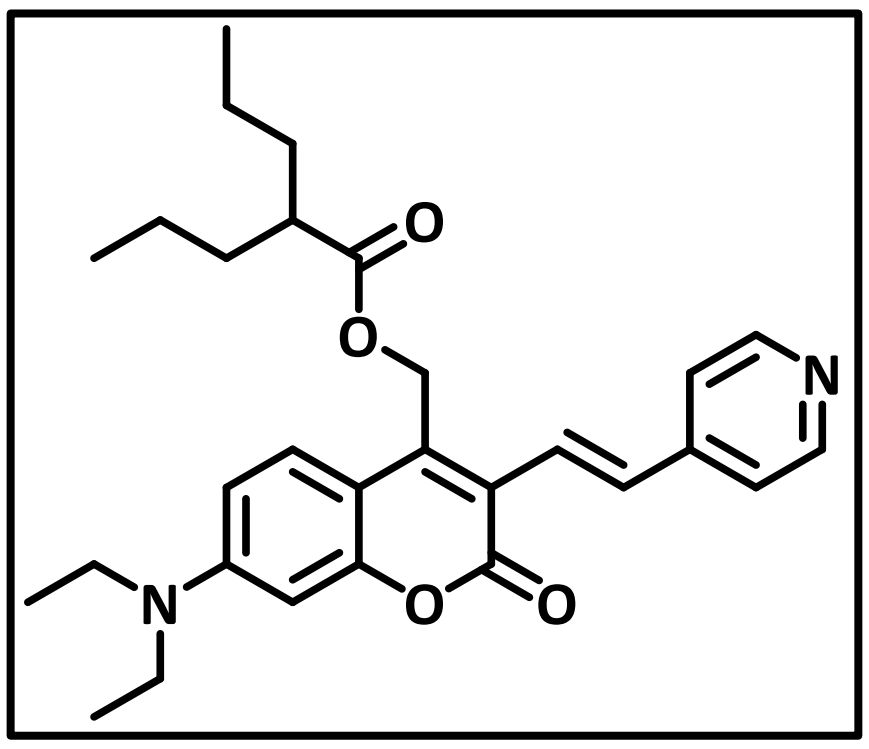

To an 8 ml solution of starting material (**4**) (100 mg, 0.22 mmol) in dry DMF, DIPEA (0.08ml, 0.44 mmol), $Pd(OAc)_2$ (4 mg, 0.02 mmol), tri(*o*-tolyl)phosphine (18 mg, 0.06 mmol), and 4-vinylpyridine (0.07 ml, 0.66 mmol) were added. Then the reaction mixture was purged with Ar-gas. After that, it was refluxed at 110 °C for 2 h. After the completion of the reaction, the organic layer was extracted with EtOAc and washed 2 times with brine solution. Then silica gel column chromatography (EtOAc: Hexane = 1:4) was performed to obtain a solid, red-coloured product. (Yield 41%). **^{1}H NMR** (500 MHz, $CDCl_3$) δ 8.56 (d, *J* = 3.9 Hz, 2H), 7.65 (d, *J* = 16.0 Hz, 1H), 7.53 (d, *J* = 9.2 Hz, 1H), 7.43 (d, *J* = 16.0 Hz, 1H), 7.37 (d, *J* = 5.7 Hz, 2H), 6.63 (dd, *J* = 9.2, 2.5 Hz, 1H), 6.51 (d, *J* = 2.5 Hz, 1H), 5.40 (s, 2H), 3.44 (q, *J* = 7.1 Hz, 4H), 2.46 – 2.39 (m, 1H), 1.64 – 1.54 (m, 2H), 1.46 – 1.36 (m, 2H), 1.29 – 1.20 (m, 10H), 0.82 (t, *J* = 7.3 Hz, 6H). **^{13}C NMR** (126 MHz, $CDCl_3$) δ 176.2, 160.7, 155.6, 151.1, 148.9, 146.9, 144.9, 131.3, 126.6, 126.1, 121.4, 116.5, 109.6, 108.4, 97.5, 57.8, 45.4, 45.0, 34.7, 20.8, 14.0, 12.6. HRMS (ESI) m/z: $[M + H]^+$ calcd for $C_{29}H_{36}N_2O_4$ 476.2675; found 476.2677.

Synthesis of (*E*)-(7-(diethylamino)-3-(4-(dimethylamino)styryl)-2-oxo-2H-chromen-4-yl)methyl 2-propylpentanoate (5b)

To an 8 ml solution of starting material (**4**) (100 mg, 0.22 mmol) in dry DMF, DIPEA (0.08ml, 0.44 mmol), $Pd(OAc)_2$ (4 mg, 0.02 mmol), tri(*o*-tolyl)phosphine (18 mg, 0.06 mmol), and N,N-Dimethyl-4-vinylaniline (0.1 ml, 0.66 mmol) were added. Then the reaction mixture was purged with Ar-gas for 45 min. After that, it was refluxed at 110 °C for 2 h. After the completion of the reaction, the organic layer was extracted with EtOAc and washed 2-3 times with brine solution. Then silica gel column chromatography (EtOAc: Hexane = 3:7) was performed to obtain a solid, red-coloured product. (Yield

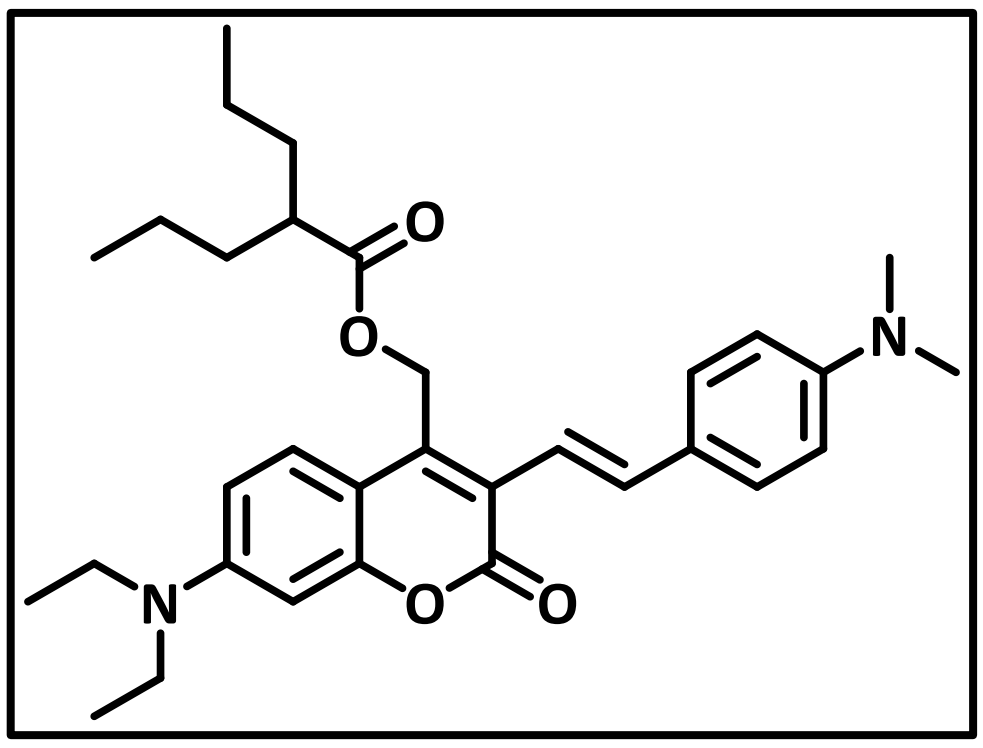

38%). **^{1}H NMR** (500 MHz, $CDCl_3$) δ 7.45 (d, *J* = 10.6 Hz, 1H), 7.41 (dd, *J* = 7.9, 6.3 Hz, 3H), 7.00 (d, *J* = 16.1 Hz, 1H), 6.69 (d, *J* = 8.8 Hz, 2H), 6.60 (dd, *J* = 9.1, 2.5 Hz, 1H), 6.52 (d, *J* = 2.5 Hz, 1H), 5.38 (s, 2H), 3.42 (q, *J* = 7.0 Hz, 4H), 2.98 (s, 6H), 2.48 – 2.41 (m, 1H), 1.66 – 1.60 (m, 2H), 1.47 – 1.40 (m, 2H), 1.34 – 1.27 (m, 4H), 1.21 (t, *J* = 7.1 Hz, 6H), 0.86 (t, *J* = 7.3 Hz, 6H). **^{13}C NMR** (126 MHz, $CDCl_3$) δ 176.4, 161.6, 154.7, 150.5, 149.9, 140.5, 135.7, 128.1, 126.2, 125.8, 120.2, 116.4, 112.4, 109.1, 108.9, 97.7, 58.8, 45.5, 44.9, 40.5, 34.8, 20.9, 14.1, 12.6. HRMS (ESI) m/z: $[M + H]^+$ calcd for $C_{32}H_{42}N_2O_4$ 518.3145; found 518.3141.

Synthesis of (*E*)-(3-(2-(benzo[d]thiazol-2-yl)vinyl)-7-(diethylamino)-2-oxo-2H-chromen-4-yl)methyl 2-propylpentanoate (5c)

To an 8 ml solution of starting material (**4**) (100 mg, 0.22 mmol) in dry DMF, DIPEA (0.08ml, 0.44 mmol), $Pd(OAc)_2$ (4 mg, 0.02 mmol), and tri(*o*-tolyl)phosphine (18 mg, 0.06 mmol) were added, followed by the addition of 2-Vinyl-1,3-benzothiazole (106 mg, 0.66 mmol) by dissolving in DMF. Then the reaction mixture was purged with Ar-gas for 45 min. After that, it was refluxed at 110 °C for 2 h. After the completion of the reaction, the organic layer was extracted with EtOAc and washed twice with brine solution. Then silica gel column chromatography (EtOAc: Hexane = 2:3) was performed to obtain a solid, red-coloured product. (Yield 26%). **^{1}H NMR** (500 MHz, $CDCl_3$) δ 8.13 (d, *J* = 15.7 Hz, 1H), 7.99 (d, *J* = 8.1 Hz, 1H), 7.85 (d, *J* = 7.9 Hz, 1H), 7.76 (d, *J* = 15.7 Hz, 1H), 7.56 (d, *J* = 9.2 Hz, 1H), 7.45 (t, *J* = 7.4 Hz, 1H), 7.36 (d, *J* = 7.7 Hz, 1H), 6.63 (dd, *J* = 9.2, 2.3 Hz, 1H), 6.51 (d, *J* = 2.3 Hz, 1H), 5.45 (s, 2H), 3.44 (q, *J* = 7.1 Hz, 4H), 2.47-2.41 (m, 1H), 1.64 – 1.58 (m, 2H), 1.42 (td, *J* = 7.4, 2.3 Hz, 2H), 1.31 – 1.25 (m, 4H), 1.23 (t, *J* = 7.1 Hz, 6H), 0.82 (t, *J* = 7.3 Hz, 6H). **^{13}C NMR** (126 MHz, $CDCl_3$) δ 176.2, 167.7, 160.4, 155.7, 154.3, 151.2, 145.5, 134.9, 128.9, 127.1, 126.7, 126.4, 125.3, 123.2, 121.6, 116.0, 109.6, 108.4, 97.5, 57.7, 45.4, 45.1, 34.7, 20.8, 14.1, 12.6. HRMS (ESI) m/z: $[M + H]^+$ calcd for $C_{31}H_{36}N_2O_4S$ 532.2396; found 532.2395.

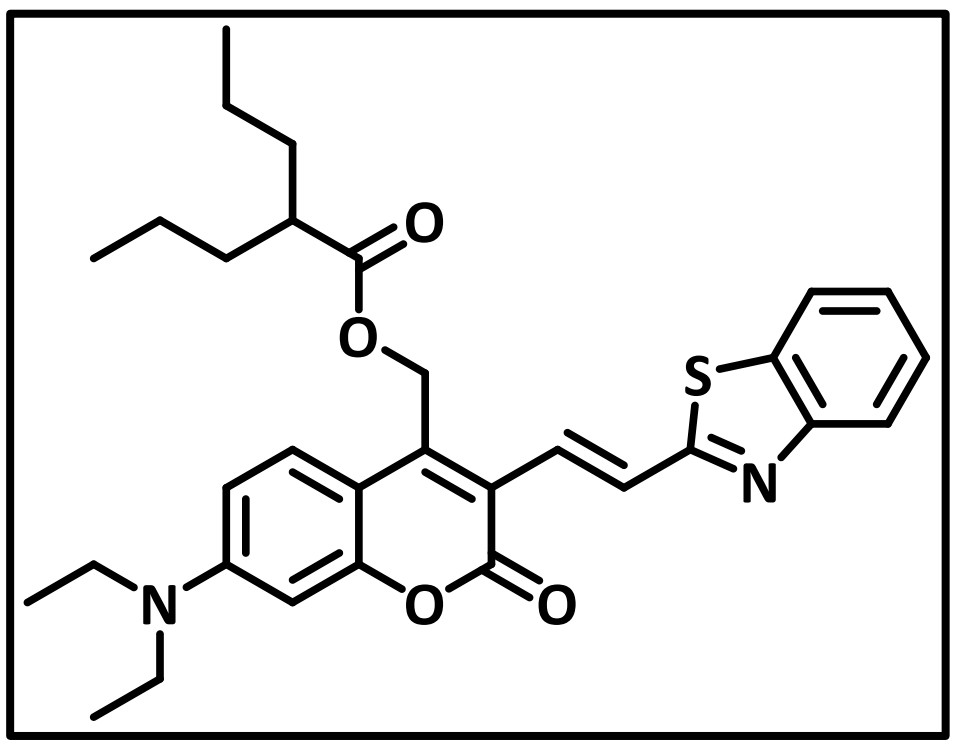

Synthesis of (*E*)-2-(2-(7-(diethylamino)-2-oxo-4-(((2-propylpentanoyl)oxy)methyl)-2H-chromen-3-yl)vinyl)-3-methylbenzo[*d*]thiazol-3-ium (5d)

To the 10 ml solution of PRPG (**5c**) (100 mg, 0.19 mmol) in dry DCM, Meerwein's salt (83 mg, 0.56 mmol) was added, and the reaction mixture was left at room temperature for 2 h. Then silica gel column chromatography (Methanol: DCM = 1:19) was performed to obtain a solid, violet-coloured product. (Yield 21%). **^{1}H NMR** (500 MHz, $CDCl_3$) δ 8.45 (d, *J* = 14.9 Hz, 1H), 8.35 (d, *J* = 14.9 Hz, 1H), 8.07 – 8.00 (m, 2H), 7.84 – 7.76 (m, 2H), 7.66 (t, *J* = 7.7 Hz, 1H), 6.76 – 6.71 (m, 1H), 6.47 (d, *J* = 1.8 Hz, 1H), 5.58 (s, 2H), 4.27 (s, 3H), 3.50 (q, *J* = 7.0 Hz, 4H), 2.43-2.38 (m, 1H), 1.54 (dd, *J* = 13.6, 7.9 Hz, 2H), 1.41 – 1.37 (m, 2H), 1.28 – 1.25 (m, 6H), 1.17 (d, *J* = 7.4 Hz, 4H), 0.78 (t, *J* = 7.3 Hz, 6H). **^{13}C NMR** (126 MHz, $CDCl_3$) δ 176.1, 172.8, 159.9, 157.1, 153.4, 152.4, 142.7, 142.1, 130.2, 129.1, 128.8, 127.8, 123.5, 116.5, 113.7, 111.9, 111.0, 109.0, 97.2, 77.4, 57.3, 45.5, 45.2, 36.2, 34.5, 20.7, 14.1, 12.7. HRMS (ESI) m/z: $[M]^+$ calcd for $C_{32}H_{39}N_2O_4S^+$ 547.2631; found 547.2632.

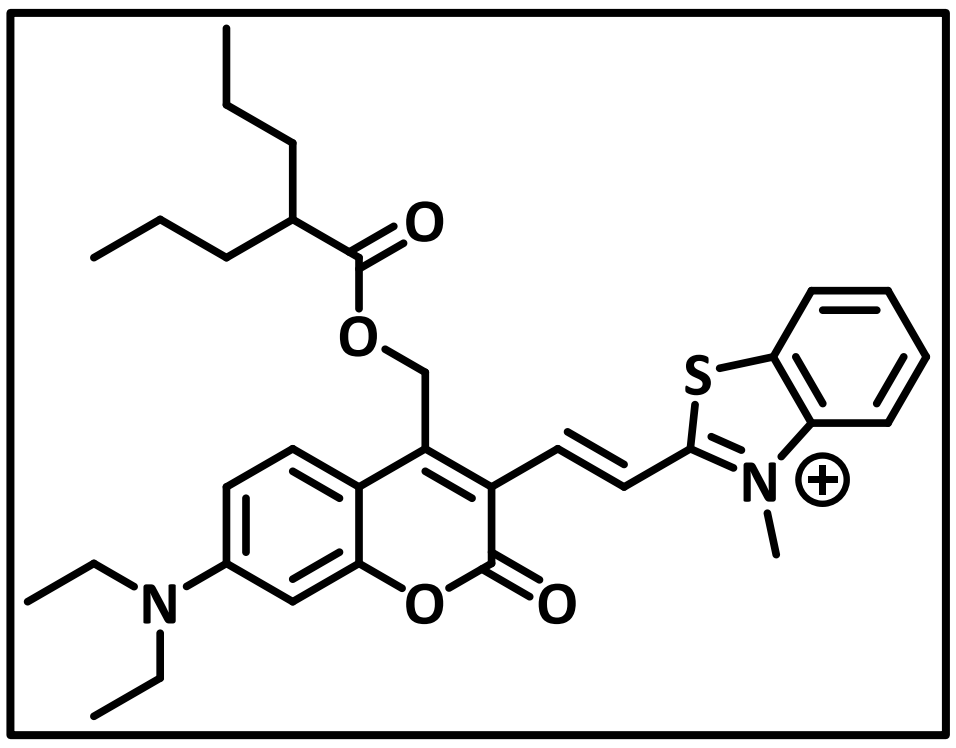

3. ^{1}H and ^{13}C NMR spectra of compounds 2-4 and coumarin fluorogen-based PRPGs 5a-d:

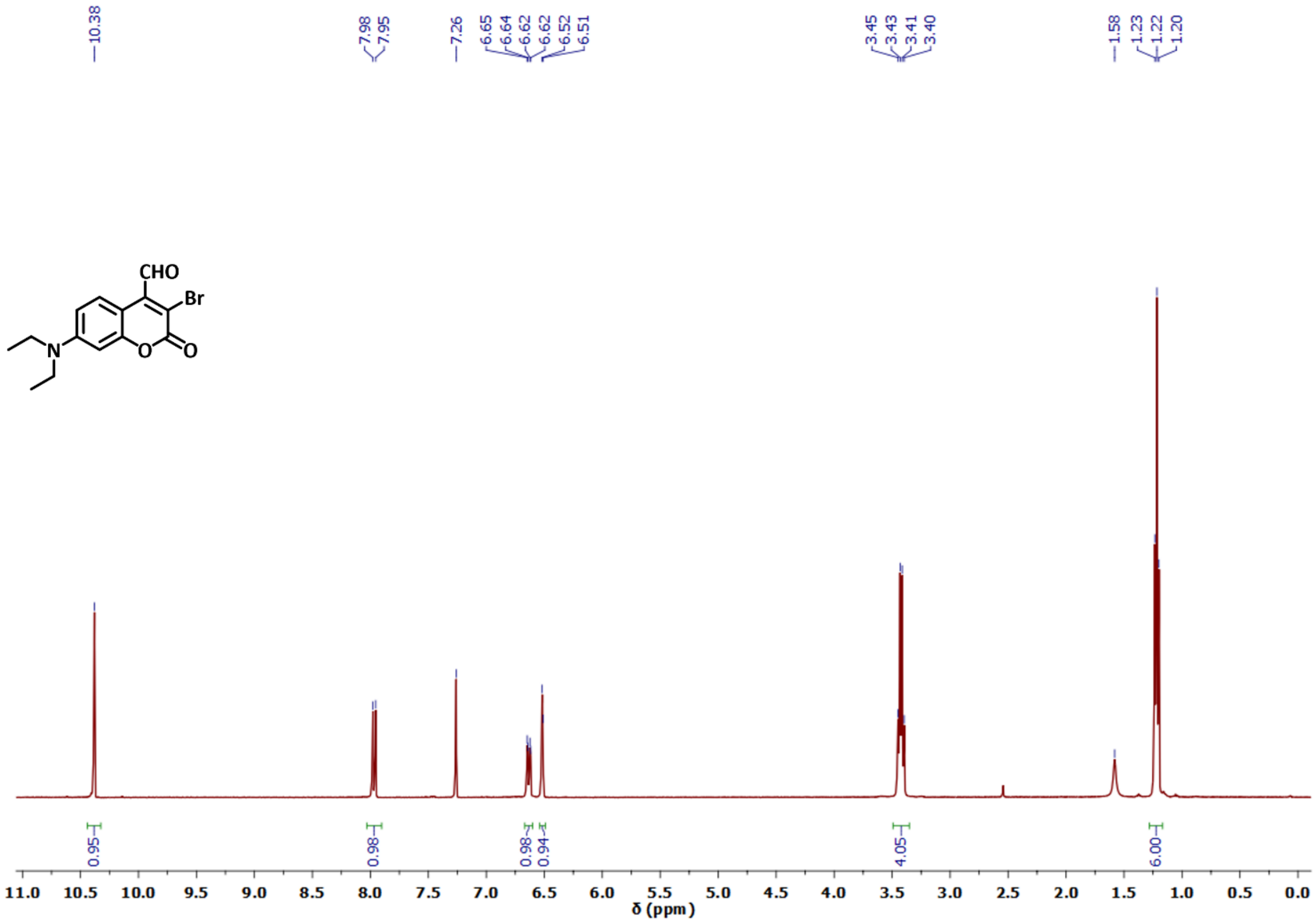

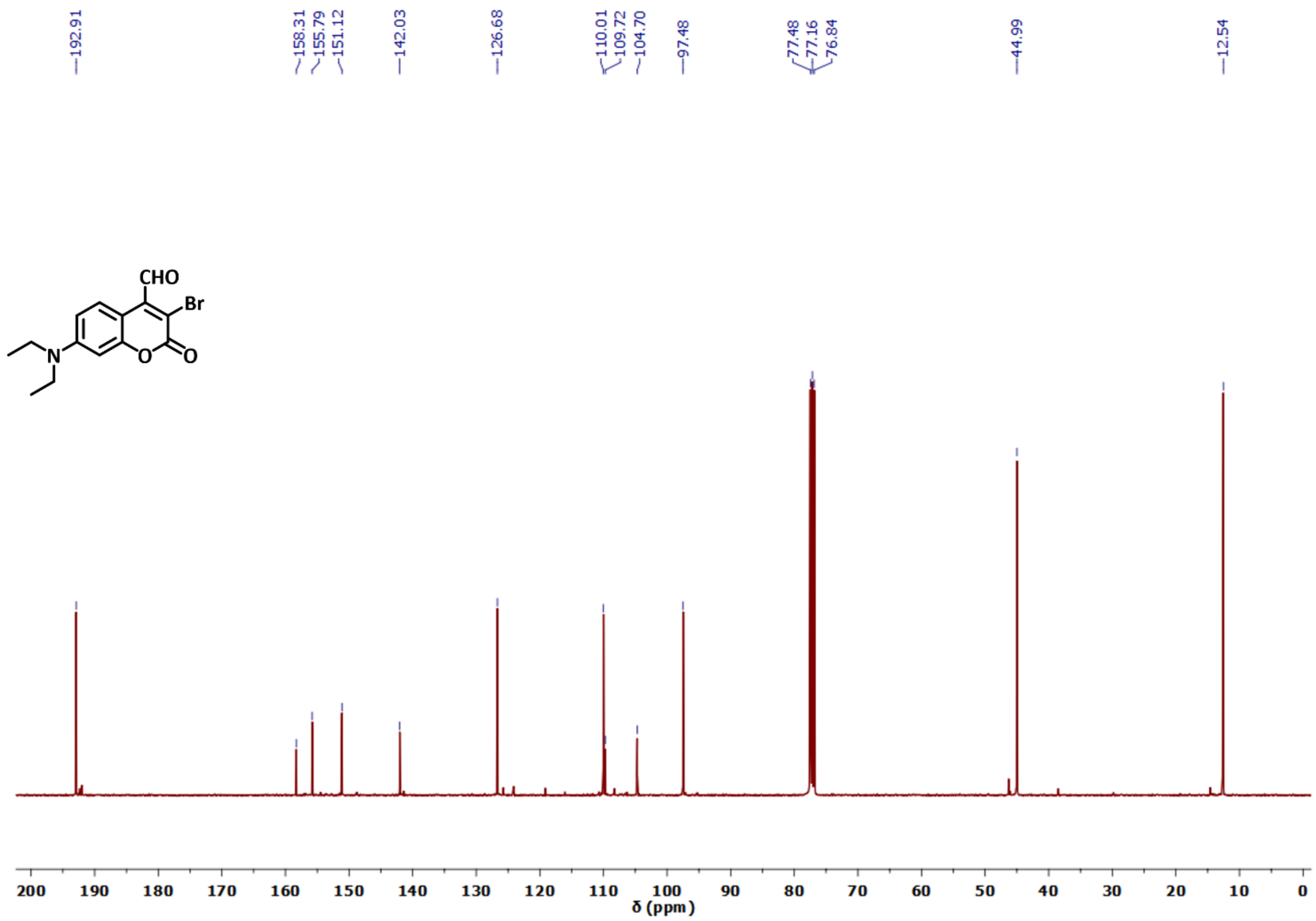

Figure S1 ^{1}H (400 MHz) and ^{13}C (101 MHz) NMR spectra of compound **2** in $CDCl_3$.

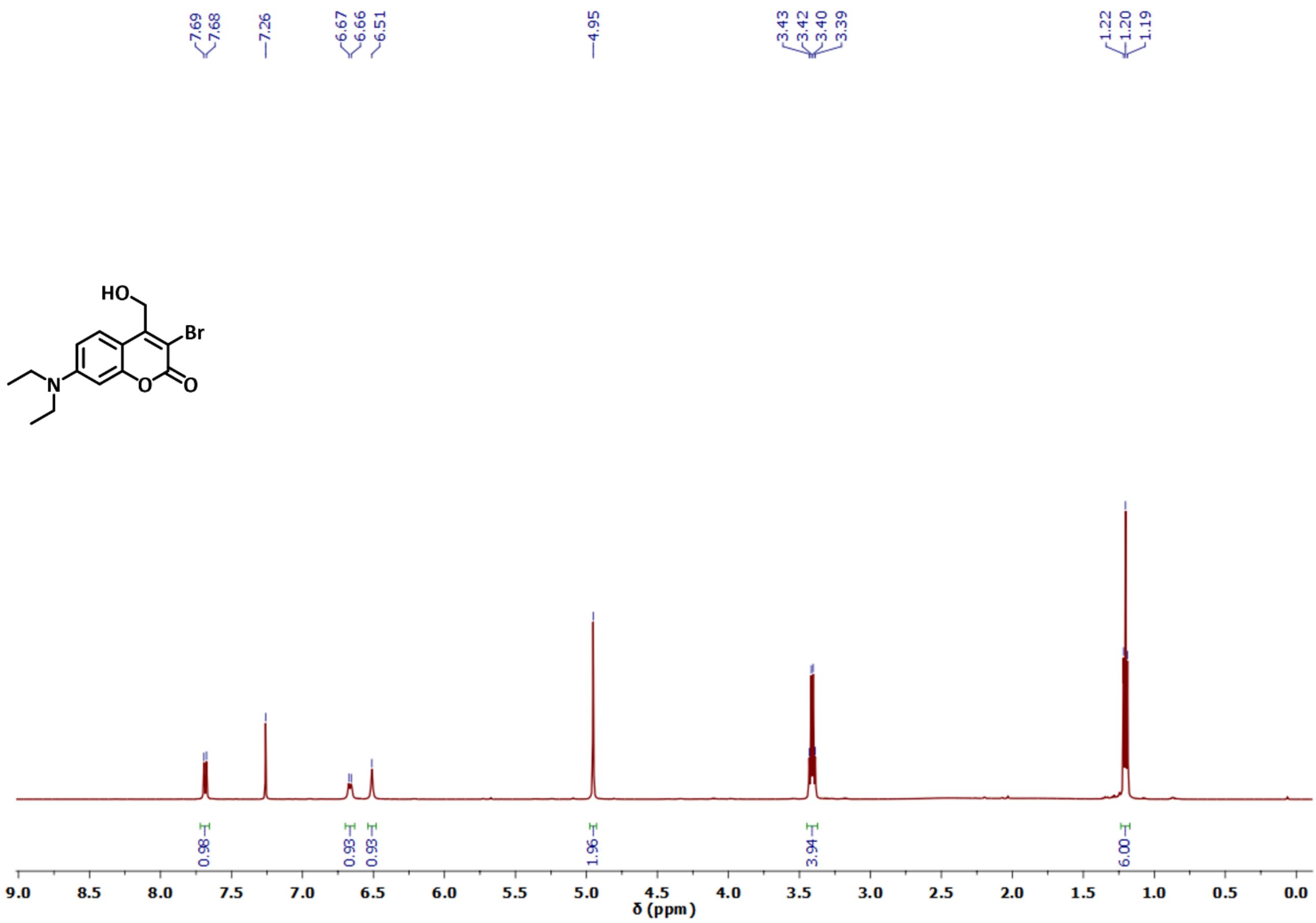

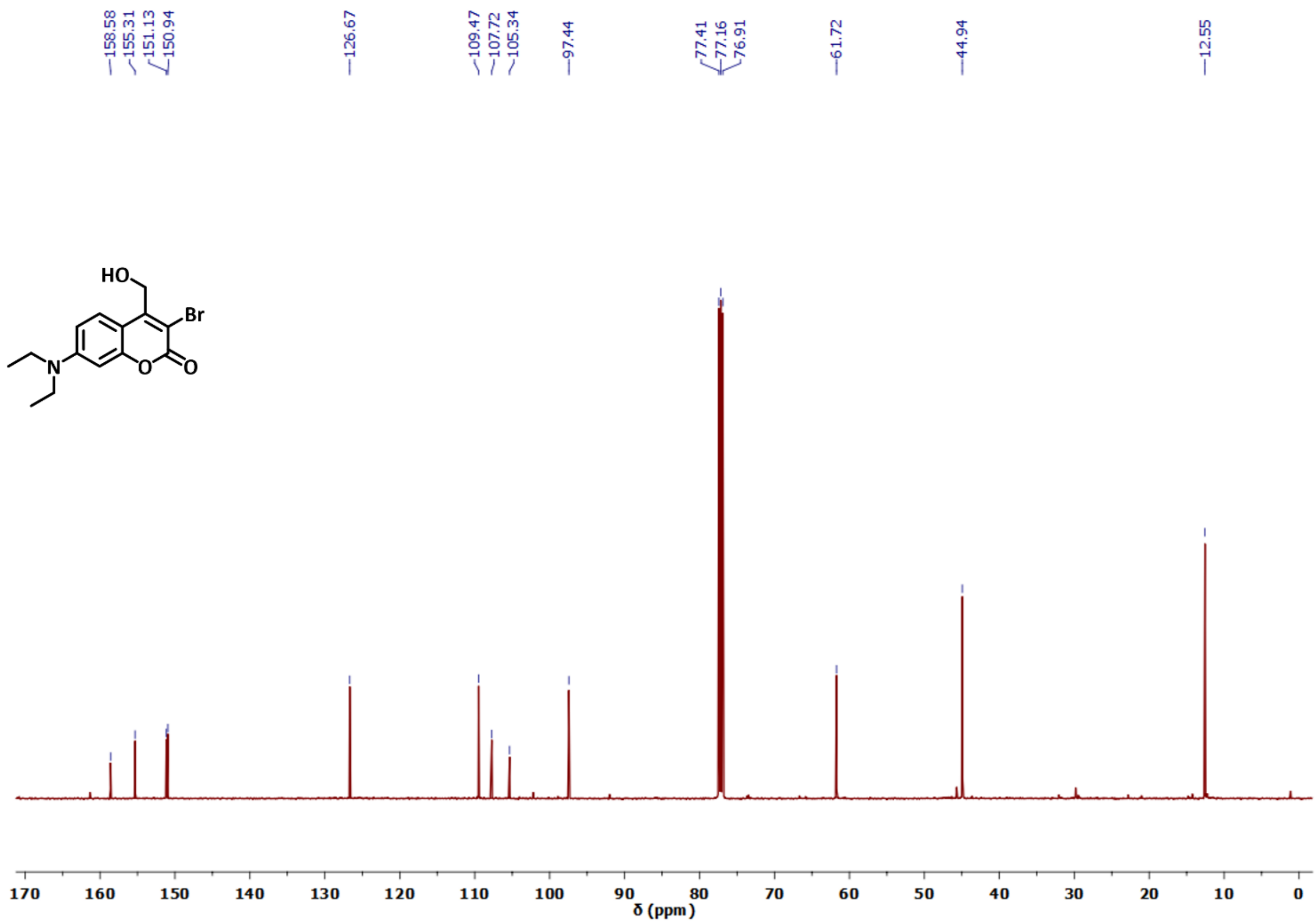

Figure S2 ^{1}H (500 MHz) and ^{13}C (126 MHz) NMR spectra of compound **3** in $CDCl_3$.

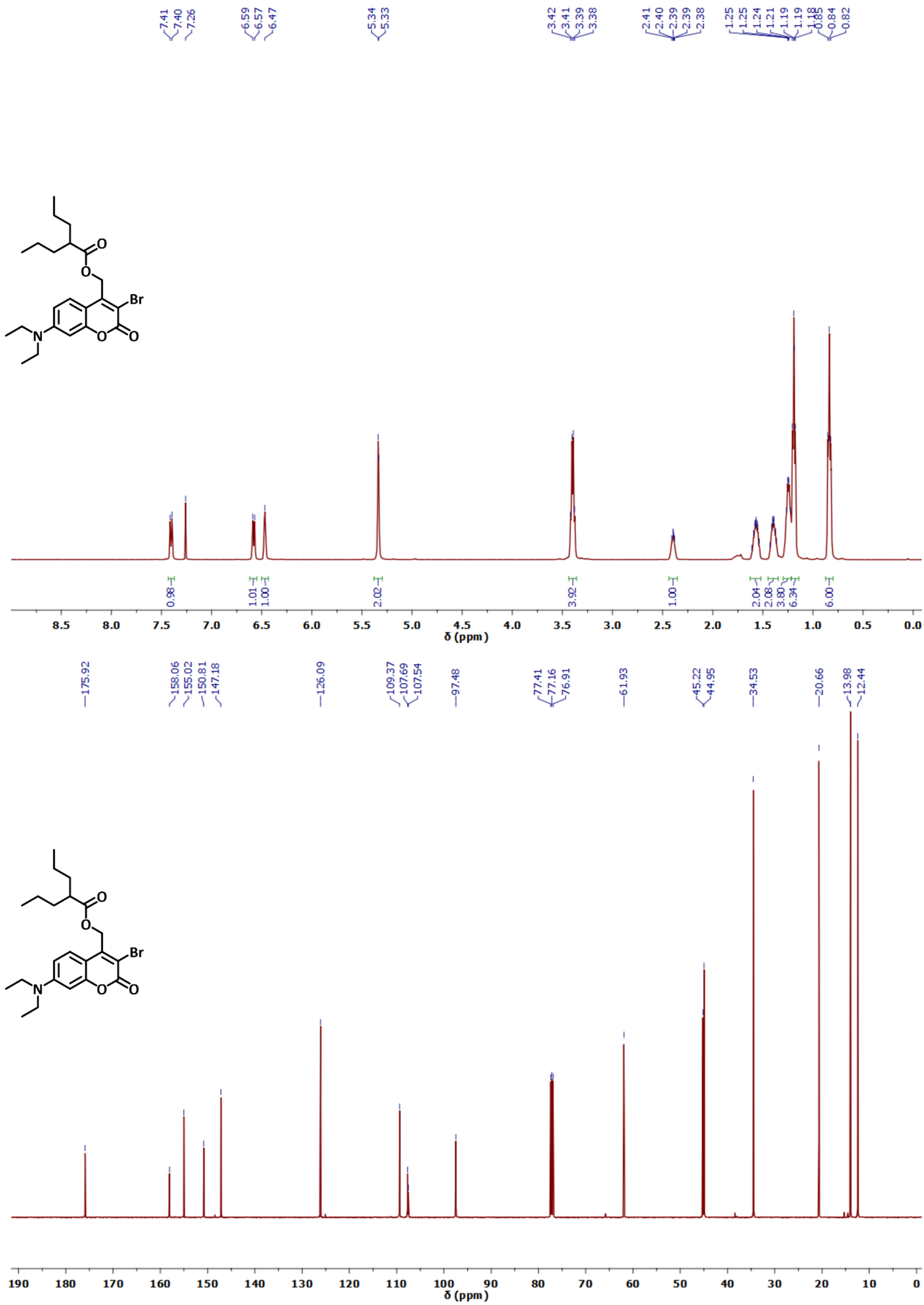

Figure S3 ^{1}H (500 MHz) and ^{13}C (126 MHz) NMR spectra of compound **4** in $CDCl_3$.

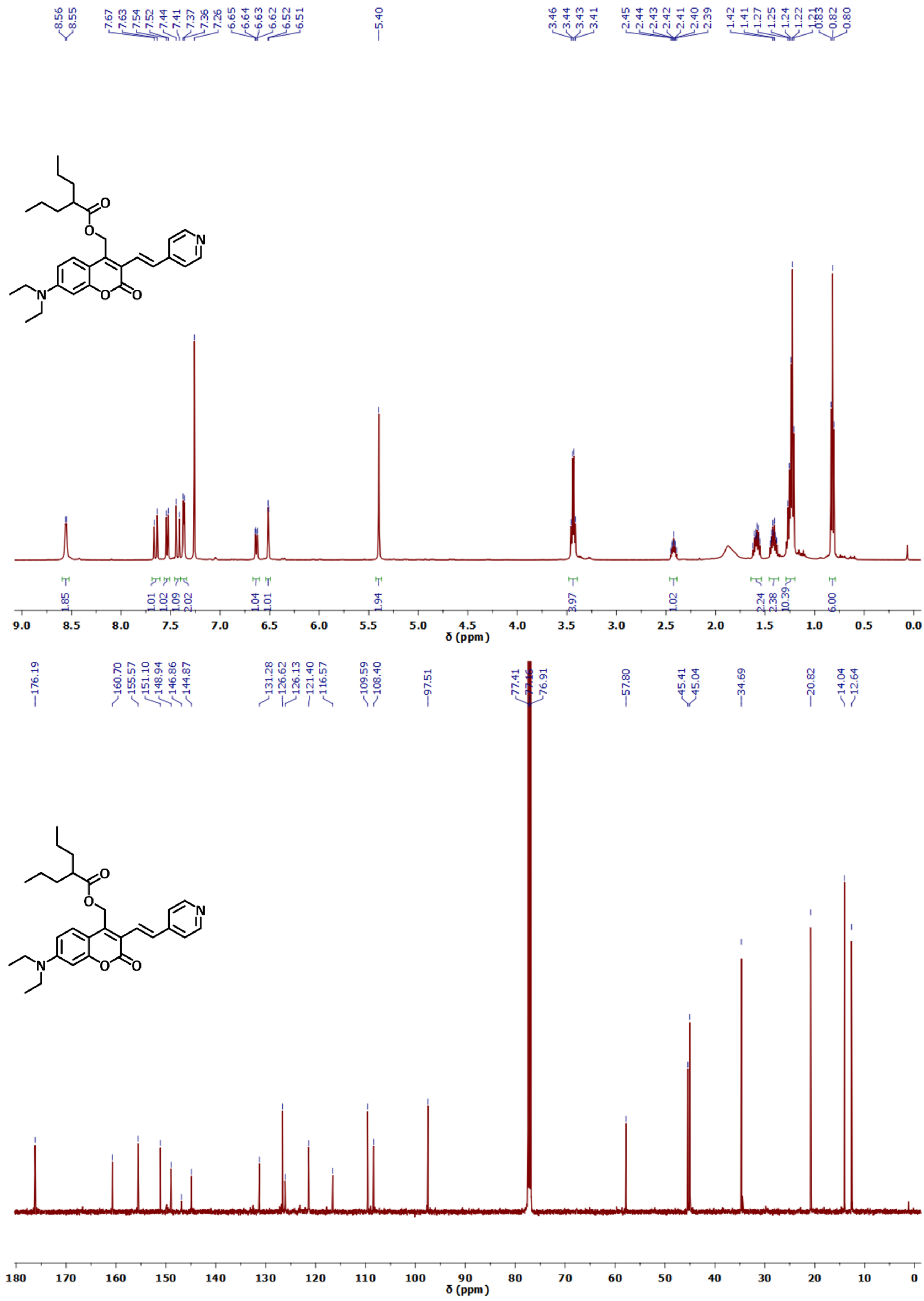

Figure S4 ^{1}H (500 MHz) and ^{13}C (126 MHz) NMR spectra of coumarin fluorogen-based PRPG **5a** in $CDCl_3$.

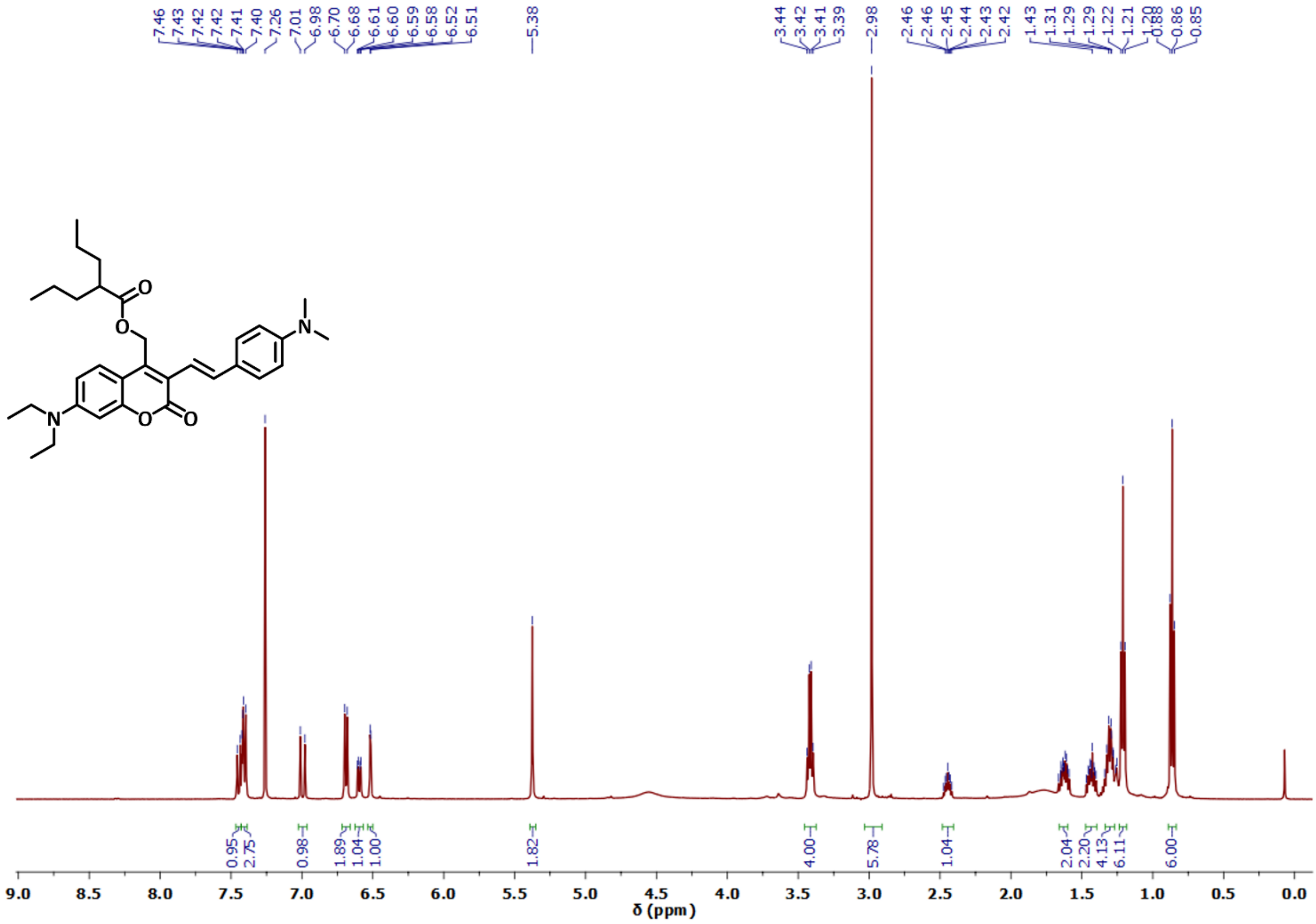

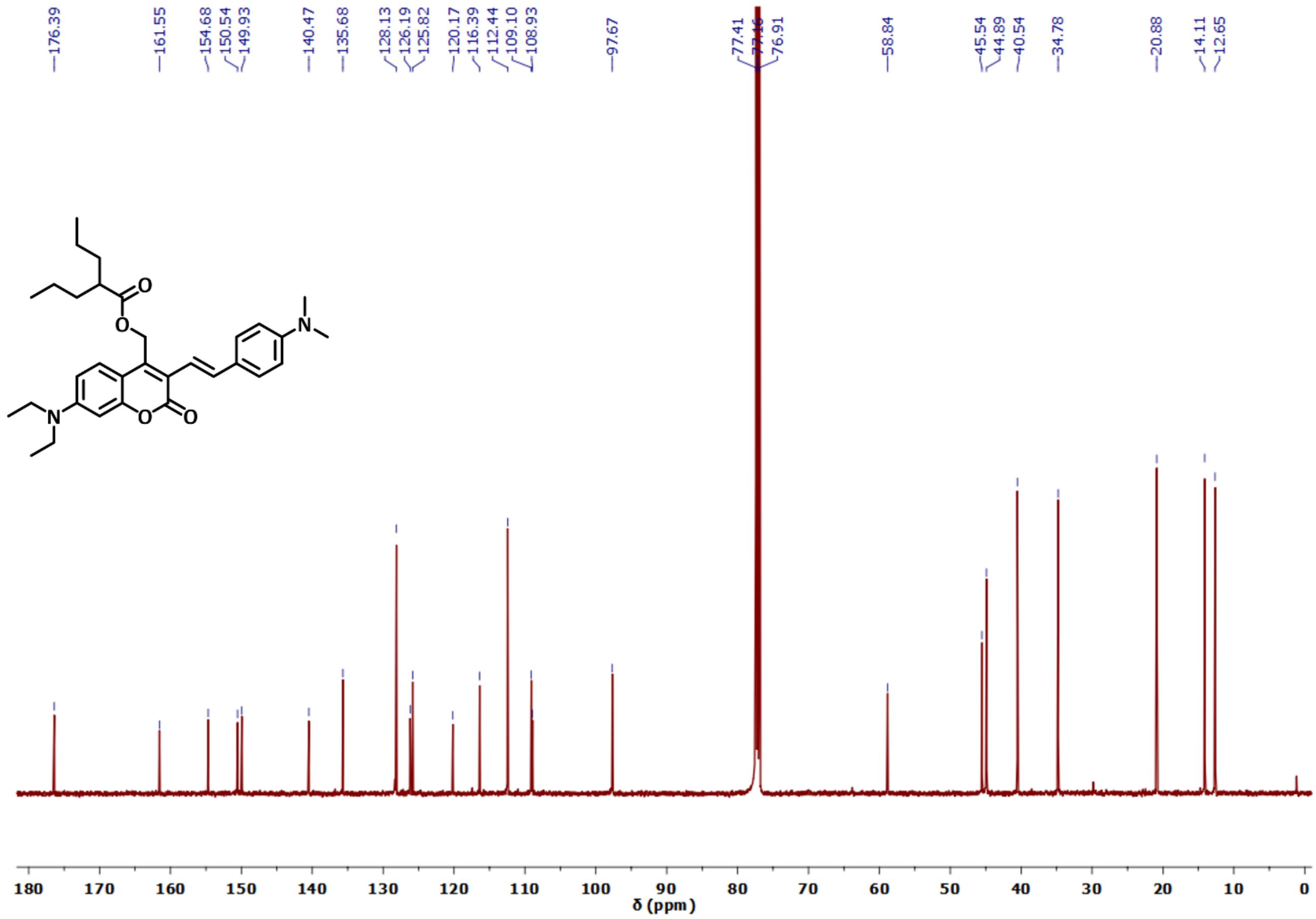

Figure S5 ^{1}H (500 MHz) and ^{13}C (126 MHz) NMR spectra of coumarin fluorogen-based PRPG **5b** in $CDCl_3$.

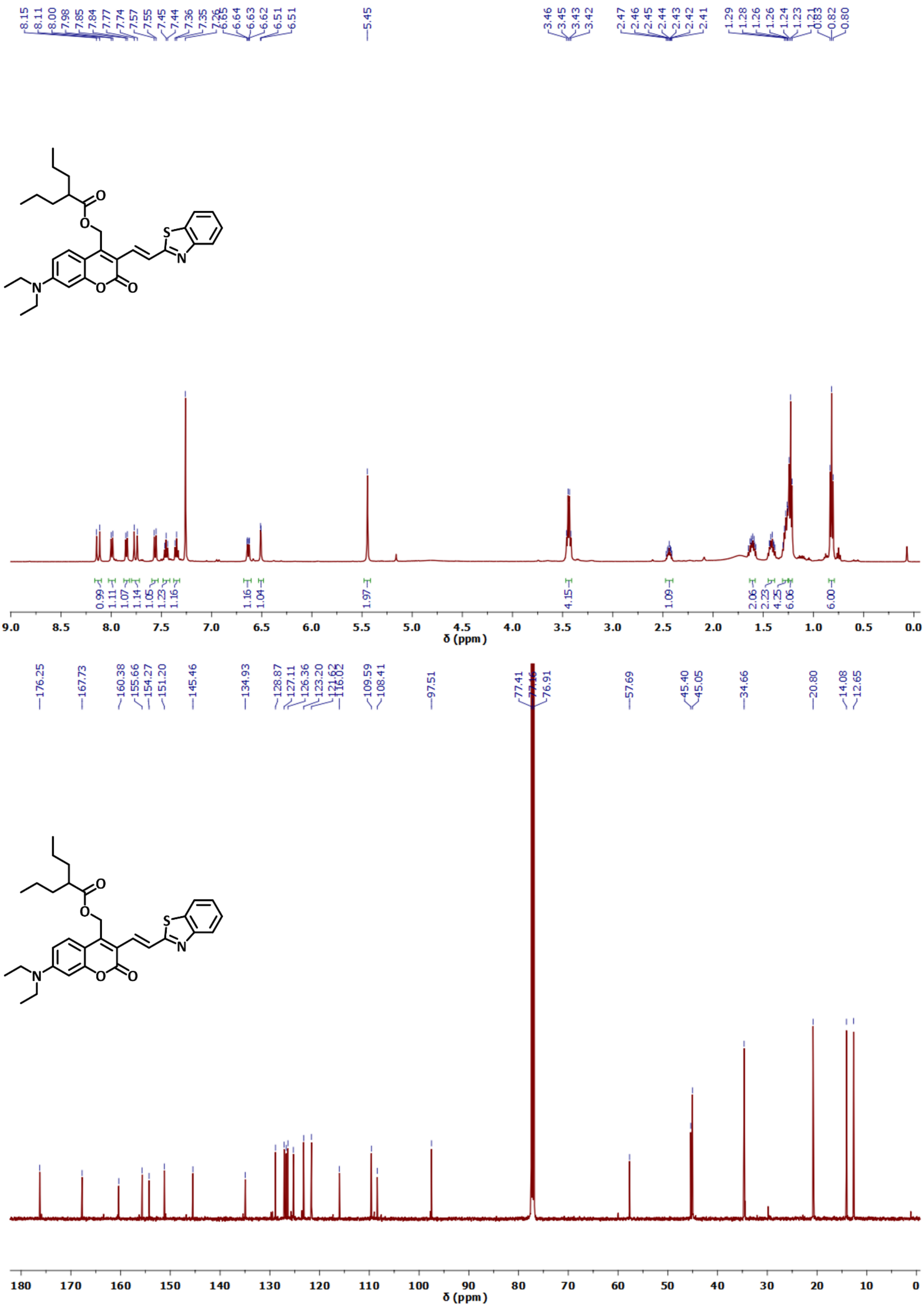

Figure S6 ^{1}H (500 MHz) and ^{13}C (126 MHz) NMR spectra of coumarin fluorogen-based PRPG **5c** in $CDCl_3$.

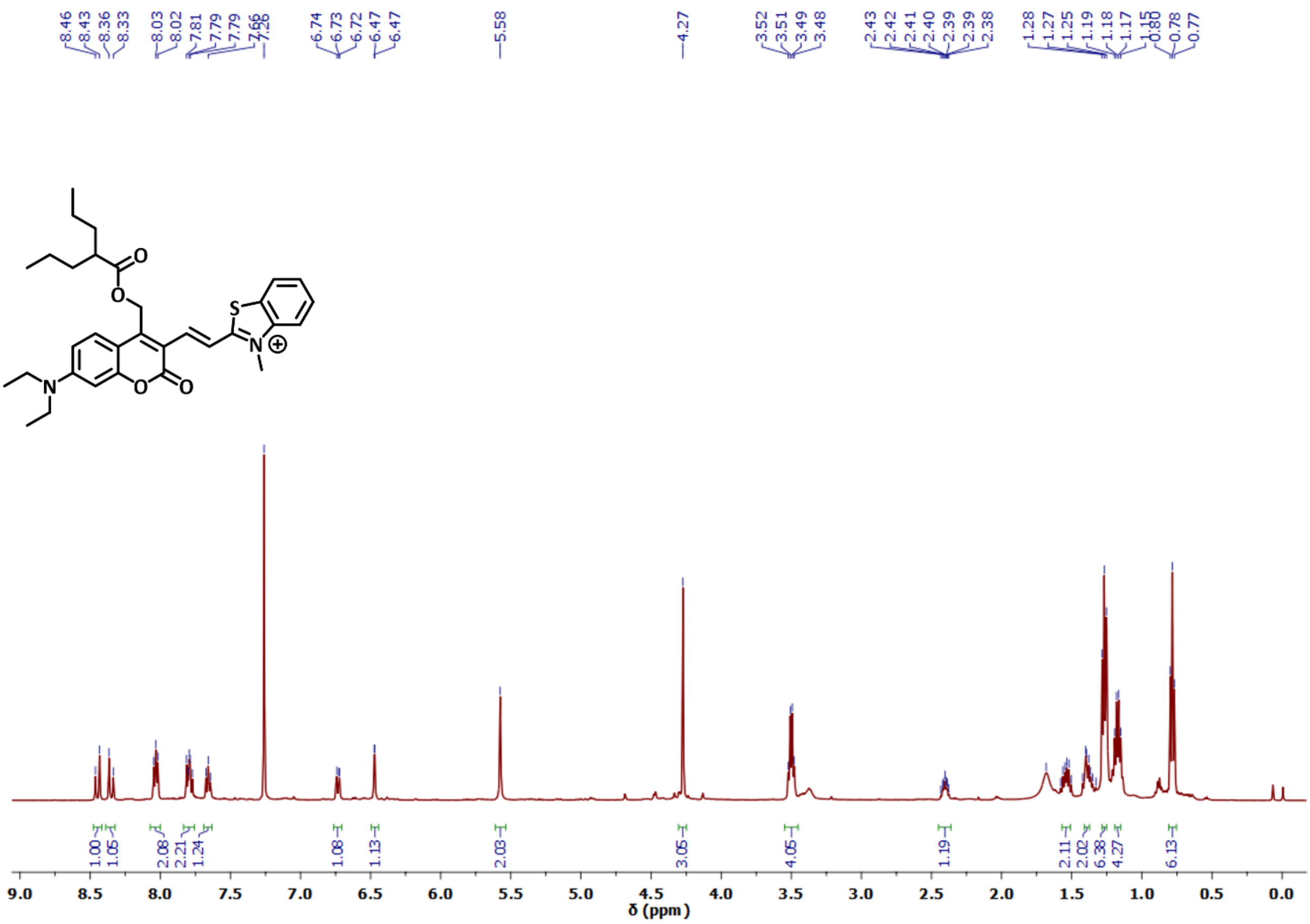

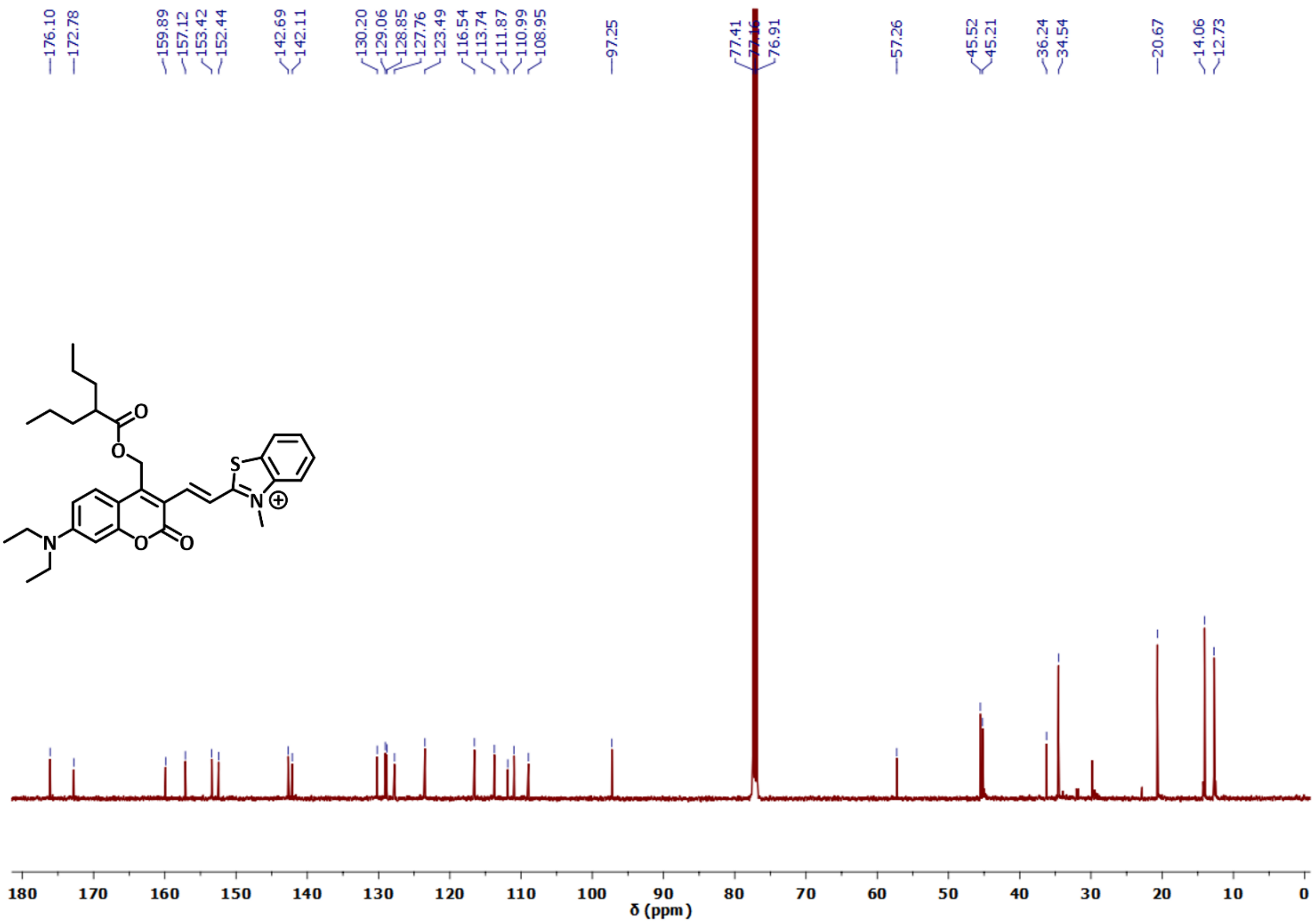

Figure S7 ^{1}H (500 MHz) and ^{13}C (126 MHz) NMR spectra of coumarin fluorogen-based PRPG **5d** in $CDCl_3$.

4. HRMS of compounds 2-4 and fluorogen-based PRPGs 5a-d:

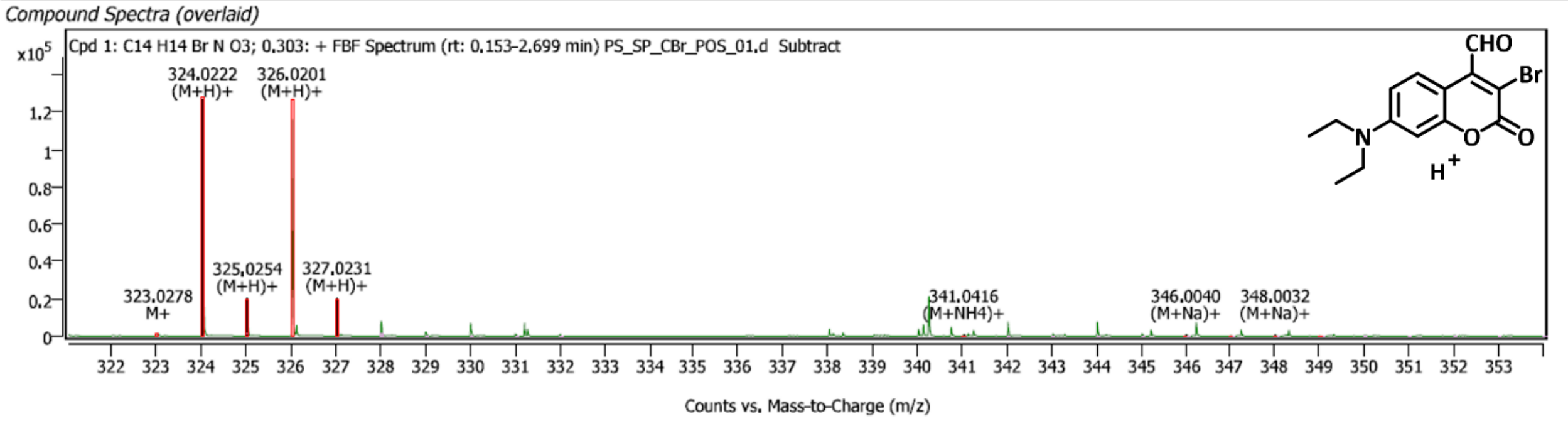

Compound ID Table

Name	Formula	Species	RT	RT Diff	Mass	CAS	ID Source	Score	Score (Lib)	Score (Tgt)
	C14 H14 Br N O3	M+ (M+H)+ (M+NH4)+ (M+Na)+	0,303		323,0148		FBF	96,94		96,94

Figure S8 HRMS of compound **2**.

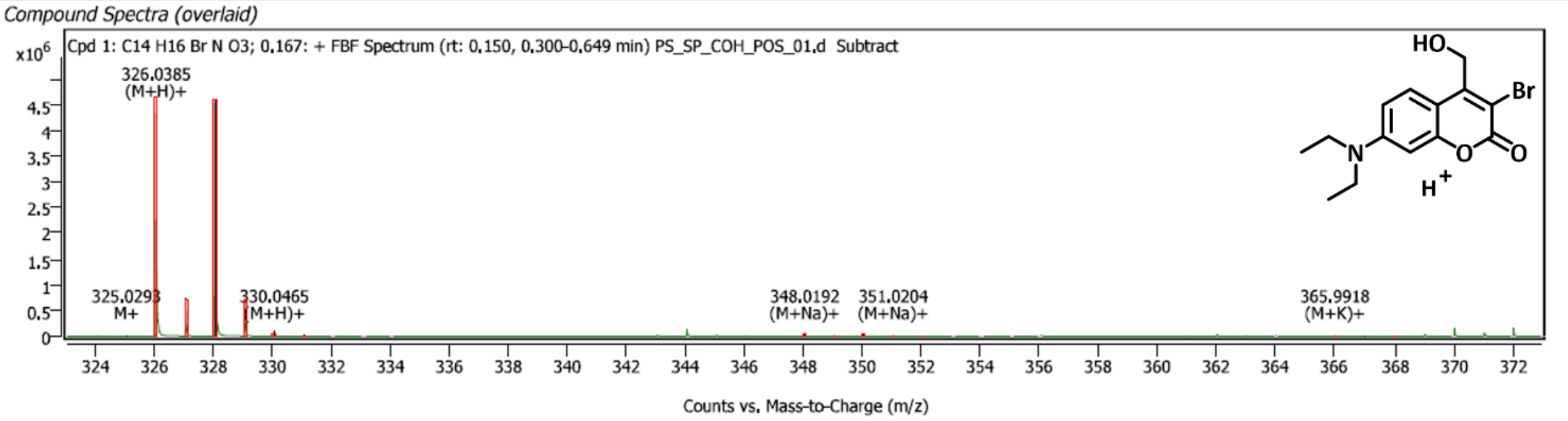

Compound ID Table

Name	Formula	Species	RT	RT Diff	Mass	CAS	ID Source	Score	Score (Lib)	Score (Tgt)
	C14 H16 Br N O3	M+ (M+H)+ (M+Na)+ (M+K)+	0,167		325,0312		FBF	99,56		99,56

Figure S9 HRMS of compound **3**.

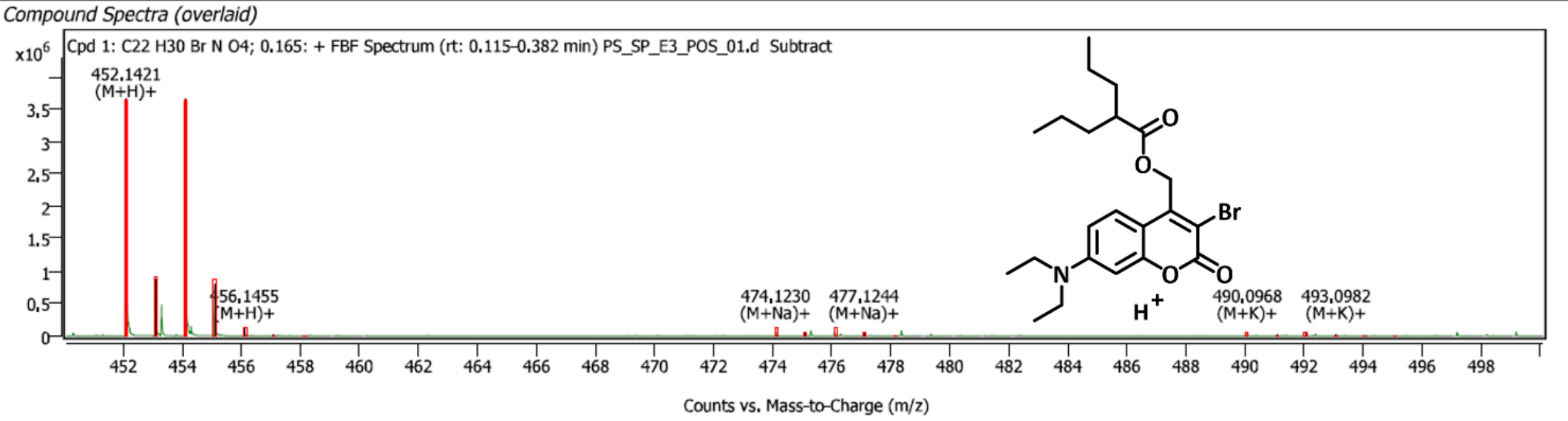

Compound ID Table

Name	Formula	Species	RT	RT Diff	Mass	CAS	ID Source	Score	Score (Lib)	Score (Tgt)
	C22 H30 Br N O4	(M+H)+ (M+Na)+ (M+K)+	0,165		451,1347		FBF	96,53		96,53

Figure S10 HRMS of compound **4**.

Compound Spectra (overlaid)

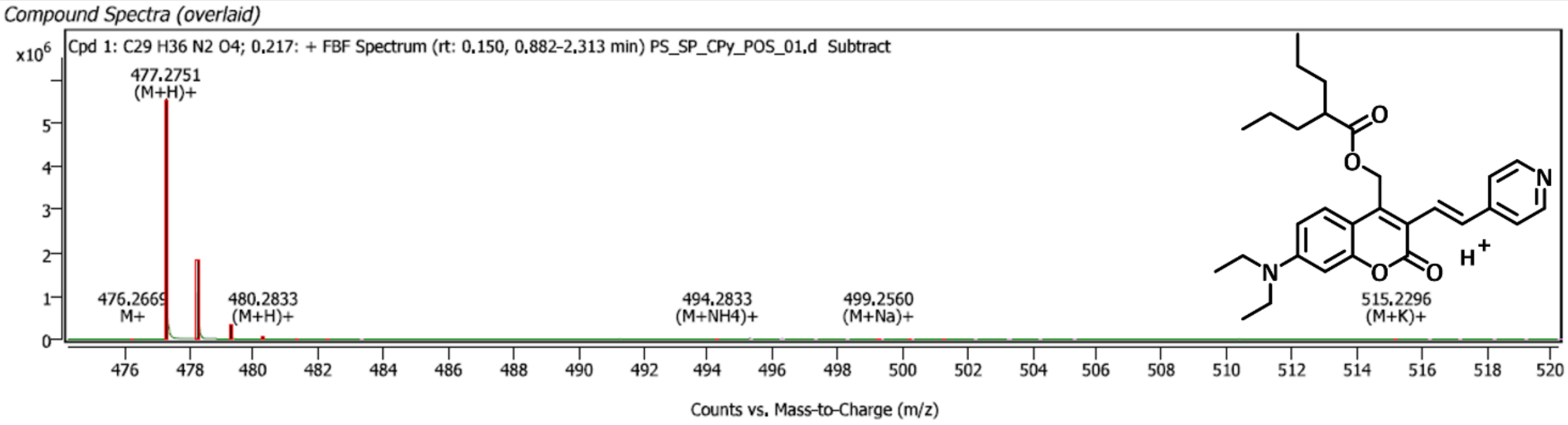

Compound ID Table

Name	Formula	Species	RT	RT Diff	Mass	CAS	ID Source	Score	Score (Lib)	Score (Tgt)
	C29 H36 N2 O4	M+ (M+H)+ (M+NH4)+ (M+Na)+ (M+K)+	0,217		476,2677		FBF	99,53		99,53

Figure S11 HRMS of coumarin fluorogen-based PRPG **5a**.

Compound Spectra (overlaid)

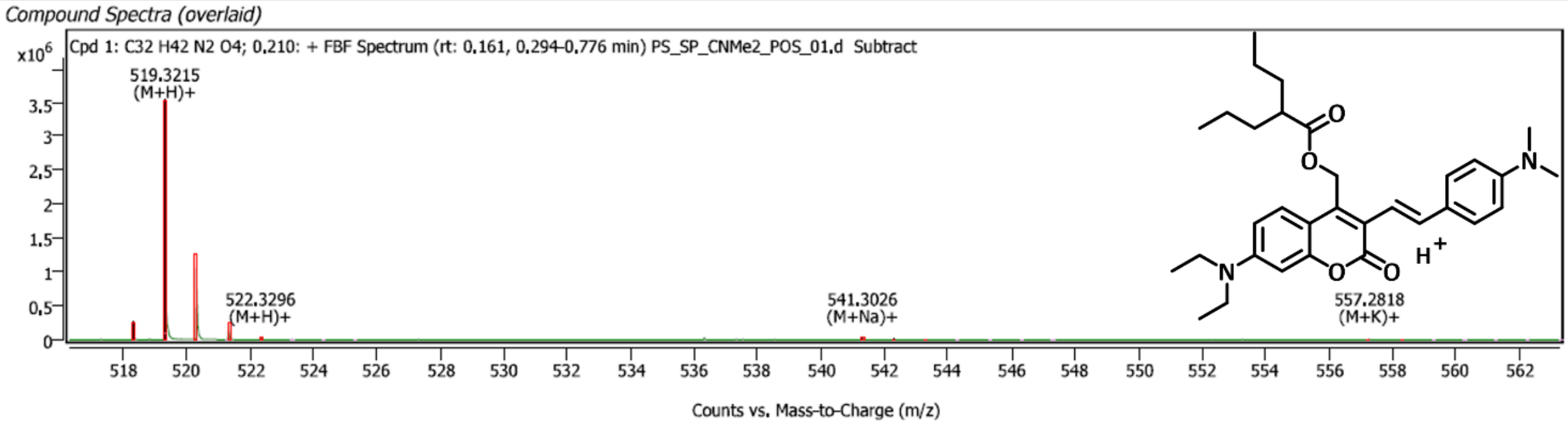

Compound ID Table

Name	Formula	Species	RT	RT Diff	Mass	CAS	ID Source	Score	Score (Lib)	Score (Tgt)
	C32 H42 N2 O4	M+ (M+H)+ (M+Na)+ (M+K)+	0,210		518,3141		FBF	98,40		98,40

Figure S12 HRMS of coumarin fluorogen-based PRPG **5b**.

Compound Spectra (overlaid)

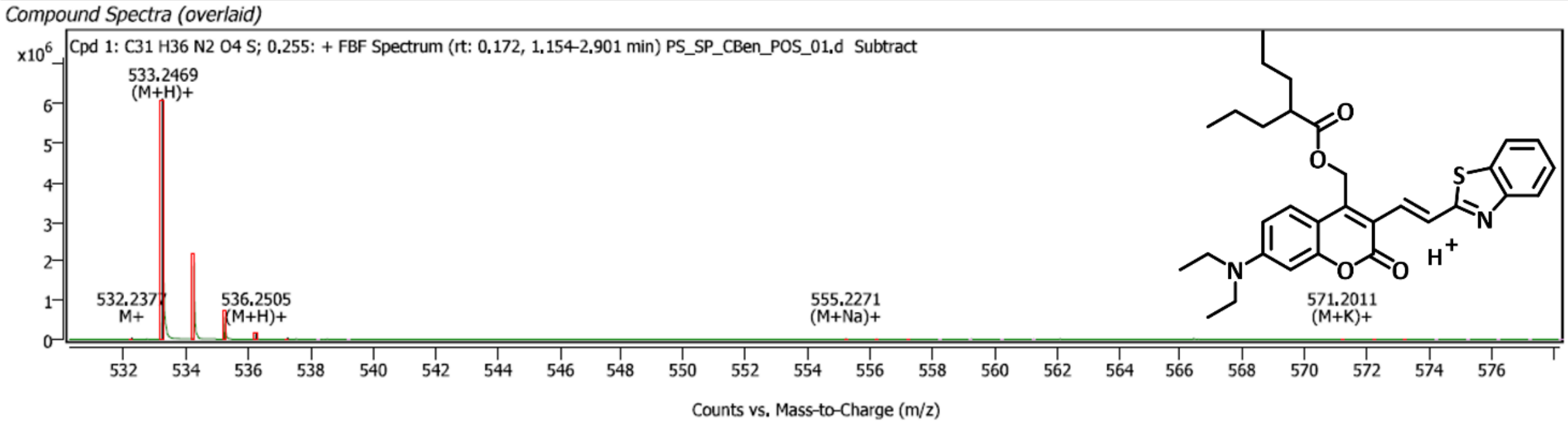

Compound ID Table

Name	Formula	Species	RT	RT Diff	Mass	CAS	ID Source	Score	Score (Lib)	Score (Tgt)
	C31 H36 N2 O4 S	M+ (M+H)+ (M+Na)+ (M+K)+	0,255		532,2395		FBF	98,16		98,16

Figure S13 HRMS of coumarin fluorogen-based PRPG **5c**.

Compound Spectra (overlaid)

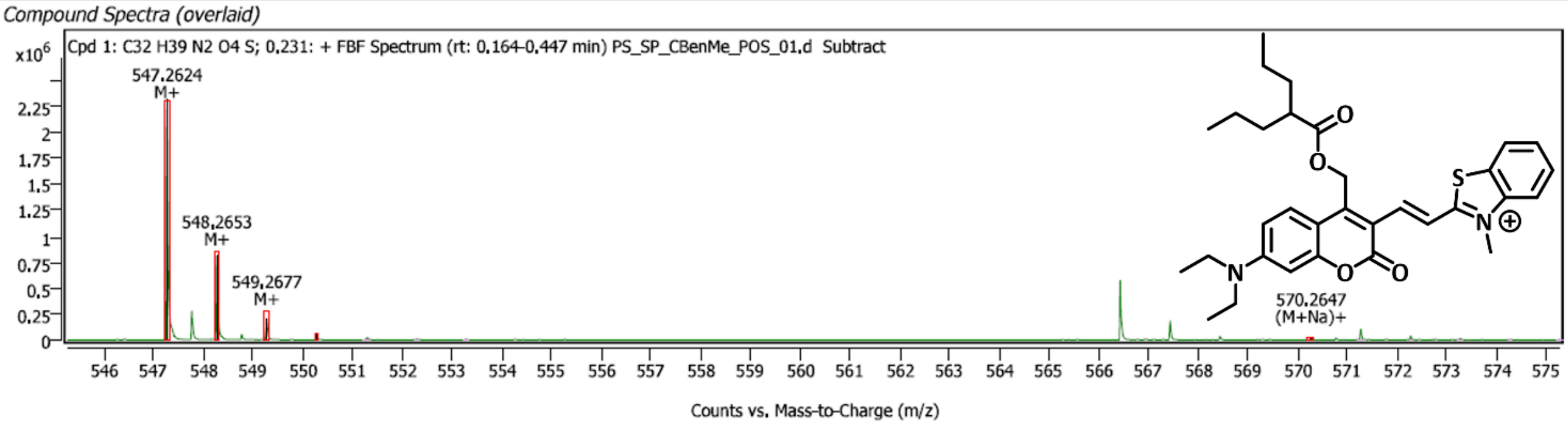

Compound ID Table

Name	Formula	Species	RT	RT Diff	Mass	CAS	ID Source	Score	Score (Lib)	Score (Tgt)
	C32 H39 N2 O4 S	M+ (M+Na)+	0,231		547,2632		FBF	96,05		96,05

Figure S14 HRMS of coumarin fluorogen-based PRPG **5d**.

5. General photophysical characterization methods:

Absorbance measurements. Coumarin fluorogen-based PRPGs **5a-d** were dissolved in DCM to make 1 mM stock solutions. The prepared stock solutions were individually diluted to 0.01 mM solutions using acetonitrile (ACN) and a 5:95 (v/v) ACN/water mixture. The absorbance spectra of all the derivatives were measured using the Shimadzu UV-1900i UV/vis spectrophotometer.
Fluorescence measurements. 1mM stock solutions of all the coumarin fluorogen-based PRPGs (**5a-d**) were prepared in DCM and diluted with ACN, solvents of different polarities (e.g. Toluene, THF, DMSO, Methanol), different ACN-water fractions (like 0%, 20%, 40%, 60%, 80%, 90% water), glycerol-methanol mixture of different percentages, and PBS buffer of pH 7.4. All the emission spectra were recorded using the Shimadzu RF6000 fluorescence spectrophotometer. Also, the temperature-dependent emission in glycerol-methanol mixture was recorded using a Hitachi F7000 fluorescence spectrophotometer.
Time-correlated single photon counting measurements. Stock solutions of 1mM for the coumarin fluorogen-based PRPGs **5a-d** were prepared by dissolving them in DCM. The stock solutions of all PRPGs were individually diluted to 0.001 mM solutions using methanol and a methanol-glycerol (1:9 v/v) mixture. Time-correlated single photon counting (TCSPC) measurements were performed using the HORIBA Deltaflex TCSPC lifetime fluorometer in the presence and absence of human TDP-43 synthetic peptide for all solutions.

Photophysical properties of coumarin fluorogen-based PRPGs 5a-d

1 mM stock solutions of coumarin fluorogen-based PRPGs **5a-d** in DCM were individually diluted to a 4 mL, 0.01 mM solution using ACN and an ACN-Water (5:95) mixture. The absorption and emission spectra of all the coumarin fluorogen-based PRPGs were recorded on a UV-Vis spectrophotometer and a fluorescence spectrophotometer, respectively.

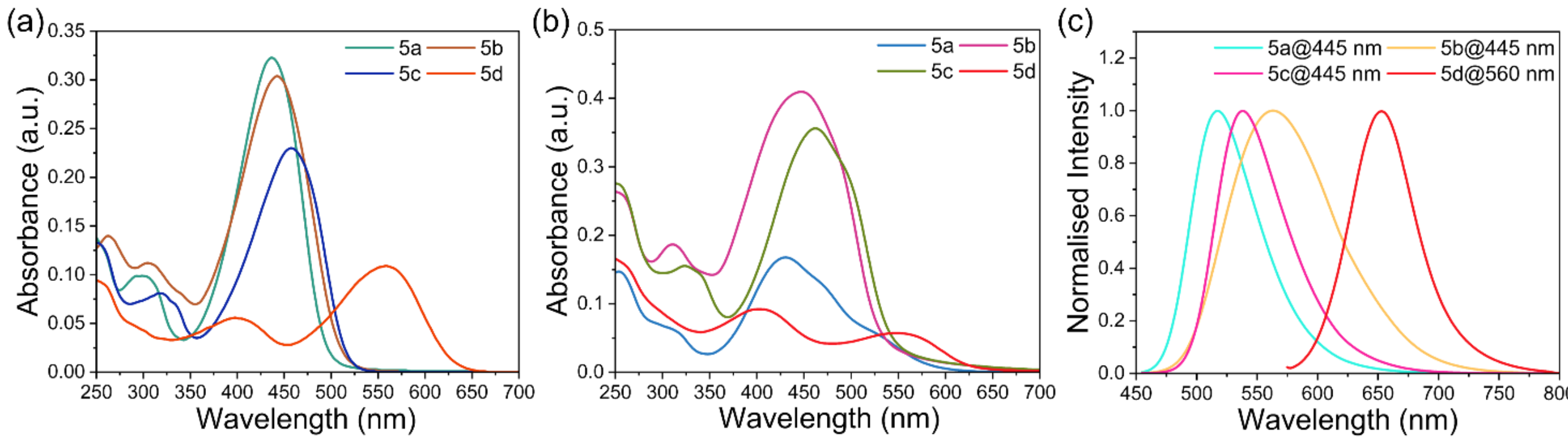

Figure S15 Absorption spectra of coumarin fluorogen-based PRPGs **5a-d** in (a) ACN, (b) ACN/Water (5:95), and (c) emission spectra of **5a-d** in ACN. The final concentration of the coumarin fluorogen-based PRPGs was 0.01 mM. The emission spectra were recorded at wavelengths near the maximum absorption.

Measurement of fluorescence quantum yield

The fluorescence quantum yield of coumarin fluorogen-based PRPGs (**5a-d**) was determined by considering 9,10-diphenylanthracene as the standard. A 0.01 mM solution of 9,10-diphenylanthracene in ethanol ($\phi_f = 0.95$) was prepared for the measurement of fluorescence quantum yields with an error of ±10%. The absorbance values at excitation wavelengths for all the solutions (coumarin fluorogen-based PRPGs **5a-d** and 9,10-diphenylanthracene) were measured by a UV-Vis spectrophotometer. Photoluminescence (PL) emission spectra of all sample solutions were recorded using a fluorescence spectrophotometer at the respective excitation wavelengths of the compound. The final fluorescence quantum yield was determined using equation (**1**).

$$\frac{\Phi_s}{\Phi_r} = \frac{A_s}{A_r} \times \frac{(Abs)_r}{(Abs)_s} \times \frac{\eta_s^2}{\eta_r^2} \ldots\ldots\ldots\ldots\ldots(1)$$

Where Φ denotes quantum yield, A represents the area under the fluorescence curve, (Abs) represents absorbance, η denotes the refractive index of the medium, and subscripts s and r are for sample and reference, respectively.

Table. S1 Photophysical properties of coumarin fluorogen-based PRPGs **5a-d** in ACN

PRPGs	Absorption λ_{abs} (nm)[a]	Emission λ_{em} (nm)[b]	Molar absorptivity ε ($M^{-1}cm^{-1}$)[c]	Stokes shift (nm)[d]	Fluorescence quantum yield[e]
**5a**	437	517	32300	80	0.47
**5b**	443	562	30400	119	0.14
**5c**	458	538	23000	80	0.40
**5d**	560	653	10900	93	0.01

[a]Maximum absorption wavelength, [b]Maximum emission wavelength, [c]Molar absorption coefficient ($M^{-1}cm^{-1}$) at maximum absorption, [d]Difference between maximum absorption wavelength and maximum emission wavelength, [e]Fluorescence quantum yield (error limit within ±10%).

Solvent-dependent absorption and emission spectra

4 mL 0.01 mM solutions of all the coumarin fluorogen-based PRPGs were prepared by diluting using solvents of different polarities (e.g. Toluene, THF, ACN, DMSO, and Methanol) from stock solutions of 1mM. The absorption and emission spectra for all the solutions were recorded using a UV/vis spectrophotometer and a fluorescence spectrophotometer, respectively.

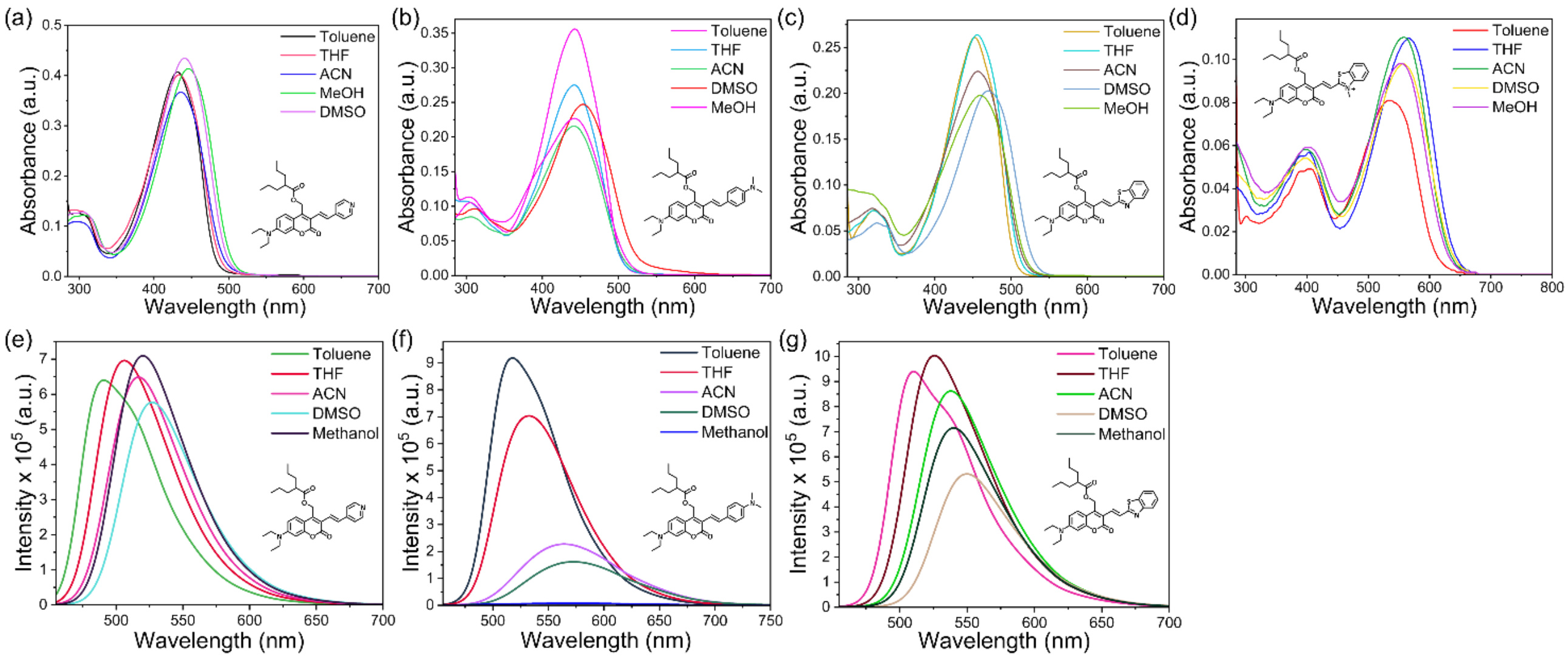

Figure S16 Absorption spectra (a) **5a**, (b) **5b**, (c) **5c**, (d) **5d**, and emission spectra of coumarin fluorogen-based PRPGs (e) **5a**, (f) **5b**, and (g) **5c** in solvents of different polarities. The final concentration of the coumarin fluorogen-based PRPGs was 0.01 mM. The emission spectra were recorded by exciting at 445 nm.

Viscosity sensing studies

4 mL 0.01 mM solutions of all the coumarin fluorogen-based PRPGs (**5a-d**) were prepared by diluting the 1 mM stock solution in DCM using a glycerol-methanol (v/v) mixture of various percentages (0, 20, 40, 60, 80% glycerol), and the emission spectra for all the solutions were recorded using a fluorescence spectrofluorometer.

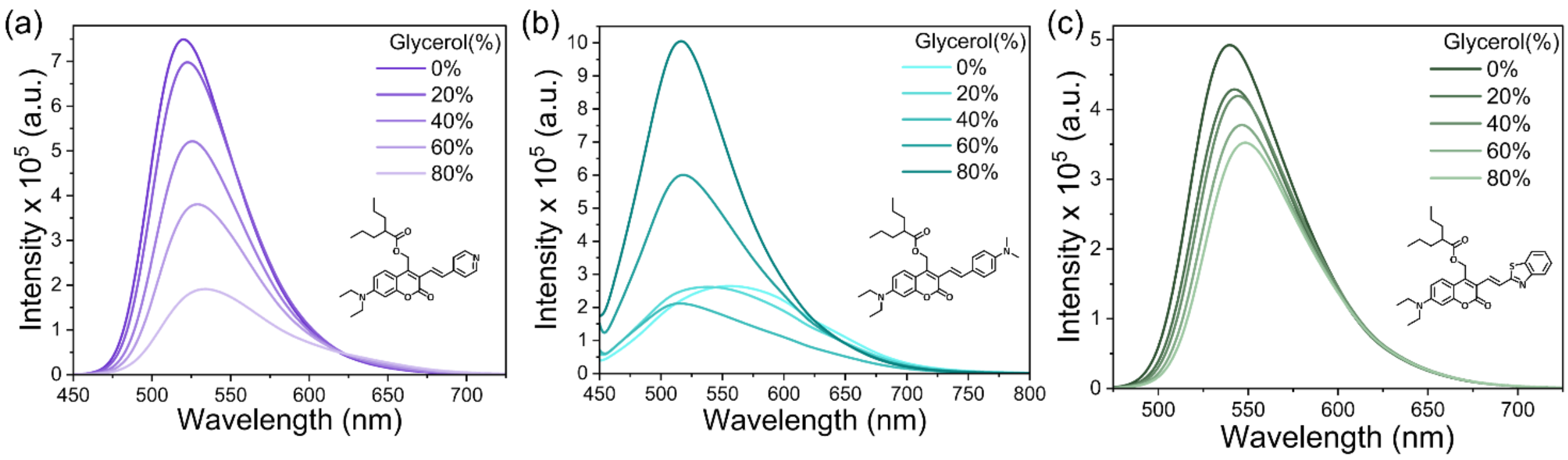

Figure S17 Emission spectra of coumarin fluorogen-based PRPGs (a) **5a**, (b) **5b**, and (c) **5c** in various percentages of glycerol-methanol (v/v) mixtures. The final concentration of the coumarin fluorogen-based PRPGs was 0.01 mM. The emission spectra were recorded by exciting at 445 nm.

To minimize the solvent polarity effect, solutions of ethylene glycol-glycerol (v/v) mixture were prepared as they have comparable dielectric constants (ethylene glycol: 37.7 and glycerol: 42.5). So, 0.01 mM solutions of various percentages of ethylene glycol-glycerol (v/v) (0, 20, 40, 60, 80% glycerol) for all the coumarin fluorogen-based PRPGs (**5a-d**) were prepared from 1 mM stock solutions in DCM, and the emission spectra were recorded in a fluorescence spectrofluorometer.

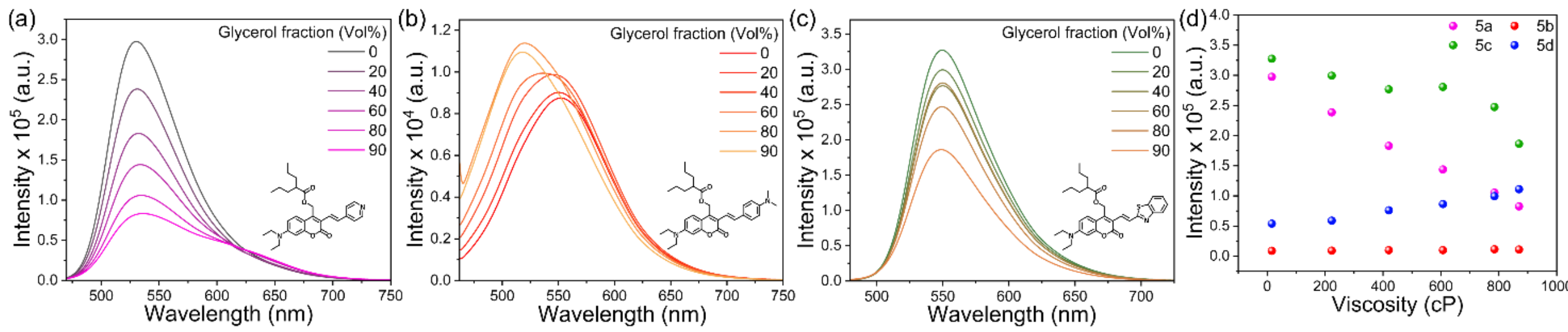

Figure S18 Emission spectra of coumarin fluorogen-based PRPGs (a) **5a**, (b) **5b**, and (c) **5c** in various percentages of ethylene glycol-glycerol (v/v) mixtures. (d) Variation in the emission intensity of coumarin fluorogen-based PRPGs (**5a-d**) with respect to the change in viscosity of the solvent medium. The final concentration of the coumarin fluorogen-based PRPGs was 0.01 mM. The emission spectra were recorded by exciting at a wavelength of 445 nm.

To plot the viscosity-dependent curves, the viscosity of the mixed solvents was calculated using equation (**2**).

$$\eta_{mix} = w_1.\eta_1 + w_2.\eta_2 \ldots\ldots\ldots\ldots\ldots(2)$$

Where η denotes the viscosity of the solvent systems, and w stands for weight fractions.
Again, the weight fraction can be calculated using equation (**3**).

$$w = \rho_1.V_1 \Big/ (\rho_1.V_1 + \rho_2.V_2) \ldots\ldots\ldots\ldots\ldots(3)$$

Where ρ is the density of the solvents, and V is the volume of the solvent in the mixture.

The viscosity-dependent curve was plotted using solvent viscosity as the X axis and emission intensity as the Y axis. The viscosity-dependent parameters χ_1 and χ_2 were calculated from the non-linear polynomial regression of the second-order equation (**4**).

$$I = C + \chi_1\eta + \chi_2\eta^2 \ldots\ldots\ldots\ldots\ldots(4)$$

Where I is the emission peak intensity, η is the viscosity of the solvent medium, C is the intercept of the curve, and χ_1, χ_2 are the viscosity sensitivity parameters where χ_1 is the linear dependency of the emission intensity on the change in viscosity and χ_2 is the non-linear relationship.

Table. S2 Viscosity and density of the solvents at 25 °C

Solvents	Viscosity cP	Density g/mL
Methanol	0.54	0.79
Ethylene glycol	15.5	1.11
Glycerol	954	1.26

Table. S3 Viscosity of the mixed solvents at 25 °C

Solvent mixture	Glycerol (Vol%)	0%	20%	40%	60%	80%	90%
Methanol	Viscosity (cP)[a]	0.54	272	492	673	825	892
Ethylene glycol		16	223	420	607	785	870

[a]Viscosity (cP) of the mixture of solvents at 25 °C (error limit within ±10%).

Emission spectra in various ACN/ Water (% v/v) mixtures

4 mL 0.01 mM solutions of all the coumarin fluorogen-based PRPGs (**5a-d**) with various ACN/ Water (% v/v) mixtures were prepared by diluting the stock solution of 1 mM in DCM, and the emission spectra were recorded in a fluorescence spectrofluorometer.

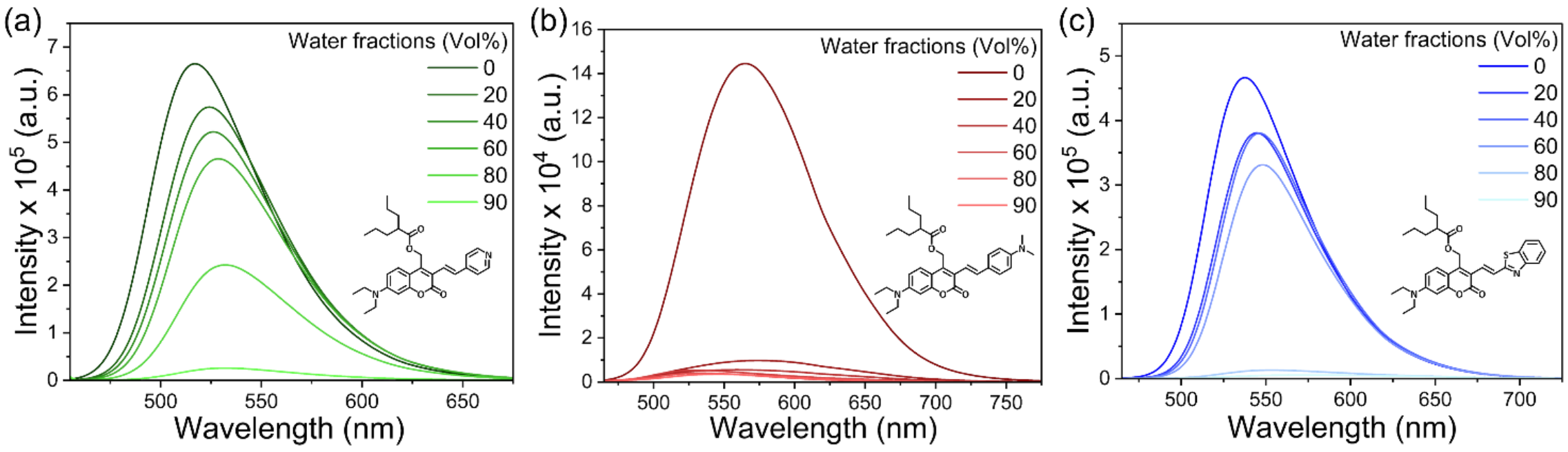

Figure S19 Emission spectra of coumarin fluorogen-based PRPGs (a) **5a**, (b) **5b**, and (c) **5c** in various ACN/ Water (% v/v) mixtures. The final concentration of the coumarin fluorogen-based PRPGs was 0.01 mM. The emission spectra were recorded at an excitation wavelength of 445 nm.

Fluorescence lifetime

The TCSPC measurement, a time-resolved emission measurement, provides information about the excited-state lifetime through the excited-state decay dynamics of a fluorescent molecule. In the TCSPC measurement module, we have used picosecond diode LASERs with peak wavelengths of 510 nm and 440 nm as the excitation LASER source. The fluorescence signals generated are detected using the Hamamatsu MCP PMT (3809U) detector at the polarizer angle of ~54.7°, which is called magic angle polarization. The TCSPC decays are collected in reverse mode and analysed with respect to the prompt (or instrument response function (IRF)) that has been taken. The IBH DAS-6 software has been utilized to fit the collected decays using the respective order of exponential equations to get the subsequent lifetime values and contributions. The average lifetimes (τ_{avg}) of **5a-d** were calculated by using equation (**5**).

$$\tau_{avg} = \sum a_i \tau_i \quad \ldots\ldots\ldots\ldots\ldots (5)$$

Where τ_i is the lifetime component, and a_i is the respective amplitude. Contributions with i represent the order of the exponential equation used to fit the lifetime decay trace, and $\sum a_i = 1$.

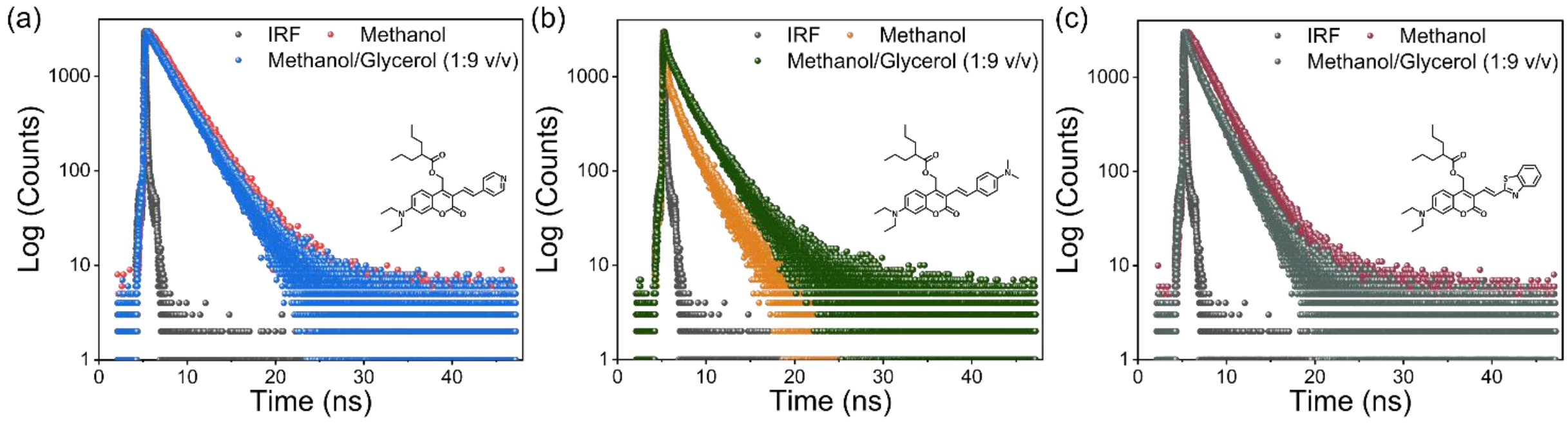

Figure S20 Time-correlated single photon counting decay profile of coumarin fluorogen-based PRPGs (a) **5a**, (b) **5b**, and (c) **5c** at an excitation wavelength of 440 nm in methanol and methanol-glycerol (1:9 v/v) mixture. The final concentration of the coumarin fluorogen-based PRPGs was 0.01 mM.

Table. S4 Average fluorescence lifetime of coumarin fluorogen-based PRPGs **5a-d**

Solvent systems	Average lifetimes τ_{avg} (ns)[a]			
	**5a**	**5b**	**5c**	**5d**
Methanol	2.76	0.36	2.37	0.54
MeOH/Glycerol (1:9 v/v)	2.18	0.81	1.27	1.31

Average lifetime (ns) in methanol and methanol/glycerol mixture at an excitation wavelength of 440 nm for PRPGs **5a-c** and 510 nm for coumarin fluorogen-based PRPG **5d** (error limit within ± 10%).

Temperature-dependent emission spectra with variation in solvent viscosity

Emission intensities for coumarin fluorogen-based PRPGs **5b** and **5d** were recorded in glycerol-methanol mixtures at a temperature range of 25-45 °C with a 5 °C variation. The methanol-glycerol mixture was chosen as methanol has low viscosity (0.54 cP at 25 °C), which is significantly less viscous than ethylene glycol (16 cP at 25 °C). So, mixing of methanol and glycerol provided a clearer investigation of temperature-dependent viscosity, as the viscosity of the solvent is highly temperature sensitive.

To get a clear idea about molecular rotation, we considered a model based on the Arrhenius equation (**6**).

$$k_{rot}=A(\eta)e^{\frac{-E_a}{k_B T}}\ldots\ldots\ldots\ldots\ldots(6)$$

Here, k_{rot} is the rate of bond rotation, which is a function of solvent viscosity (η) and temperature (T). E_a is the rotational energy barrier.

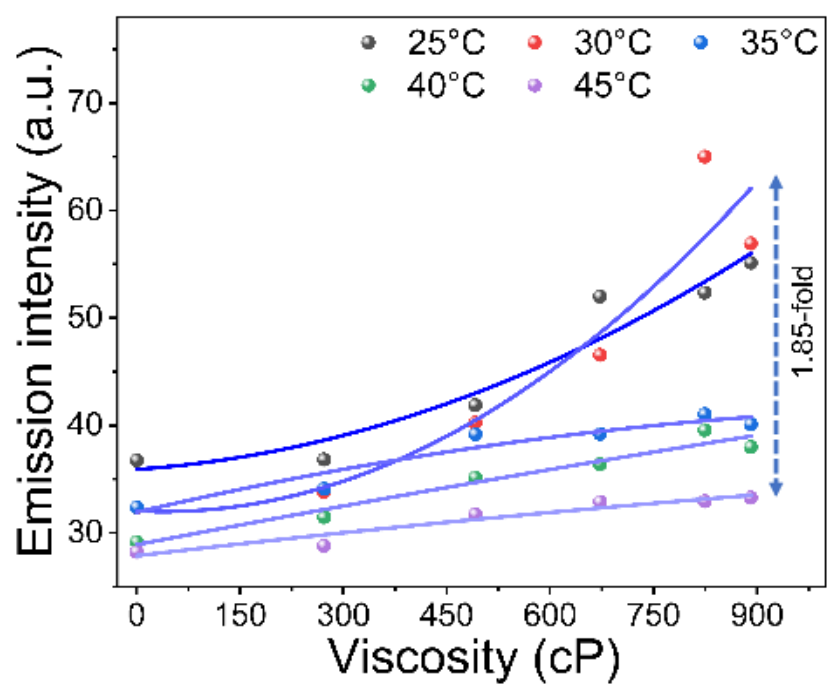

Figure S21 Temperature-dependent emission spectra of coumarin fluorogen-based PRPG **5b** in different methanol-glycerol percentages according to viscosity indices. The final concentration of the probe was 0.01 mM.

6. Determination of incident photon flux (I_0) for LED lamps:

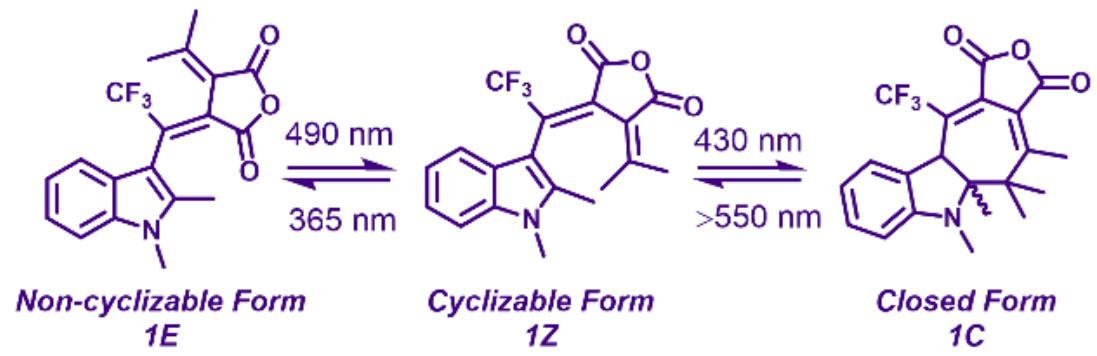

Scheme Photochemical isomers of indolylfulgide.

The photo flux of LED lamps was determined using a 0.1 mM solution of Fulgide actinometer in toluene as per the reported procedure.[2] At first, the prepared solution of Fulgide was irradiated with the corresponding light for 45 sec to get its photostationary state and absorption data were recorded spectrochemically. The existence of a photostationary state was confirmed by no change in absorbance value after prolonged irradiation. Then, the solution was irradiated with the required LED light, and decreased absorption was recorded at a regular interval of time (1 sec). The linear change in absorbance was plotted against time to get the slope for the photochemical reaction of the Fulgide due to irradiation of the particular LED lamp. So, by using the following equation (**7**), the intensity of incident light can be calculated.

$$I=\frac{\Delta A}{t}\times\frac{V}{\varepsilon\times d\times\phi}\times 6.023\times10^{23}\ldots\ldots\ldots\ldots\ldots(7)$$

Where $\frac{\Delta A}{t}$ is the slope of the curve, V is the sample volume in dcm^3, ε is the molar absorption coefficient at the wavelength corresponding to the LED light, ϕ is the reported quantum yield of the particular photochemical reaction near the emitting wavelength of the LED light, and d is the optical pathway in cm. This will give the value in Einstein s^{-1}, which is further converted to quanta s^{-1} by multiplying 6.023×10^{23}.

Photon flux (I_0) for blue LED lamp (456 ± 10 nm)

The photo flux of blue light LED (456 ± 10 nm) was determined by the above-described procedure. The prepared Fulgide solution was first irradiated with 525 nm LED light for 45 sec to get its photostationary state, which is mainly the Cyclizable form (**1Z**) and absorption data was recorded spectrochemically at ~ 427 nm. Then, the solution was irradiated with 456 ± 10 nm LED light, and decreased absorption at ~ 427 nm was recorded at a regular interval of time (1 sec). The slope change in absorbance was plotted against time denotes the rate of change of Cyclizable form (**1Z**) to Closed form (**1C**). From this plot, the decrease of Cyclizable form (**1Z**) at a particular time can be calculated. So, using equation (**2**), the incident intensity of 456 ± 10 nm LED light was calculated as 2.89×10^{16} quanta s^{-1}.

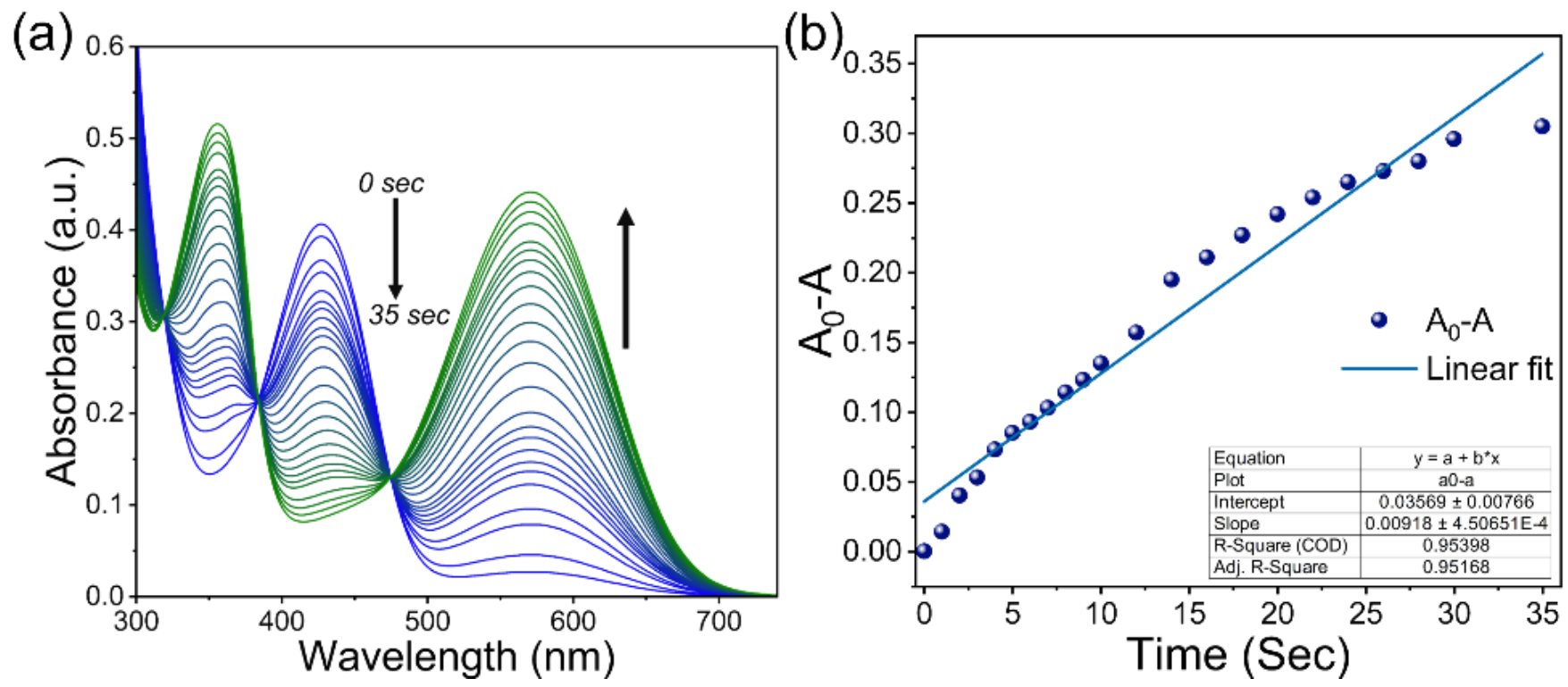

Figure S22 (a) Time-dependent absorption spectra, and (b) Linear fit plot of ΔA vs Time (s) for Fulgide actinometer irradiated with 456 ± 10 nm LED light.

Photon flux (I_0) for green LED lamp (525 ± 10 nm)

The photo flux of blue light LED (525 ± 10 nm) was determined by the above-described procedure. The prepared Fulgide solution was first irradiated with 456 nm LED light for 45 sec to get its photostationary state, which is mainly the Closed form (**1C**) and absorption data was recorded spectrochemically at ~ 572 nm. Then, the solution was irradiated with 525 ± 10 nm LED light, and decreased absorption at ~ 572 nm was recorded at a regular interval of time (1 sec). The slope change in absorbance was plotted against time denotes the rate of change of Closed form (**1C**) to Cyclizable form (**1Z**). From this plot, the decrease of Closed form (**1C**) at a particular time can be calculated. So, using equation (**2**), the incident intensity of 525 ± 10 nm LED light was calculated as 2.51×10^{15} quanta s^{-1}.

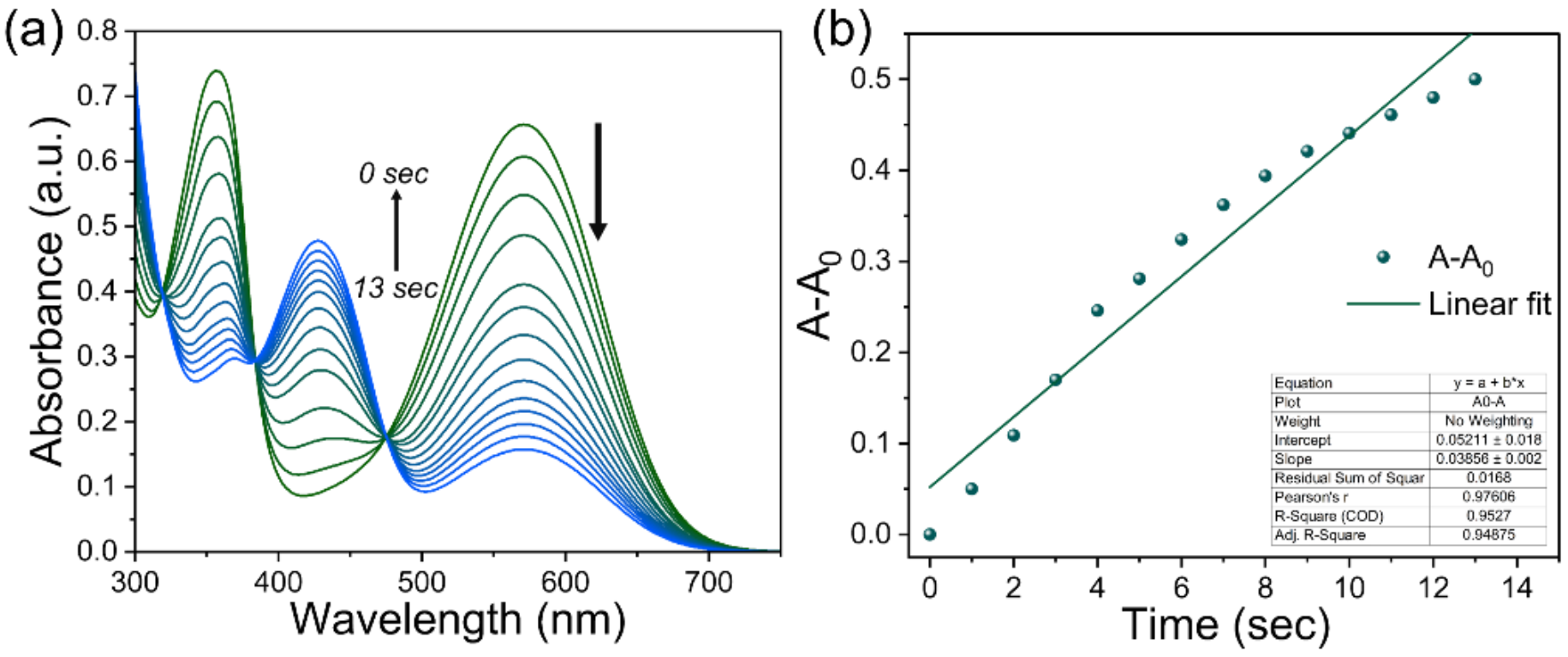

Figure S23 (a) Time-dependent absorption spectra, and (b) Linear fit plot of ΔA vs Time (s) for Fulgide actinometer irradiated with 525 ± 10 nm LED light.

7. General photochemical studies:

Photorelease monitoring of coumarin fluorogen-based PRPGs (5a-d) and determination of photouncaging quantum yield using ^1H NMR spectroscopy

The photouncaging quantum yield for coumarin fluorogen-based PRPGs (**5a-d**) was determined by proton NMR spectroscopy using 1,2-dichloroethane as an internal standard. 1.8 mg of the coumarin fluorogen-based PRPGs were dissolved in 0.7 ml of NMR solvents (ACN-d_3 and D_2O). The internal standard, 1,2-dichloroethane, was added in an equimolar amount of **5a-d**. The decrease in concentration was calculated with respect to the -CH_2 peak intensity of the esters. The quantum yield of uncaging was calculated using equation (8).

$$\Phi_{FP}=\Phi_{act}\times\frac{k_{FP}}{k_{act}}\times\frac{F_{act}}{F_{FP}}\ldots\ldots\ldots\ldots\ldots(8)$$

Where the subscript FP and act denote coumarin fluorogen-based PRPG and actinometer, respectively, Φ is the relative photolysis quantum yield, *k* is the photolysis rate constant, and F represents the fraction of light absorbed. The photorelease quantum yield was calculated with respect to the photodegradation of the starting material.

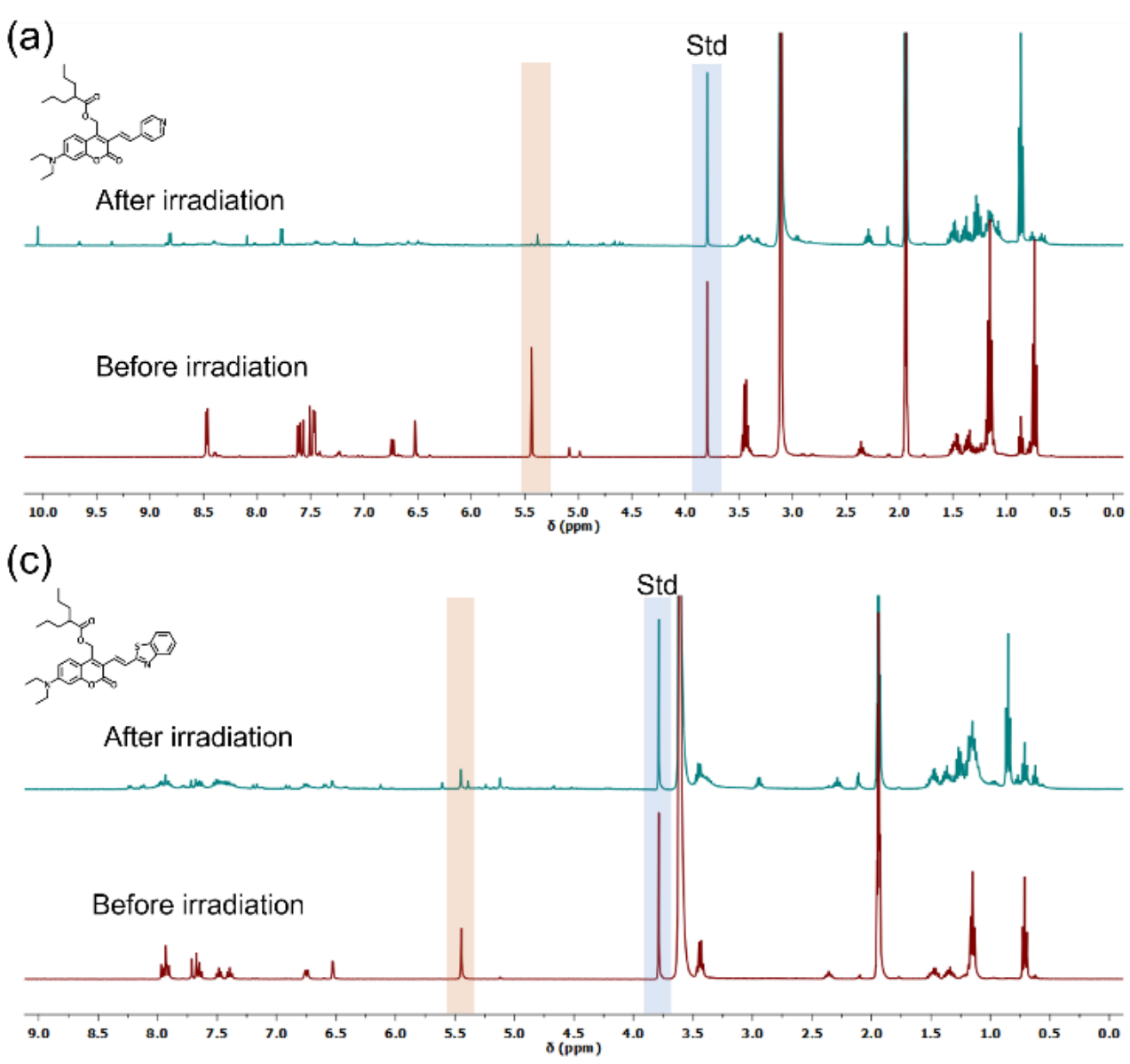

Figure S24 Photolysis monitoring by ^{1}H NMR (400 MHz) for coumarin fluorogen-based PRPGs (a) **5a** (1.8 mg in 0.7 mL ACN-d$_3$/ D$_2$O 9:1 v/v), (b) **5b** (1.7 mg in 0.7 mL ACN-d$_3$/ D$_2$O 9:1 v/v), (c) **5c** (1.8 mg in 0.7 mL ACN-d$_3$/ D$_2$O 4:1 v/v), and (d) **5d** (1.8 mg in 0.85 mL ACN-d$_3$/ D$_2$O 1:9 v/v) in presence of 1,2-dichloroethane as internal standard.

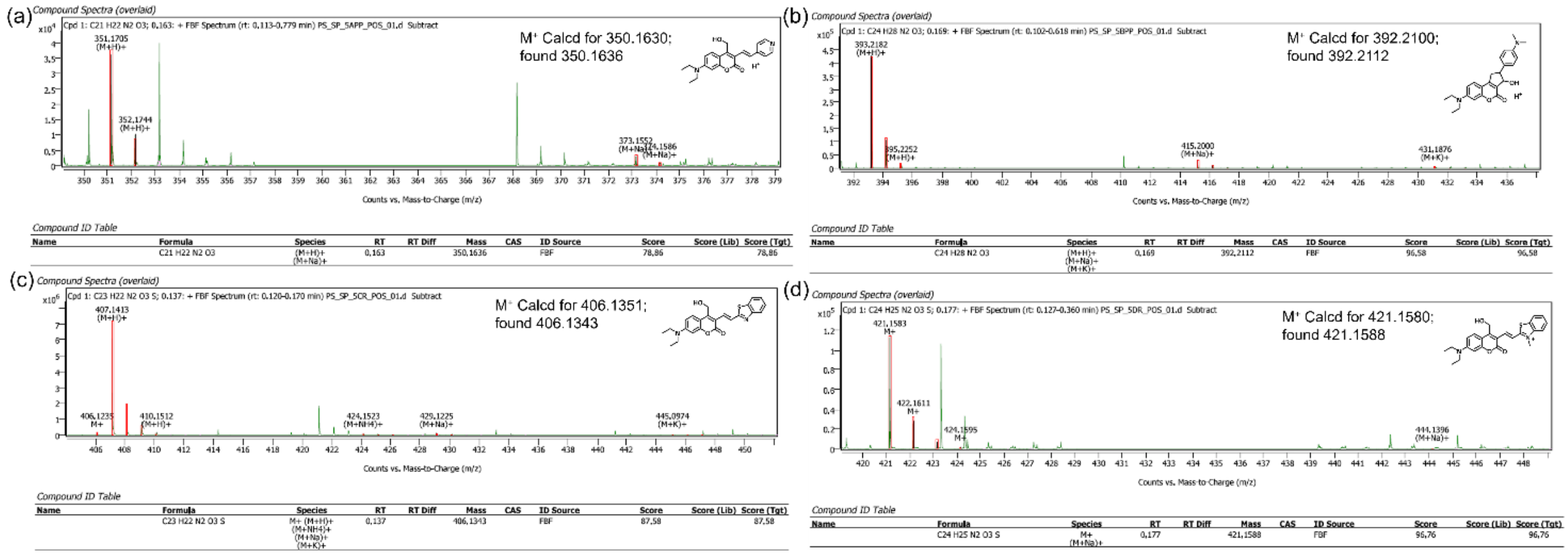

Figure S25 HRMS spectra of photolyzed reaction mixtures of coumarin fluorogen-based PRPGs (a) **5a**, (b) **5b**, (c) **5c**, and (d) **5d**.

Table. S5 Photorelease quantum yield for coumarin fluorogen-based PRPGs (**5a-d**)

	**5a**	**5b**	**5c**	**5d**
Photorelease quantum yield (%)	0.18	0.13	0.03	0.11

Isolation and characterization of photoproduct 5d(PP)

The photolysis of coumarin fluorogen-based PRPG **5d** was monitored using HRMS, and the formed photoproduct **5d(PP)** was isolated using column chromatography (DCM/ Methanol 1:50 v/v). The isolated **5d(PP)** was characterized by proton NMR spectrum and HRMS. ^{1}H NMR (400 MHz, CDCl$_3$) δ 8.04 (dd, *J* = 13.2, 8.4 Hz, 2H), 7.77 (t, *J* = 7.8 Hz, 1H), 7.65 (t, *J* = 7.7 Hz, 1H), 7.13 (d, *J* = 8.9 Hz, 1H), 6.56 (dd, *J* = 9.0, 2.3 Hz, 1H), 6.46 (d, *J* = 2.3 Hz, 1H), 5.67 – 5.61 (m, 1H), 5.42 (dd, *J* = 15.2, 4.3 Hz, 1H), 5.14 (dd, *J* = 15.2, 3.0 Hz, 1H), 4.35 (s, 3H), 4.14 (d, *J* = 4.1 Hz, 2H), 3.40 – 3.35 (m, 4H), 1.17 (t, *J* = 7.1 Hz, 6H). HRMS (ESI) m/z: [M]$^+$ calcd for $C_{24}H_{25}N_2O_3S^+$ 547.2631; found 547.2632.

Also, the methoxy photoproduct (**5d(PP′)**) was detected in photolysis mixture by HRMS when the photolysis of **5d** was carried out in Methanol/water (1:9) solvent.

(a)

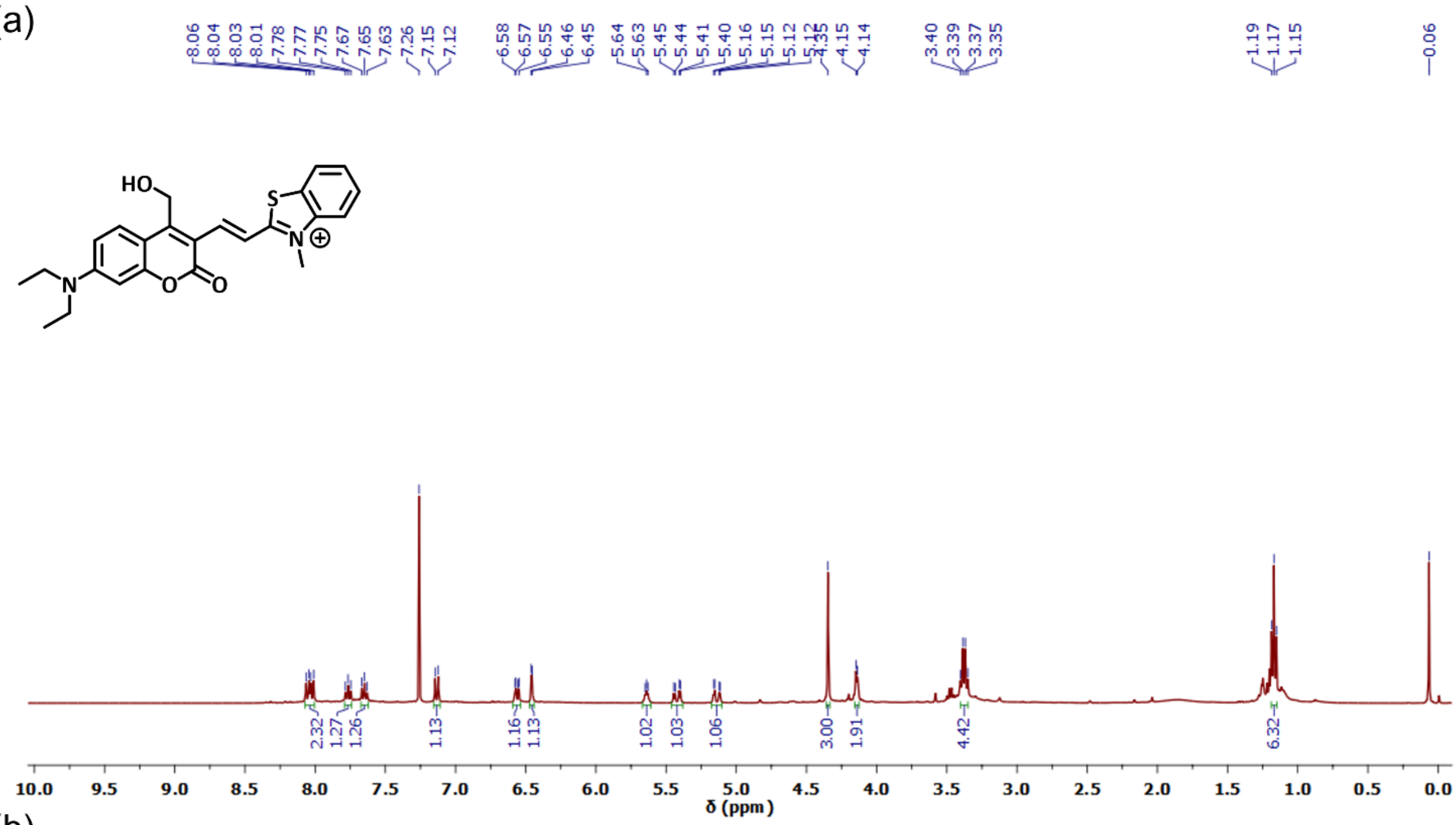

(b)

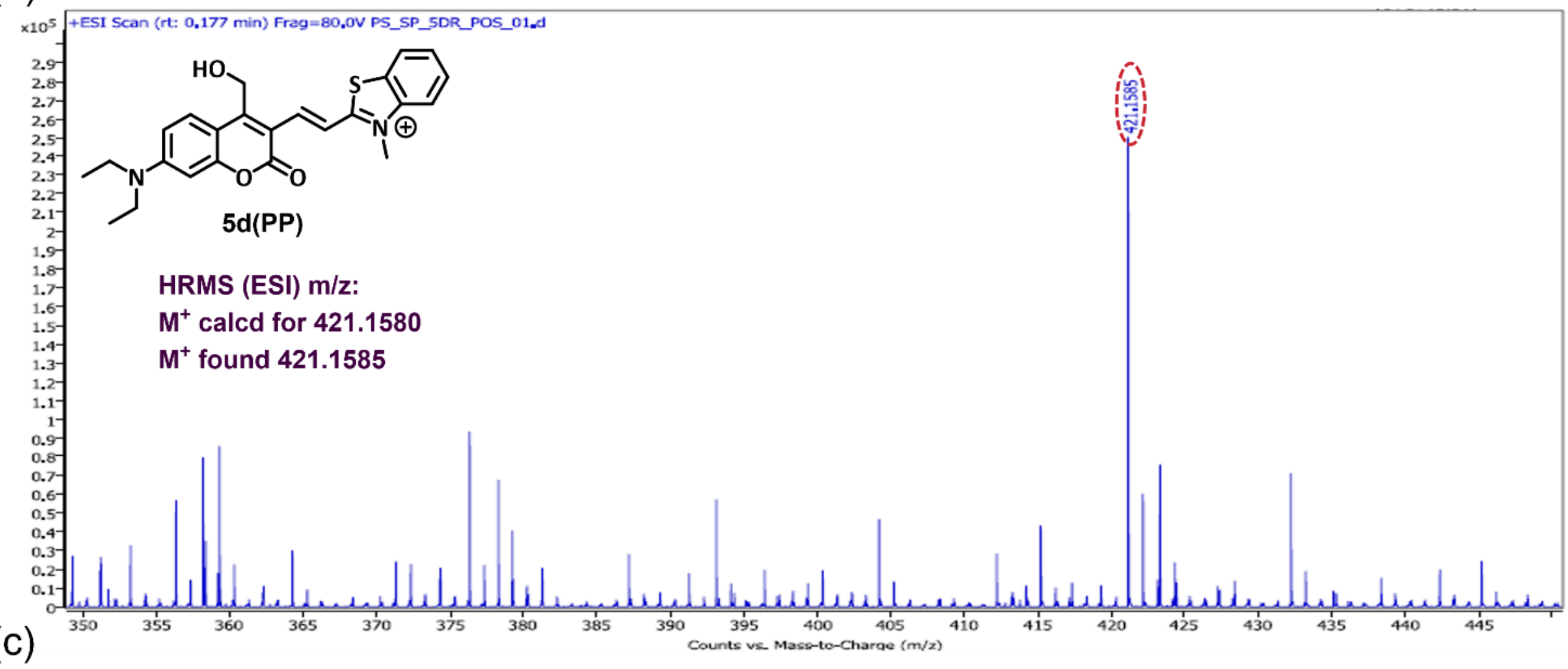

(c)

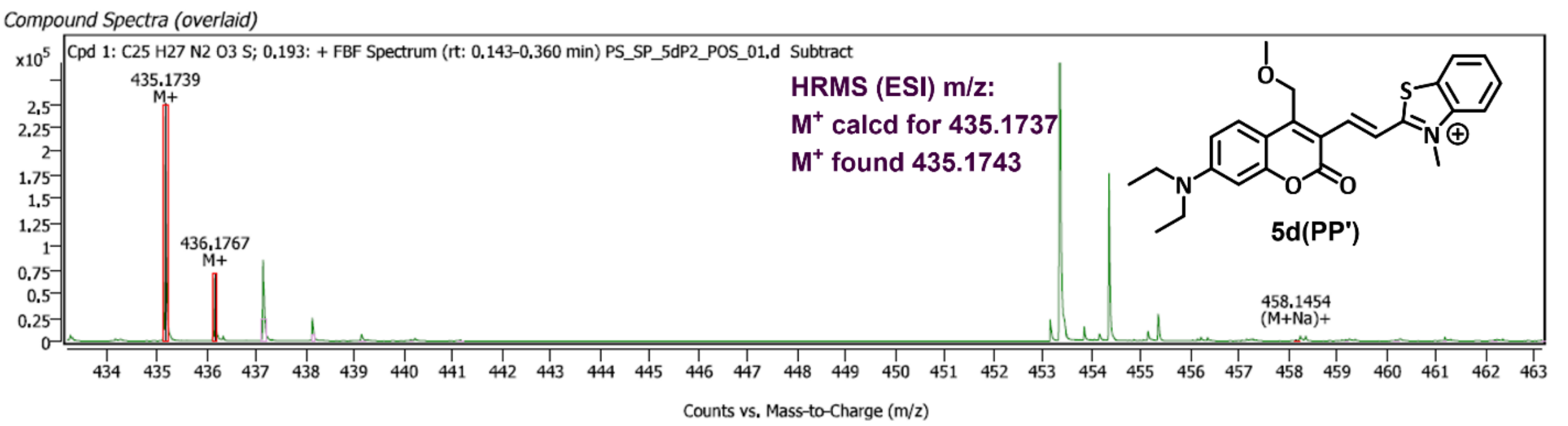

Compound ID Table

Name	Formula	Species	RT	RT Diff	Mass	CAS	ID Source	Score	Score (Lib)	Score (Tgt)
	C25 H27 N2 O3 S	M+ (M+Na)+	0.193		435.1743		FBF	72.21		72.21

Figure S26 (a) ^{1}H NMR (400 MHz) of photoproduct **5d(PP)** in $CDCl_3$. (b) Photolysis mixture HRMS of **5d**. (c) Detection of -OMe Photoproduct (**5d(PP′)**) by HRMS in the photolysis mixture when Methanol-Water (1:9) was used as solvent.

Quenching study for coumarin fluorogen-based PRPG 5d

To determine whether the coumarin fluorogen-based PRPG **5d** showed singlet state or triplet state chemistry, the photolysis was monitored by absorption spectroscopy in the presence of 1 mM *N,N′*-

Di-*n*-octyl-3,4,9,10-perylenetetracarboxylic Diimide as singlet state quencher and Potassium sorbate as triplet state quencher with 0.01 mM **5d**.

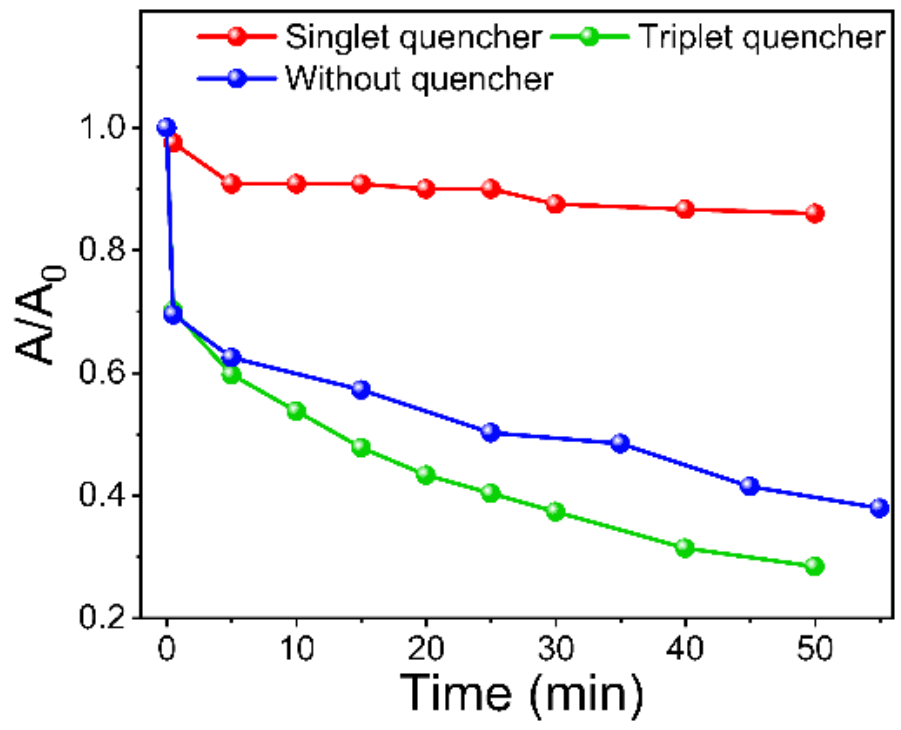

Figure S27 Relative absorbance (A/A_0) vs time (min) curve for **5d** in the presence of singlet and triplet quencher. The final concentration of **5d** was 0.01 mM, and the quenchers were 1 mM.

Photolysis monitoring of coumarin fluorogen-based PRPGs (5a-d) by absorption spectroscopy

To study the photoisomerization and photouncaging during the light irradiation, the course of photolysis was monitored using absorption spectroscopy at defined time intervals in different solvent mixtures (ACN, ACN-water, and methanol-glycerol) for systematic variation of the aggregated state. 0.01mM 4 mL solutions of coumarin fluorogen-based PRPGs (**5a-d**) were prepared by diluting 1 mM stock solutions. The prepared solutions were irradiated with blue (456 ± 10 nm) and green (525 ± 10 nm) LEDs for **5a-c** and **5d**, respectively, and the data were recorded using the Shimadzu UV-1900i UV/vis spectrophotometer at different time intervals. The change in absorption resulted either from photoisomerization or photoproduct formation.

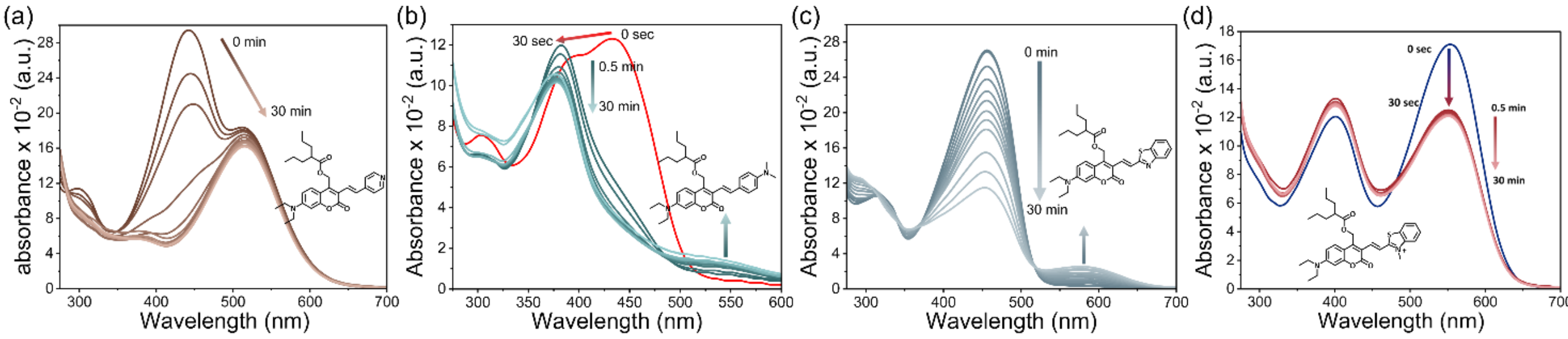

Figure S28 Photolysis of coumarin fluorogen-based PRPGs (a) **5a**, (b) **5b**, (c) **5c**, and (d) **5d** in ACN. The final concentration of the coumarin fluorogen-based PRPGs was 0.01 mM.

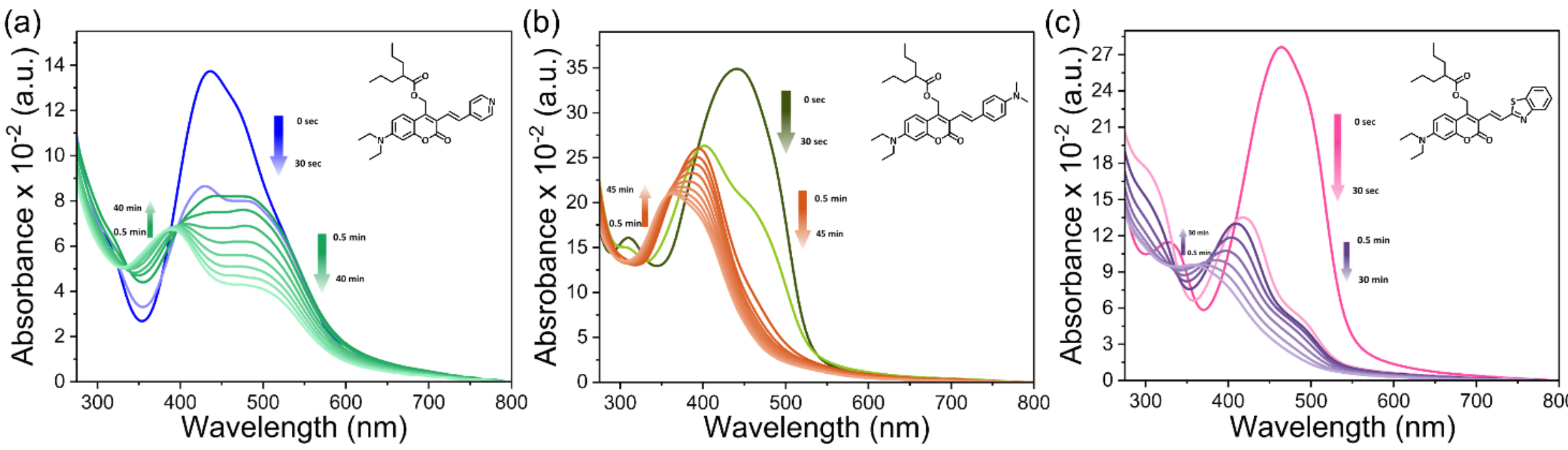

Figure S29 Photolysis of coumarin fluorogen-based PRPGs (a) **5a**, (b) **5b**, and (c) **5c** in ACN-water (1:19 v/v). The final concentration of the coumarin fluorogen-based PRPGs was 0.01 mM.

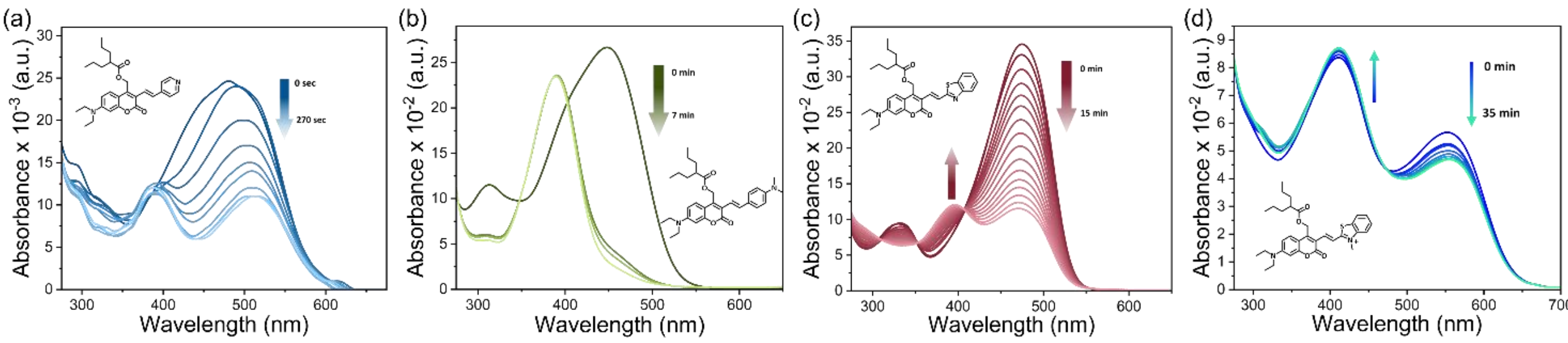

Figure S30 Photolysis of coumarin fluorogen-based PRPGs (a) **5a**, (b) **5b**, (c) **5c**, and (d) **5d** in methanol-glycerol (1:9 v/v). The final concentration of the coumarin fluorogen-based PRPGs was 0.01 mM.

Spatio-temporal control over the photouncaging process, "ON-OFF" study

Temporal control over the photouncaging process of coumarin fluorogen-based PRPG **5d** was monitored by a decrease in the peak area of the ester peak at 5.69 ppm in ^{1}H NMR (CD_3CN/D_2O 1:19 v/v, 1.8 mg in 0.85 ml). Spatial control was subsequently validated through confocal imaging of MPP^+-treated SH-SY5Y cells. In a 6-well plate, 3 wells were covered with aluminium foil, and it was irradiated with 525 nm LED light. The confocal image confirmed a decrease in the fluorescence of **5d** only in the uncovered wells, which confirmed the localized activation of the system.

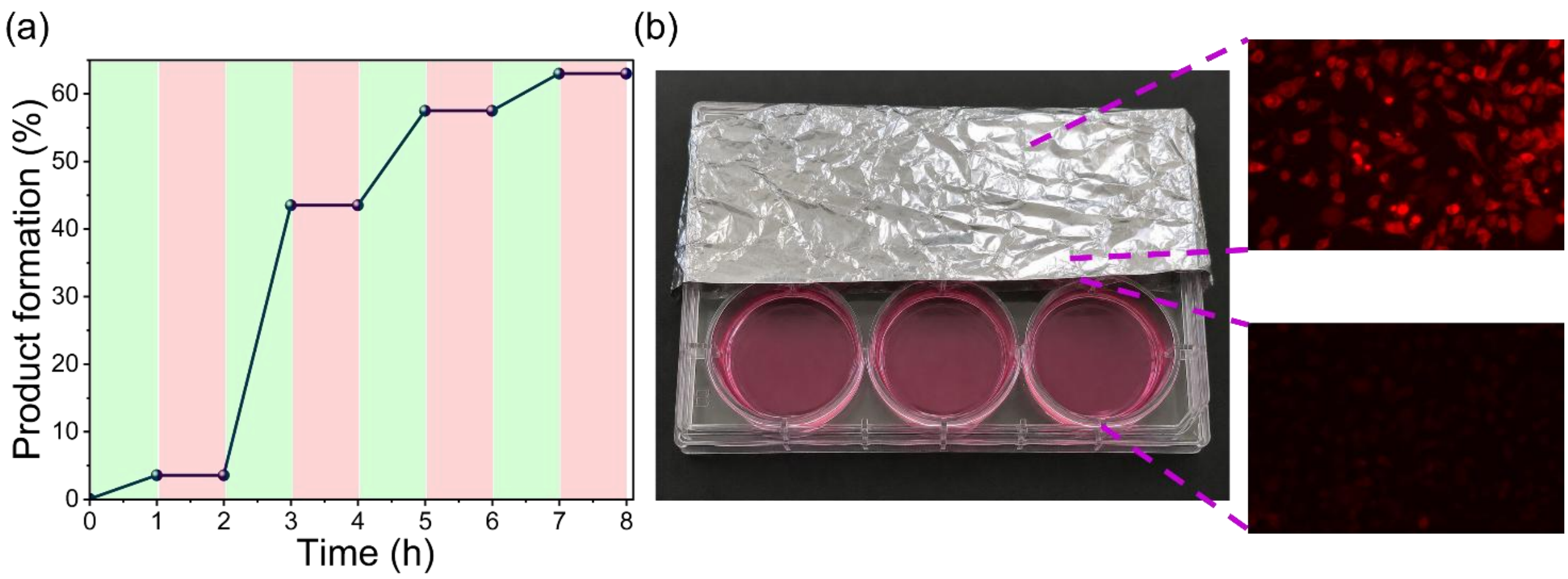

Figure S31 (a) "ON-OFF" study of **5d**, with 1.8 mg in 0.85 mL of CD_3CN/D_2O (1:19 v/v) solution to demonstrate temporal control. (b) Cellular imaging after irradiation of a covered and an uncovered 6-well plate to demonstrate spatial control.

8. TDP-43 protein aggregation and general characterization:

ThT fluorescence experiment. A 1 mM stock solution of Thioflavin T (ThT) was prepared in ACN. The stock solution was further diluted by 500 µL of PBS buffer (pH 7.4) to maintain the final concentration of 0.01 mM. The emission of ThT at 485 nm was measured using a Hitachi F7000 fluorescence spectrophotometer at an excitation wavelength of 440 nm.

TEM measurement. A 1 µM solution of aggregated TDP-43 protein in PBS (pH 7.4) was drop-cast onto a 300-mesh carbon-coated copper grid and allowed to dry. Then, on the same grid, a water solution of uranyl acetate (2% w/v) was added as negative staining. The sample was kept at room temperature to dry, and kept in a desiccator for 24 h. A similar procedure was followed for the sample preparation of the TDP-43 protein after irradiation of the aggregated solution. The TEM analysis was measured using a JEOL JEM2100F instrument at 200 kV.

Circular dichroism spectroscopy. A 500 µL solution was prepared in PBS buffer (pH 7.4) containing 1 µM TDP-43 protein peptide and 0.01 mM of the coumarin fluorogen-based PRPG **5d**. CD spectra were acquired and monitored on a JASCO instrument (Model J-815–150S) at 25 °C for the initial day (0 day), after ageing of 9 days, and after irradiation for different times (10, 20, 40 min) followed by 3 days of incubation at 37 °C at a wavelength range of 180-220 nm.

Aggregation of TDP-43 protein

The TDP-43 protein monomer was prepared by dissolving the Human TDP-43 synthetic peptide in PBS buffer (pH 7.4) with a final concentration of 1 µM. The resulting solution was shaken at 37 °C for 9 days. The protein aggregation was confirmed by the ThT fluorescence experiment and TEM analysis.

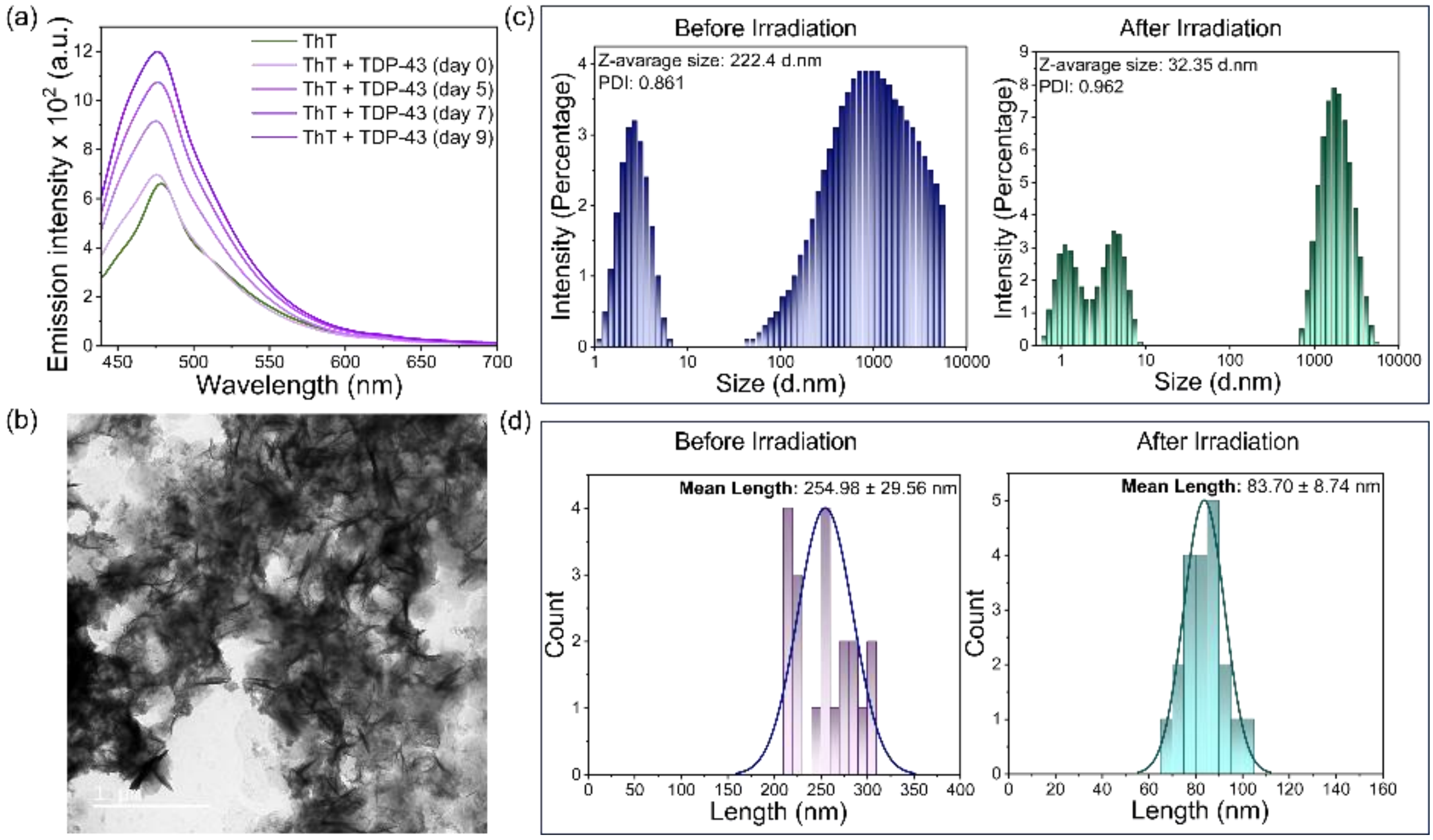

Figure S32 TDP-43 protein aggregation monitoring by (a) ThT-assay fluorescence (λ_{ex} = 412 nm), (b) TEM analysis in PBS buffer (pH 7.4). Fibril size distribution from (c) DLS experiment, and (d) TEM image before and after irradiation with **5d**. The final concentration of the TDP-43 protein monomer was 1 μM.

TDP-43 protein aggregation sensing by coumarin fluorogen-based PRPGs (5a-d)
1 mM stock solutions of coumarin fluorogen-based (**5a-d**) were diluted with a 1 μM PBS buffer (pH 7.4) solution of TDP-43 protein to get the final concentration of 0.01 mM. The emission for all the coumarin fluorogen-based PRPGs (**5a-d**) was recorded using a Hitachi F7000 fluorescence spectrophotometer at day 0 and day 9 to check the sensing capabilities.

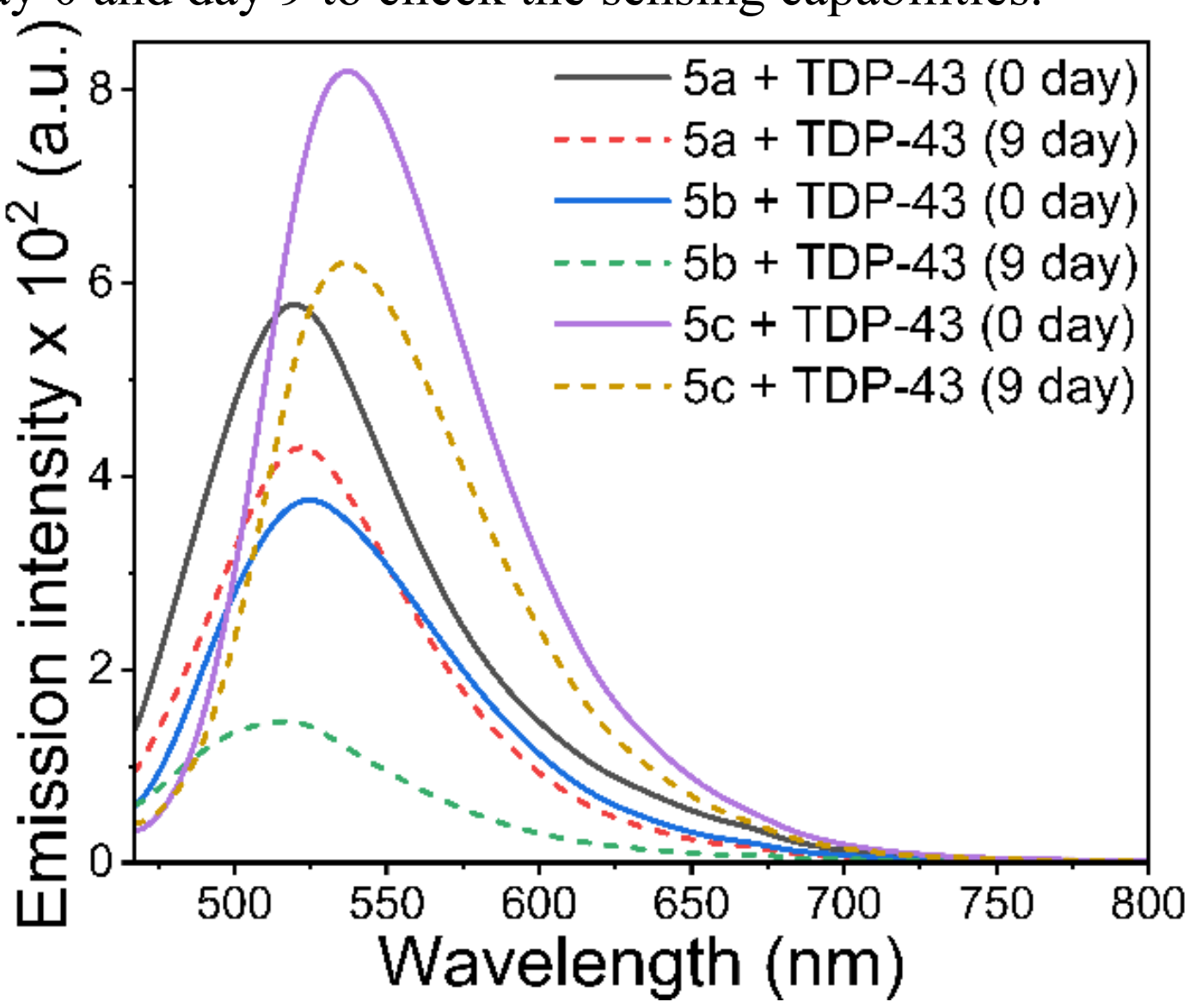

Figure S33 The emission spectra of coumarin fluorogen-based PRPGs (**5a-c**) in PBS buffer (pH 7.4) in the presence of TDP-43 protein on day 0 and day 9. The emission data were recorded by exciting the coumarin fluorogen-based PRPGs at their absorption maxima. The final concentration for all the coumarin fluorogen-based PRPGs was 0.01 mM.

Controlled experiment for coumarin fluorogen-based PRPG 5d
Controlled experiment of **5d** was performed to check whether it destroys the aggregated structure or not. CD and emission spectra of coumarin fluorogen-based **5d** with a 1 μM PBS buffer (pH 7.4) solution of TDP-43 protein were recorded by varying the concentration of **5d**.

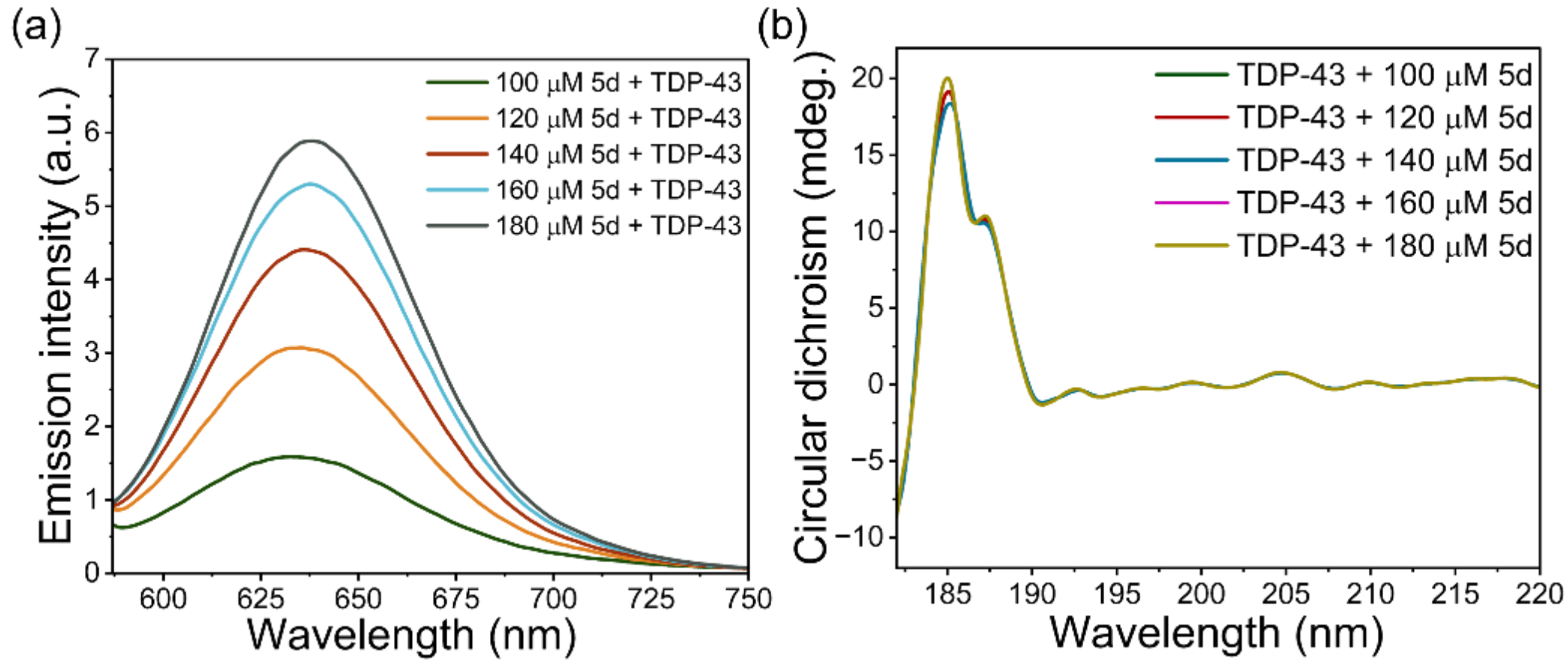

Figure S34 (a) Emission spectra (λ_{ex} 560 nm), and (b) CD spectra of **5d** with various concentrations in the presence of 1 μM TDP-43 protein in PBS buffer (pH 7.4).

9. Biological experiments:

Cell viability studies for coumarin fluorogen-based PRPGs

Cell viability of the coumarin fluorogen-based PRPGs (**5a-d**) and valproic acid was measured using the 3-(4,5-dimethylthiazol-2-yl)-2,5-diphenyltetrazolium bromide (MTT) assay. 1.5 × 10^7 cells/ml were seeded in a 96-well plate for overnight. Then, the compounds were treated at different concentrations for 24 hours and were further exposed to LED light irradiation to obtain data with and without light exposure. Following treatment, MTT solution (5 mg/mL) was added and incubated for 3 h. The resulting formazan crystals were dissolved in a 1:1 mixture of dimethyl sulfoxide (DMSO), and absorbance was measured at 595 nm using a microplate reader. Cell viability was expressed as a percentage using the formula: % viability = [(A_{595} (treated cells) − background) / (A_{595} (untreated cells) − background)] × 100.

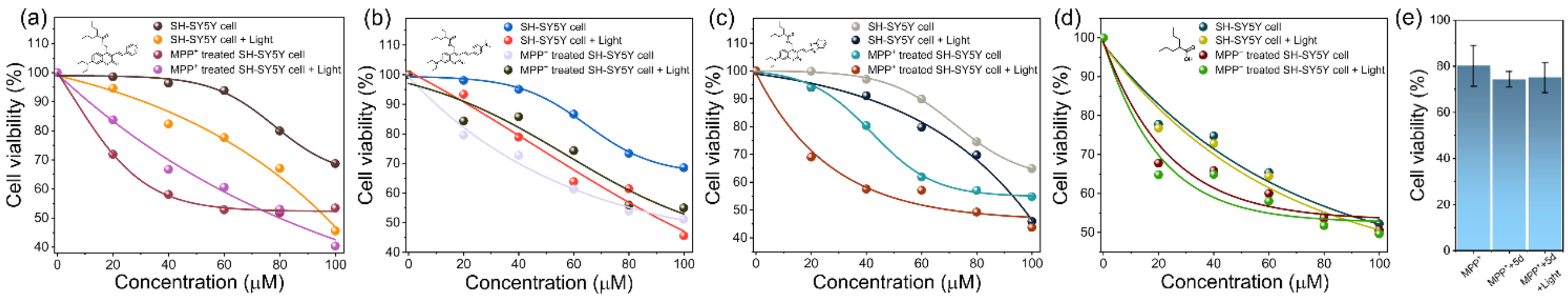

Figure S35 Cell viability studies of coumarin fluorogen-based PRPGs (a) **5a**, (b) **5b**, (c) **5c**, and (d) Valproic acid (VPA) in the SH-SY5Y neuroblastoma cell line and MPP^+ treated SH-SY5Y neuroblastoma cell line before and after light irradiation. (e) Bar diagram of cell viability study for 70 μM **5d** and 100 μM MPP^+ in different conditions (Error limit within ± 10%).

Colocalization study

For the colocalization study of fluorogen-based PRPG **5d**, we used commercially available Thioflavin T (ThT) as a standard, which can sense protein aggregation and viscosity sense of cellular environment. The SH-SY5Y cells and MPP^+ -treated SH-SY5Y cells were seeded at a density of 5000 cells per well one day before the treatment. Then the cells were treated with **5d** (100 μM) and ThT (5 μM) for 5 h. After treatment, cells were subsequently washed with 1× PBS, and confocal imaging was performed before and after irradiation for 40 min with a green LED (525 ± 5 nm).

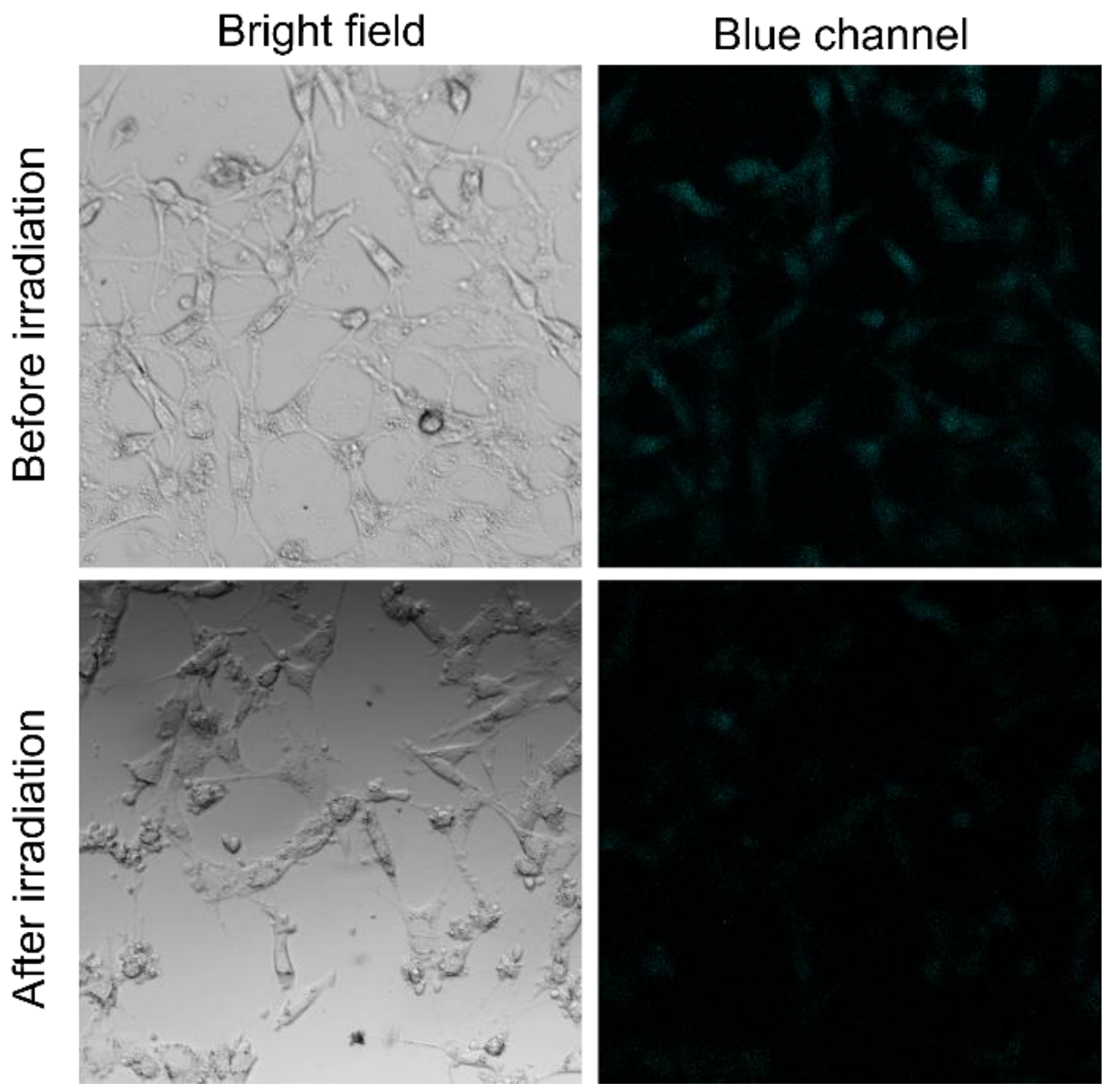

Figure S36 Intracellular defibrillation monitoring before and after irradiation by confocal imaging using Thioflavin T as standard.

10. Computational studies:

All calculations were performed in DFT, with M062X functional[1] and TZVP basis set.[2] An empirical dispersion factor[3] was also added. For TDDFT calculations, CAM-B3LYP basis set[4] was used instead of M062X for better performance.[5] Two solvent systems were considered, viz. acetonitrile: water at 1:9 ratio and methanol: glycerol at 1:9 ratio. These solvents were considered in the SCRF formalism[6] with integral equation formalism polarisable continuum model (IEFPCM) approximation,[7] with dielectric constants at 75.0 for the first and 41.0 for the second set, with radii of the spheres used in the model kept at 0.50 and 2.0 units. In the case of the S_1 state and the transition states, the SV basis set[8] is used for optimization as it is much smaller. M062X functional with CAM-B3LYP and TZVP basis sets were used at optimized geometries for TDDFT and MO figures, respectively. The percentage contribution of the major component of the electronic transition is given in brackets in **Table S6**. H, L represent HOMO and LUMO, respectively.

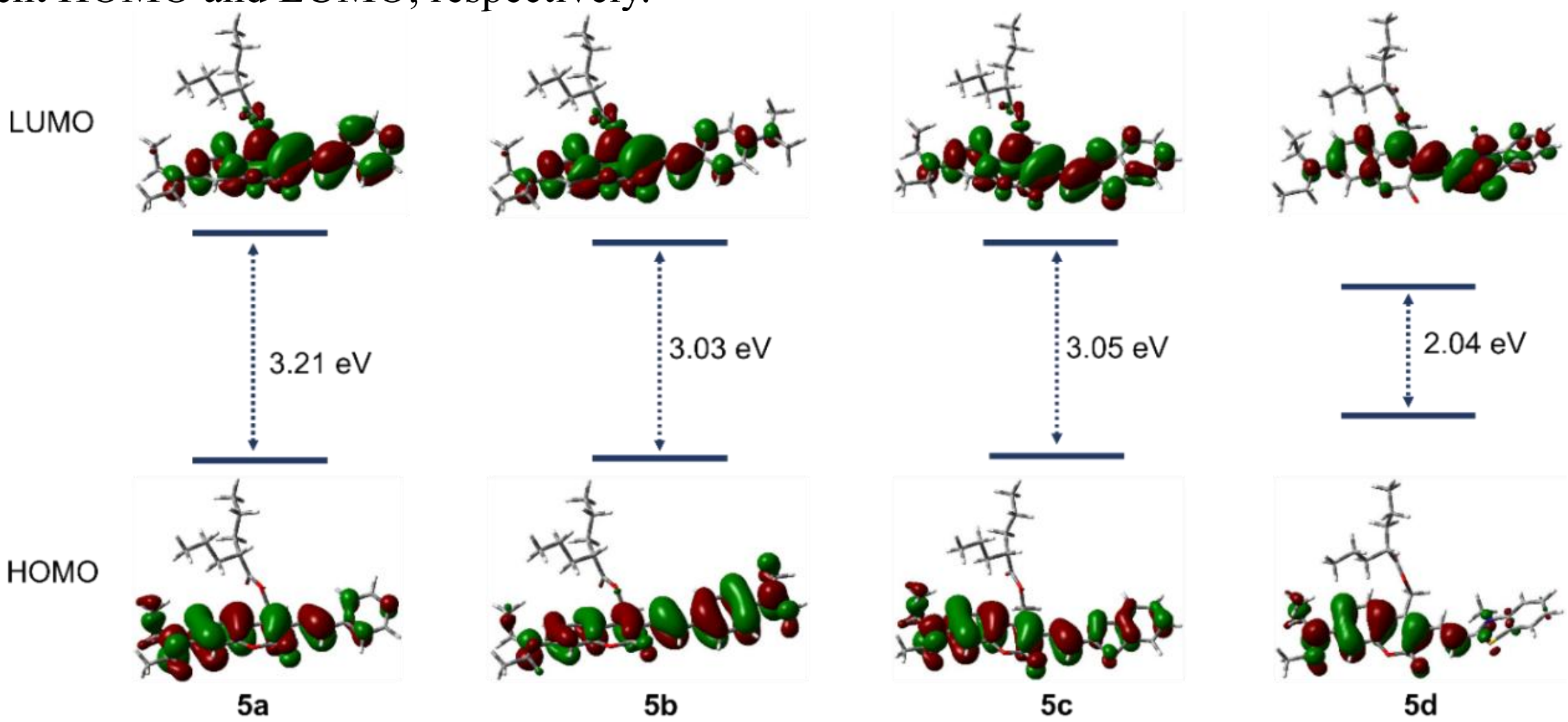

Figure S37 HOMO-LUMO electronic distribution and the energy gap for fluorogen-based PRPGs (**5a-d**).

Table S6 Summary of states of **5d** in two solvent systems

Compound 5d	Relative energy (eV)	εHOMO (a.u.)	εLUMO (a.u.)	Dipole moment (D)	ΔE (eV)	λ(nm)	f_{osc}	Major component	Dihedral angle (degrees)
Acetonitrile : water = 1 : 9									

Ground state	0	-0.256	-0.091	11.225	2.863	432.99	1.6439	H → L (44.75)	-175.897
E-form S_1	0.933	-0.257	-0.112	6.920	2.373	522.42	1.8098	H → L (46.42)	-177.733
E-form TS_1	2.840	-0.259	-0.151	7.890	1.452	853.57	0.5289	H → L (48.51)	-169.405
E-form TS_2	0.056	-0.255	-0.098	16.681	2.630	471.36	1.6972	H → L (45.71)	-177.131
Z-form S_1	0.011	-0.255	-0.078	12.671	3.072	403.62	0.7527	H → L (43.52)	7.963
Z-form TS_1	3.029	-0.260	-0.152	16.177	1.351	917.79	0.2003	H → L (48.26)	10.544
Z-form TS_2	0.068	-0.257	-0.085	14.044	2.919	424.77	0.8169	H → L (44.39)	9.183
5d(PP)		-0.255	-0.088	11.472	2.860	433.45	1.7359	H → L (44.97)	178.727
Valproic acid		-0.358	+0.042	2.414	8.252	150.24	0.0823	H → L+2 (20.31)	
Methanol : glycerol = 1 : 9									
Ground state	0.025	-0.257	-0.091	11.163	2.857	433.92	1.6446	H → L (44.75)	-175.922
E-form S_1	-0.076	-0.256	-0.094	10.740	2.324	533.49	1.8136	H → L (47.36)	-175.396
E-form TS_1	2.867	-0.260	-0.152	7.807	1.451	854.49	0.5287	H → L (48.51)	-169.405
E-form TS_2	0.087	-0.256	-0.099	16.570	2.626	472.23	1.6968	H → L (45.70)	-177.131
Z-form S_1	0.034	-0.256	-0.079	12.593	3.065	404.47	0.7522	H → L (43.52)	8.028
Z-form TS_1	3.059	-0.261	-0.153	16.080	1.350	918.26	0.2003	H → L (48.24)	10.544
Z-form TS_2	0.096	-0.257	-0.086	13.998	2.915	425.30	0.8147	H → L (44.37)	9.183
5d(PP)		-0.256	-0.089	11.381	2.855	434.27	1.7373	H → L (44.97)	178.744
Valproic acid		-0.358	+0.042	2.399	8.248	150.31	0.0823	H → L+2 (20.57)	

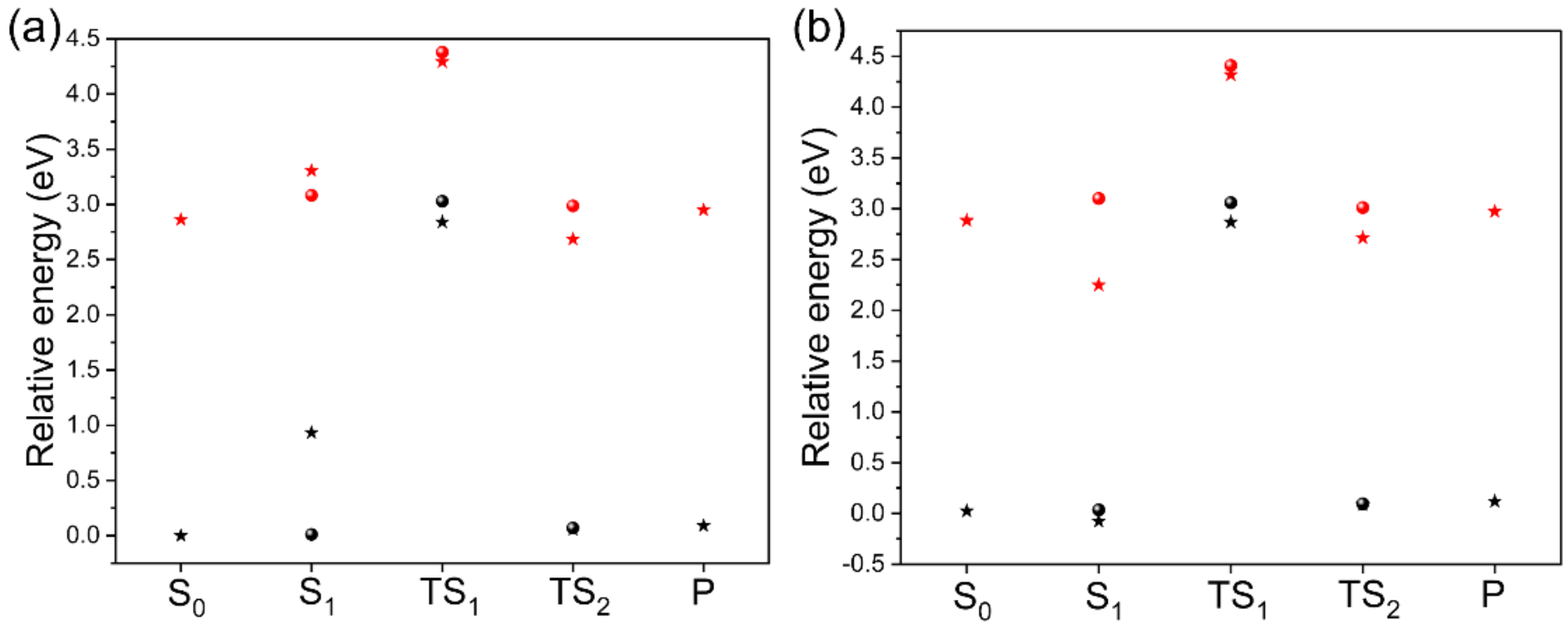

Figure S38 Energy levels of states of **5d** in (a) Acetonitrile/ water, (b) Methanol/ glycerol. Black represents the ground state, Red represents the excited state, the star and the dot represent *E* and *Z* forms, respectively.

Table S7 Total Mulliken charges on thiazole, coumarin, their connecting part, amine and the carboxylate part, in various stages of interaction of **5d**

	Thiazole	Coumarin	Connector	Amine	Carboxylate
		Acetonitrile : water = 1:9			
Ground state	0.7438	0.0753	0.1193	0.2156	-0.1541
E-form S_1	0.6234	0.0328	0.2823	0.2329	-0.1713
E-form TS_1	0.6829	0.3859	0.1984	0.2591	-0.5263
E-form TS_2	0.6979	-0.0138	0.2269	0.2263	-0.3833
Z-form S_1	0.8013	-0.0176	0.1563	0.2022	-0.1422
Z-form TS_1	0.8236	0.4253	0.0905	0.2305	-0.5698
Z-form TS_2	0.0340	0.0979	0.1450	0.1549	-0.3650
		Methanol : glycerol = 1:9			
Ground state	0.7423	0.0764	0.1189	0.2157	-0.1533
E-form S_1	0.6926	-0.0429	0.2423	0.2249	-0.1169
E-form TS_1	0.6820	0.3858	0.1975	0.2589	-0.5241
E-form TS_2	0.6965	-0.0118	0.2257	0.2260	-0.3825
Z-form S_1	0.8003	-0.0165	0.1555	0.2022	-0.1414
Z-form TS_1	0.8226	0.4236	0.0898	0.2303	-0.5663
Z-form TS_2	0.0338	0.0990	0.1448	0.1546	-0.3646

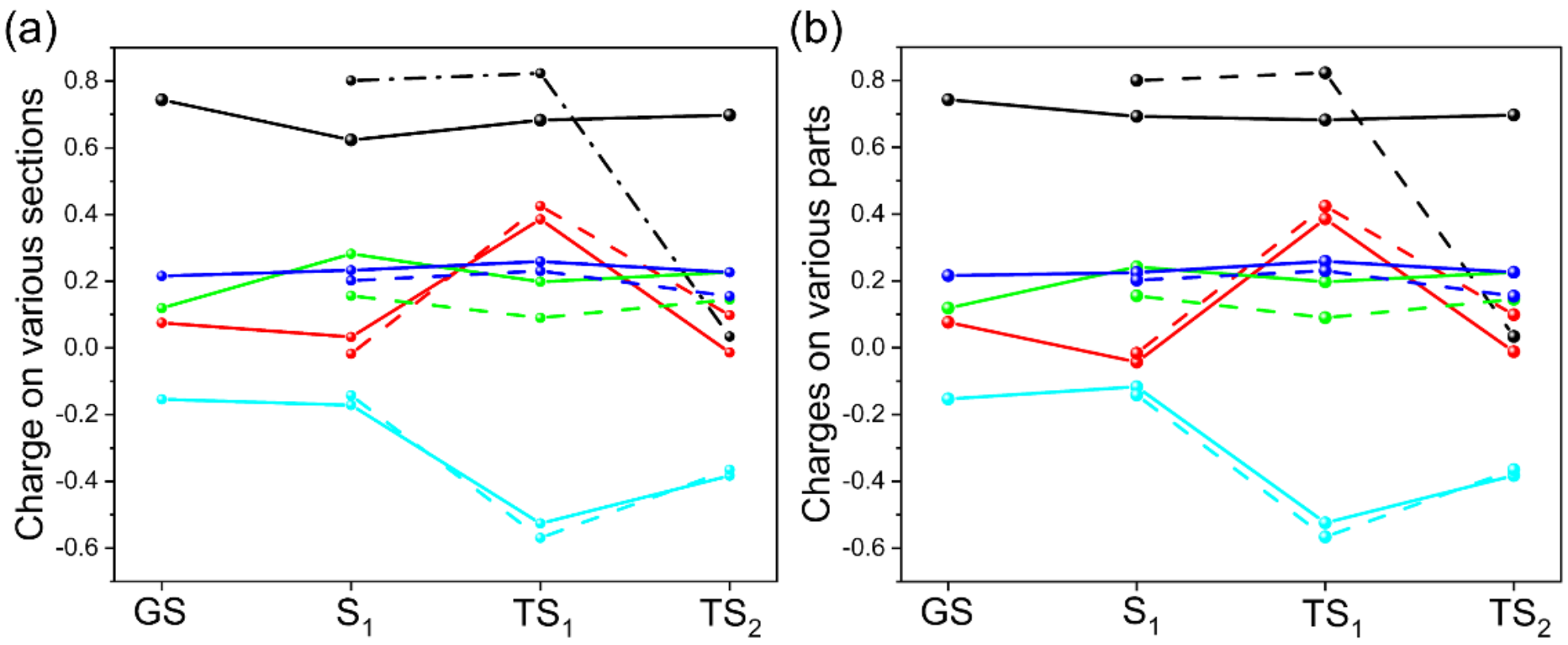

Figure S39 Charges on thiazole (black), coumarin (red), connector (green), amine (blue) and carboxylate (cyan) in (a) Acetonitrile/ water, (b) Methanol/ glycerol for **5d**. *E*-form data are shown in solid lines, and *Z*-form data in broken lines.

11. Docking study:

To explore the interaction between the protein and the compound **5d**, a docking study was undertaken between the human TAR DNA-binding protein 43 (TDP-43) and compound **5d** using AutoDock Vina.[9,10] The structure of the protein was downloaded from the Cambridge Crystallographic Data Centre (protein ID: 6T4B). There were several binding modes possible for each ligand molecule. The docking results are tabulated in **Table S8**.

Table S8 Results of docking of the ligand molecule (**5d**) with the protein (ID: 6T4B) using AutoDock Vina.

Configuration	Docking score (kcal/mole)
1	-4.471
2	-4.259
3	-4.259
4	-4.170
5	-4.081
6	-4.072
7	-4.041

8	-3.981
9	-3.945

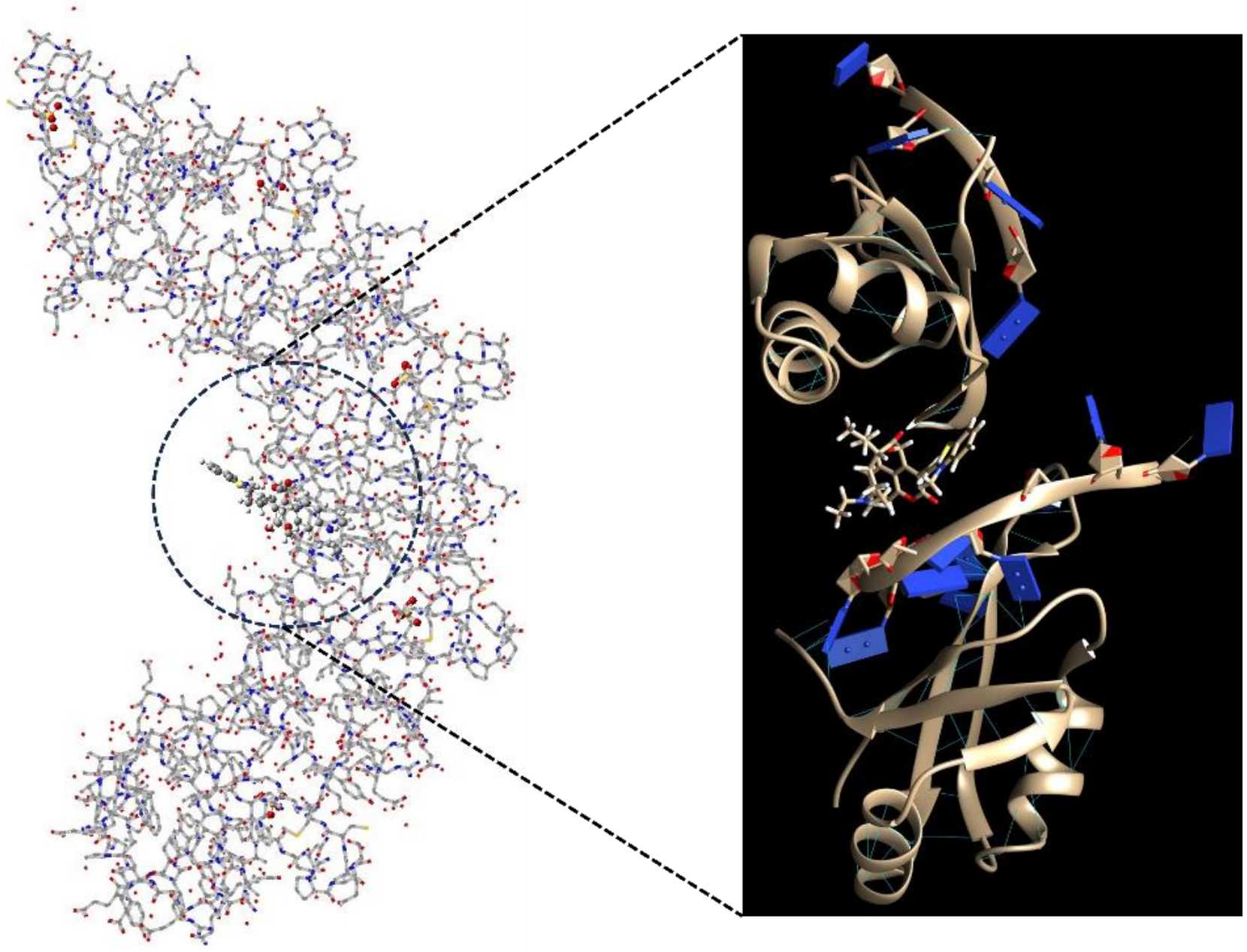

Figure S40 3D and 2D views of the dock pose of human TDP-43 and **5d**.

12. References:

1. Y. Zhao and D. G. Truhlar, *Theor. Chem. Acc.*, 2008, **120**, 215-241.

2. A. Schaefer, H. Horn and R. Ahlrichs, *J. Chem. Phys.*, 1992, **97**, 2571-2577.

3. S. Grimme, J. Antony, S. Ehrlich, H. Krieg, *J. Chem. Phys*. 2010, **132**, 154104.

4. T. Yanai, D. P. Tew and N. C. Handy, *Chem. Phys. Lett*., 2004, **393**, 51-57.

5. (a) B. Mahato, A. N. Panda, *J. Phys. Chem. A,* 2021, **125**, 115-125. (b) R. Sarkar, M. Boggio-Pasqua, P-F. Loos and D. Jacquemin, *J. Chem. Theory Comput*., 2021, **17**, 1117-1132. (c) S. Slavova and L. Antonov, *J. Phys. Chem. A*, 2024, **128**, 1280-1287.

6. (a) M. W. Wong, M. J. Frisch and K. B. Wiberg, *J. Am. Chem. Soc.*, 1991, **113**, 4776-4782. (b) M. W. Wong, K. B. Wiberg and M. J. Frisch, *J. Am. Chem. Soc.*, 1992, **114**, 523-529.

7. (a) J. Tomasi, B. Mennucci and R. Cammi, *Chem. Rev.*, 2005, **105**, 2999-3093. (b) F. Lipparini, G. Scalmani, B. Mennucci, E. Cances, M. Caricato and M. J. Frisch, *J. Chem. Phys.*, 2010, **133**, 014106.

8. A. Schaefer, H. Horn and R. Ahlrichs, *J. Chem. Phys*., 1992, **97**, 2571-2577.

9. J. Eberhardt, D. Santos-Martins, A. F. Tillack, S. Forli, AutoDock Vina 1.2.0: New Docking Methods, Expanded Force Field, and Python Bindings. *J. Chem. Inf. Model.*, 2021, **61**, 3891-3898.

10. O. Trott, A. J. Olson, AutoDock Vina: improving the speed and accuracy of docking with a new scoring function, efficient optimization and multithreading. *J. Comp. Chem.*, 2010, **31**, 455-461.

13. Optimized Cartesian coordinates from DFT calculation:

Coordinates for 5a

Ground state

C	-3.14868900	-2.24102200	-0.48029800
C	-2.04693500	-2.93569900	0.05015100
C	-0.77488500	-2.41301400	-0.07735300
O	0.23705400	-3.15483900	0.45227000
C	1.54781500	-2.77488000	0.42138000
C	1.86914700	-1.46534900	-0.16413700
C	0.86014700	-0.73264200	-0.73062500
C	-0.49650900	-1.19790500	-0.70282500

C	-1.60649700	-0.50975400	-1.23187900
C	-2.88125800	-0.99742600	-1.12252900
O	2.33741100	-3.55113500	0.89992300
C	3.24146900	-0.97036100	-0.07350900
C	1.09935500	0.60096800	-1.38996100
N	-4.41731400	-2.74604600	-0.39618000
C	-4.62115300	-3.93400100	0.44239700
C	-5.55826300	-1.86496400	-0.64121200
C	-5.81441600	-0.90053000	0.51158300
C	-6.03322500	-4.49251700	0.41746500
O	0.57562900	1.60205800	-0.50429500
C	-0.12475300	2.62000200	-1.03781400
C	-0.76685500	3.46065100	0.03717900
C	-1.07879100	4.87874000	-0.45757800
C	-2.01842400	2.67985000	0.49318100
O	-0.26198500	2.76746100	-2.22605800
C	-2.79409100	3.35128900	1.62053300
C	-0.83101300	5.95049200	0.60262100
C	-1.23661500	7.33639900	0.11491800
C	-3.97814700	2.49796000	2.06251100
C	4.35917200	-1.67176200	0.15828900
C	5.69592900	-1.07079600	0.24241100
C	6.82081800	-1.89172400	0.14275400
C	5.92871500	0.29553700	0.42389300
C	7.23229900	0.75979600	0.47198200
C	8.08681200	-1.32967400	0.20635300
N	8.30814300	-0.02460900	0.36494500
H	-1.46803700	0.43691800	-1.73897200
H	-3.68530200	-0.40867200	-1.53486300
H	-5.39639200	-1.32687400	-1.57288700
H	-6.43149400	-2.48668200	-0.81562000
H	-4.94206500	-0.26956100	0.68909600
H	-6.66600800	-0.25622800	0.29020500
H	-6.02915600	-1.45071900	1.42965400
H	-3.94624500	-4.71172200	0.08227200
H	-4.33178100	-3.70996000	1.47712600
H	-6.04118500	-5.41821600	0.99232600
H	-6.75828200	-3.81602000	0.86945000
H	-6.35199300	-4.72406100	-0.59935000
H	-3.64237200	1.51878000	2.41093400
H	-4.53135600	2.97357400	2.87289600
H	-4.66882300	2.33775800	1.23176400
H	-2.30386800	7.36804000	-0.11483000
H	-1.03134800	8.09985600	0.86604200
H	-0.69253500	7.60219700	-0.79397100
H	-1.37535700	5.70760500	1.51794900
H	-2.12337000	3.52731800	2.46662300
H	-0.46561400	5.10564000	-1.33247500
H	-2.11907300	4.92469400	-0.79576700
H	-2.68036900	2.55007100	-0.37131000
H	-1.71503900	1.67888100	0.81470700
H	-3.15360900	4.32967700	1.29221100
H	0.23091400	5.94523400	0.86479400
H	-0.06755000	3.50199700	0.87726600
H	2.14824800	0.81018800	-1.57121000
H	0.58158600	0.64796000	-2.34668700
H	-2.14643600	-3.87532900	0.56929300
H	3.34501000	0.09903200	-0.20676900
H	4.31771100	-2.74603400	0.26997500
H	5.11505400	0.99817500	0.54433900
H	7.42358200	1.81790700	0.61202500
H	6.70965200	-2.96084400	0.01250600
H	8.96526500	-1.96033200	0.12589600

Excited state

C	-3.51914900	-1.79284700	-0.44980400
C	-2.46388900	-2.67255000	-0.05270700
C	-1.15751900	-2.37395200	-0.35398500
O	-0.19659400	-3.29267600	0.04602400
C	1.18223700	-3.02462200	0.00594500
C	1.60229600	-1.74694000	-0.55064800
C	0.61887600	-0.92588900	-1.24045400
C	-0.75338900	-1.19633400	-1.05996800
C	-1.82970600	-0.34698000	-1.49316800
C	-3.14343100	-0.62464700	-1.20177600
O	1.90005000	-3.91604300	0.47584000
C	2.93688800	-1.30126900	-0.38090200
C	1.03997000	0.29157000	-1.98680200
N	-4.81702900	-2.04857500	-0.11466000

C	-5.11991000	-3.23350700	0.72152200
C	-5.87676000	-1.07566600	-0.42702300
C	-5.94453000	0.06871700	0.59001100
C	-6.59447600	-3.47084100	1.02296800
O	1.00608000	1.45108600	-1.05017900
C	0.16982600	2.49762600	-1.30000800
C	-0.07951300	3.32160400	-0.06370000
C	-0.56103900	4.74497300	-0.40263600
C	-1.09091200	2.50477600	0.78796200
O	-0.37756600	2.66313500	-2.39689400
C	-1.44966200	3.12188500	2.14239900
C	0.01409600	5.82515800	0.52435900
C	-0.52399300	7.22142200	0.19893600
C	-2.39608800	2.21748000	2.94000600
C	4.02324000	-1.98190000	0.15749900
C	5.33920200	-1.42124300	0.32835700
C	6.37548900	-2.22299900	0.87984500
C	5.70099300	-0.08985700	-0.01796200
C	7.00435900	0.35000600	0.19132000
C	7.65133900	-1.69616200	1.05358200
N	7.97713100	-0.42756500	0.71811000
H	-1.60638400	0.56429500	-2.04965500
H	-3.90121600	0.07717100	-1.54241300
H	-5.71707800	-0.68655500	-1.44375300
H	-6.83515800	-1.60674400	-0.46336700
H	-4.98752700	0.61819600	0.63874200
H	-6.73870200	0.78147600	0.30734700
H	-6.16961100	-0.31550600	1.60015300
H	-4.71661900	-4.12515400	0.20973400
H	-4.56650000	-3.13560900	1.67492800
H	-6.67290700	-4.36812300	1.66003600
H	-7.05391900	-2.63361200	1.57439400
H	-7.18247800	-3.66279000	0.10972800
H	-1.92908300	1.23571200	3.14226700
H	-2.66717900	2.67072600	3.90992700
H	-3.33162000	2.03226700	2.37872800
H	-1.62284900	7.26010300	0.31915100
H	-0.08479200	7.98918000	0.86058800
H	-0.29207300	7.50164100	-0.84564000
H	-0.20976800	5.57704100	1.57757300
H	-0.52434900	3.30316100	2.72161000
H	-0.28552200	4.98904300	-1.44403400
H	-1.66719300	4.77744200	-0.37054600
H	-2.01686000	2.37141500	0.19496700
H	-0.67242800	1.49460300	0.95189100
H	-1.92513100	4.10816100	1.99246600
H	1.11765100	5.82019300	0.43612500
H	0.87255600	3.36679300	0.49378300
H	2.06845900	0.22944600	-2.36267000
H	0.37657600	0.52710000	-2.82997800
H	-2.64792100	-3.59144900	0.49646200
H	3.11730500	-0.27078300	-0.69768100
H	3.88907500	-3.01770500	0.47180300
H	4.97274300	0.60040000	-0.44723700
H	7.28709300	1.37313600	-0.07354500
H	6.16788900	-3.25647900	1.16823600
H	8.44997100	-2.31130900	1.47838300

Coordinates for 5b

Ground state

C	-4.09687200	-2.19614500	-0.44881200
C	-2.99708300	-2.90832500	0.05845300
C	-1.71816800	-2.40538500	-0.09371700
O	-0.70695000	-3.16311100	0.41680600
C	0.60843400	-2.80017300	0.36920900
C	0.94373400	-1.49791600	-0.23075600
C	-0.06485700	-0.75481100	-0.77955000
C	-1.43269000	-1.19467700	-0.71984300
C	-2.54061500	-0.48778000	-1.22379700
C	-3.82230900	-0.95613600	-1.09036100
O	1.39119800	-3.58490100	0.84591000
C	2.32672100	-1.02445400	-0.16720300
C	0.18693000	0.56894300	-1.45017600
N	-5.37602900	-2.68420300	-0.34472000
C	-5.57864200	-3.86639800	0.50082100
C	-6.50218800	-1.76866600	-0.52307400
C	-6.69049200	-0.82674300	0.66121000
C	-6.99764400	-4.40840900	0.49866900

O	-0.28590900	1.58939100	-0.55479400
C	-0.97939800	2.61919400	-1.07067400
C	-1.57868800	3.47227600	0.01966200
C	-1.87633900	4.89594400	-0.46747000
C	-2.83284600	2.71478400	0.50673800
O	-1.14504700	2.77076000	-2.25525900
C	-3.56874800	3.39935600	1.65258300
C	-1.58534300	5.96254900	0.58694800
C	-1.97545500	7.35607300	0.10837700
C	-4.75416100	2.56473400	2.12572100
C	3.43941500	-1.76188700	-0.02680700
C	4.80437900	-1.24135100	0.04063700
C	5.87511600	-2.14091800	0.05881600
C	5.12571100	0.12106300	0.09509400
C	6.43289100	0.56087200	0.14669800
C	7.19256400	-1.72276200	0.10989000
C	7.51219600	-0.35088700	0.14002600
N	8.81494700	0.08394300	0.15829000
C	9.87410100	-0.88044500	0.39163200
C	9.08920600	1.48168400	0.43812600
H	-2.39687800	0.45917200	-1.72922400
H	-4.62438400	-0.35299600	-1.48557300
H	-6.36314400	-1.21107100	-1.44690600
H	-7.39725400	-2.36409800	-0.67884200
H	-5.79741400	-0.21964700	0.81799100
H	-7.53503600	-0.15837400	0.48957500
H	-6.88153100	-1.39390400	1.57430600
H	-4.91961800	-4.65278900	0.13051000
H	-5.26946000	-3.64623600	1.53118200
H	-7.00650700	-5.34068300	1.06285900
H	-7.70563700	-3.72854200	0.97221800
H	-7.33883000	-4.62323100	-0.51468000
H	-4.42410500	1.58203200	2.46961800
H	-5.28121800	3.05080500	2.94727300
H	-5.46656500	2.41138900	1.31216900
H	-3.04662500	7.40769400	-0.09836700
H	-1.73995500	8.11515000	0.85508300
H	-1.44618900	7.61231000	-0.81194000
H	-2.11395000	5.72955800	1.51398900
H	-2.87408100	3.56558200	2.48115000
H	-1.27926200	5.11227600	-1.35610000
H	-2.92309100	4.96091900	-0.78188400
H	-3.51804500	2.59728900	-0.34123500
H	-2.53984700	1.70826600	0.82037600
H	-3.92119800	4.38298900	1.33229100
H	-0.51822200	5.93725400	0.82588300
H	-0.85818800	3.50056300	0.84221700
H	1.23682000	0.74799800	-1.65791100
H	-0.35623400	0.62911500	-2.39196800
H	-3.10085100	-3.84704800	0.57859000
H	2.43769000	0.05032700	-0.23968900
H	3.35448100	-2.83903400	0.03483800
H	4.33977000	0.86644800	0.11107800
H	6.61816600	1.62374600	0.19843400
H	5.66714000	-3.20513300	0.03419000
H	7.97374700	-2.46843100	0.12790400
H	10.83015200	-0.36461000	0.37373800
H	9.76737000	-1.38771600	1.35782000
H	9.88944000	-1.63780100	-0.39375600
H	10.16381300	1.64001400	0.41536600
H	8.64214500	2.12547500	-0.32093900
H	8.71065400	1.79044600	1.41965100

Excited state

C	-4.45427100	-1.75336700	-0.40291900
C	-3.39995300	-2.61690100	0.01993600
C	-2.08675100	-2.32051500	-0.28432200
O	-1.12416400	-3.22803300	0.14491100
C	0.25881500	-2.98084600	0.06835000
C	0.68595800	-1.74967100	-0.56685400
C	-0.30522200	-0.91984300	-1.22982300
C	-1.68272700	-1.16354700	-1.01546700
C	-2.75519500	-0.31418100	-1.44452700
C	-4.07765100	-0.58958600	-1.15021000
O	0.97176400	-3.85967600	0.57828600
C	2.04646600	-1.35264700	-0.49644000
C	0.10873300	0.27315100	-2.01749800
N	-5.76486200	-2.03031500	-0.09459500
C	-6.05564600	-3.20365000	0.75089200

C	-6.81923100	-1.06094700	-0.41358400
C	-6.89068300	0.09202400	0.59574000
C	-7.53563300	-3.47266400	1.00270400
O	0.16723800	1.45551900	-1.11071900
C	-0.67496200	2.50498900	-1.31247100
C	-0.82997100	3.34505800	-0.06983200
C	-1.35152400	4.75916500	-0.38366200
C	-1.76712100	2.53475500	0.86786500
O	-1.29311400	2.67189900	-2.37075600
C	-2.02910500	3.18805000	2.22864900
C	-0.71460400	5.84923400	0.49136800
C	-1.30662500	7.23492100	0.21464300
C	-2.90345400	2.29800000	3.12039800
C	3.14701900	-2.04396400	0.02606200
C	4.47562100	-1.52748200	0.08771800
C	5.52103300	-2.34467200	0.62473200
C	4.85893500	-0.22097000	-0.35693300
C	6.16335500	0.22611900	-0.27425300
C	6.82888100	-1.91048400	0.71500500
C	7.19920300	-0.60261900	0.26557300
N	8.48825000	-0.16217300	0.34829700
C	9.52766200	-1.02636300	0.90612100
C	8.84144500	1.17770300	-0.11890000
H	-2.52987300	0.59845200	-2.00196500
H	-4.83400300	0.11585400	-1.48984600
H	-6.65423300	-0.67769300	-1.43274600
H	-7.77960600	-1.59087700	-0.44940300
H	-5.92981000	0.63410000	0.64579000
H	-7.67902200	0.80795400	0.30778500
H	-7.12122300	-0.29060600	1.60521400
H	-5.61426300	-4.09395400	0.26809800
H	-5.53650700	-3.08221200	1.72231200
H	-7.61997700	-4.36560500	1.64344300
H	-8.03053700	-2.63826800	1.52647500
H	-8.08100500	-3.67964900	0.06716300
H	-2.41208200	1.32638600	3.30701000
H	-3.10015200	2.77422200	4.09585000
H	-3.87654400	2.09566500	2.63701200
H	-2.38875100	7.25096200	0.43762400
H	-0.82162600	8.01186900	0.82982700
H	-1.17776000	7.51147900	-0.84737600
H	-0.84016200	5.59629800	1.55964000
H	-1.06398500	3.39222700	2.72950200
H	-1.15731000	4.99766900	-1.44481400
H	-2.45265300	4.77994400	-0.26426300
H	-2.73314600	2.38444500	0.34514000
H	-1.32630700	1.53171500	1.02299500
H	-2.52549100	4.16521500	2.08468500
H	0.37557200	5.86247900	0.30319400
H	0.16376400	3.40406700	0.41006900
H	1.11231800	0.17363900	-2.44995900
H	-0.59840700	0.51344600	-2.82388300
H	-3.58457800	-3.52450700	0.58938300
H	2.24856100	-0.34963400	-0.88421800
H	2.99504400	-3.05357400	0.41065400
H	4.10914100	0.45380500	-0.77490000
H	6.39623500	1.22973600	-0.62830400
H	5.26681500	-3.34885300	0.97458400
H	7.57930400	-2.57952400	1.13434800
H	10.48717900	-0.49381500	0.88570600
H	9.30269300	-1.29920500	1.95279000
H	9.63282900	-1.95681100	0.31984500
H	9.91693100	1.33832000	0.02994200
H	8.61635200	1.29951900	-1.19358900
H	8.29199100	1.95555800	0.44112100

Coordinates for 5c

Ground state

C	-4.13996100	-2.08923300	-0.47657000
C	-3.06379900	-2.85175100	0.01336400
C	-1.76918200	-2.40058800	-0.14671700
O	-0.78688100	-3.20501300	0.34463300
C	0.54091100	-2.89741000	0.28033500
C	0.91918200	-1.59857000	-0.29335400
C	-0.06349400	-0.80323200	-0.82846700
C	-1.43950000	-1.19638800	-0.77146200
C	-2.52509500	-0.44031200	-1.26025100
C	-3.82081200	-0.85588200	-1.11715300
O	1.30036300	-3.72563300	0.71917900

C	2.31294800	-1.18403600	-0.23516800
C	0.22513200	0.52869700	-1.47244900
N	-5.43033200	-2.52022300	-0.35607700
C	-5.67849900	-3.70646600	0.47378200
C	-6.52770900	-1.57855500	-0.57444600
C	-6.70096000	-0.60122400	0.58284300
C	-7.12125300	-4.18016200	0.48862100
O	-0.22134300	1.53616600	-0.55291300
C	-0.88040500	2.60064300	-1.04727900
C	-1.44343400	3.45415100	0.06111400
C	-1.68849400	4.89703200	-0.39855800
C	-2.72386500	2.73651400	0.54042100
O	-1.04347700	2.77802000	-2.22808900
C	-3.42339100	3.42761800	1.70503100
C	-1.34603600	5.93210000	0.67163200
C	-1.68562500	7.34813600	0.22154400
C	-4.64253000	2.63700400	2.16775400
C	3.38818200	-1.90353000	0.12223200
C	4.71230300	-1.29557600	0.14558400
S	6.10053800	-2.30796200	0.56314300
N	4.98718800	-0.06033400	-0.11669100
C	6.34056700	0.18926000	-0.00804700
C	7.12965600	-0.91709700	0.35661400
C	6.95065900	1.42782000	-0.23017800
C	8.50973800	-0.81107200	0.50605900
C	8.31988300	1.53374200	-0.08321900
C	9.09426000	0.42317800	0.28179100
H	-2.34858000	0.50248200	-1.76257100
H	-4.60189000	-0.21815700	-1.49955200
H	-6.35998400	-1.05081600	-1.51104500
H	-7.43760900	-2.15174700	-0.72517200
H	-5.79122700	-0.01882800	0.73688200
H	-7.52152100	0.08836500	0.38180900
H	-6.92175700	-1.13844300	1.50707500
H	-5.06269700	-4.51770900	0.08271700
H	-5.34347300	-3.51345900	1.50069900
H	-7.16577600	-5.10896800	1.05675200
H	-7.79088000	-3.46642100	0.96800500
H	-7.48444600	-4.38379200	-0.51917400
H	-4.35631800	1.63132500	2.48343800
H	-5.13932400	3.12581200	3.00635400
H	-5.36812100	2.53867000	1.35740700
H	-2.75576500	7.44504200	0.02605600
H	-1.41401500	8.08386400	0.97926800
H	-1.15494000	7.60013700	-0.69911800
H	-1.87486400	5.70304700	1.59953400
H	-2.71754000	3.54501900	2.53246700
H	-1.09107500	5.10538700	-1.28879000
H	-2.73456600	5.00879800	-0.70180100
H	-3.41825700	2.66413900	-0.30510300
H	-2.46968200	1.71291800	0.83216100
H	-3.73357900	4.43255700	1.40805300
H	-0.27862800	5.86070400	0.89948100
H	-0.71907800	3.43968800	0.88059900
H	1.27631600	0.69103900	-1.68283300
H	-0.31813600	0.61900000	-2.41162400
H	-3.20129100	-3.78986300	0.52637500
H	2.51056500	-0.15093600	-0.49082100
H	3.31563200	-2.94338900	0.40104200
H	6.34282200	2.27817400	-0.51095100
H	9.10753500	-1.66765500	0.78770400
H	10.16547200	0.53154500	0.39097000
H	8.80452400	2.48654800	-0.25210400

Excited state

C	-4.33565700	-1.79522900	-0.45273700
C	-3.28011100	-2.66869800	-0.04669400
C	-1.97365200	-2.36313000	-0.34196100
O	-1.00935500	-3.26998000	0.07505500
C	0.36324400	-3.02996200	-0.01078400
C	0.79400800	-1.74667300	-0.57564800
C	-0.19808500	-0.90951000	-1.22073700
C	-1.56918300	-1.18870400	-1.04847200
C	-2.64576600	-0.34551000	-1.49027800
C	-3.95930400	-0.62693600	-1.20502300
O	1.08587700	-3.93156800	0.42082600
C	2.13596500	-1.34431300	-0.45429000
C	0.20895100	0.33653500	-1.93518300
N	-5.63423200	-2.05580100	-0.12794400

C	-5.93793100	-3.24106000	0.70779400
C	-6.69638200	-1.08869800	-0.45124900
C	-6.77831300	0.05748300	0.56226300
C	-7.41384400	-3.48567200	0.99659900
O	0.10781400	1.46649700	-0.97868100
C	-0.72080300	2.51464800	-1.25153800
C	-1.01605500	3.32559700	-0.01691800
C	-1.49671400	4.74855100	-0.35908400
C	-2.04652000	2.49591300	0.79850500
O	-1.22352400	2.68903100	-2.36760900
C	-2.44584400	3.10038800	2.14730200
C	-0.95434800	5.82505800	0.59167800
C	-1.49215200	7.22042600	0.26203700
C	-3.40611500	2.18335800	2.91322500
C	3.22544000	-2.05126100	0.07938000
C	4.50148000	-1.46187200	0.14901200
S	5.92950700	-2.40583000	0.85173900
N	4.83375300	-0.23732400	-0.24914100
C	6.16008300	0.06554700	-0.05280600
C	6.94746100	-0.96621200	0.53516300
C	6.77353000	1.29312300	-0.38449600
C	8.30548500	-0.78798600	0.78857800
C	8.13020800	1.46810000	-0.13038400
C	8.89610400	0.43606100	0.45289300
H	-2.42502700	0.56323200	-2.05155700
H	-4.71771200	0.07079100	-1.55235800
H	-6.53086200	-0.70134100	-1.46768400
H	-7.65164800	-1.62486700	-0.49428400
H	-5.82516200	0.61309500	0.61627400
H	-7.57485800	0.76482600	0.27277500
H	-7.00764000	-0.32576400	1.57183800
H	-5.52575700	-4.13141800	0.20090600
H	-5.39350100	-3.13865700	1.66584400
H	-7.49337900	-4.38234200	1.63439200
H	-7.88256200	-2.64993600	1.54247500
H	-7.99262800	-3.68205100	0.07841500
H	-2.93745400	1.20338500	3.12028400
H	-3.70558500	2.62733000	3.87905600
H	-4.32539100	1.99581800	2.32658800
H	-2.59413500	7.25143000	0.35207600
H	-1.07628800	7.98568100	0.94142300
H	-1.23319600	7.51016700	-0.77354100
H	-1.20567600	5.56741700	1.63636800
H	-1.53748100	3.28392900	2.75205600
H	-1.19355300	5.00214200	-1.39043900
H	-2.60351400	4.77417400	-0.35765100
H	-2.95469700	2.36213100	0.17877400
H	-1.62703400	1.48690400	0.96630500
H	-2.92437300	4.08433600	1.99197400
H	0.15121300	5.82755000	0.53401100
H	-0.08167400	3.37292300	0.56959000
H	1.24710900	0.31119800	-2.28563000
H	-0.43718300	0.56328700	-2.79418300
H	-3.46227000	-3.58577100	0.50597900
H	2.38177100	-0.33673300	-0.79835600
H	3.09325600	-3.07028700	0.44119600
H	6.16789100	2.08171700	-0.83466700
H	8.90183100	-1.58451300	1.23973200
H	9.96030000	0.59132600	0.64504700
H	8.61052900	2.41603600	-0.38486900

Coordinates for 5d in Acetonitrile/ water

Ground state

C	-4.30668300	-2.03423400	-0.46131500
C	-3.23094500	-2.81981100	0.00202200
C	-1.93630900	-2.38966900	-0.18152900
O	-0.95817400	-3.21187800	0.28450400
C	0.37123100	-2.92103900	0.20353200
C	0.75424800	-1.62457900	-0.36577000
C	-0.22946200	-0.80796000	-0.88825400
C	-1.59861000	-1.18561800	-0.81405300
C	-2.68543800	-0.40737200	-1.27662200
C	-3.98082000	-0.80119900	-1.10811300
O	1.12824600	-3.76231900	0.62115000
C	2.14373900	-1.22774600	-0.32723800
C	0.06177600	0.53073000	-1.52148700
N	-5.59269100	-2.43851000	-0.30861200
C	-5.84943800	-3.63775100	0.50346900
C	-6.68903800	-1.50352700	-0.56383900

C	-6.86674800	-0.49435500	0.56464100
C	-7.30169600	-4.07736300	0.55183900
O	-0.34553000	1.52426900	-0.57294100
C	-1.00104900	2.60850300	-1.03397400
C	-1.52616200	3.44846600	0.10208800
C	-1.75595300	4.90439200	-0.32345200
C	-2.80873900	2.74216700	0.59318700
O	-1.18638100	2.80642700	-2.20752700
C	-3.46957900	3.41920700	1.78823800
C	-1.37138500	5.91086400	0.75967200
C	-1.69794600	7.34137000	0.34743300
C	-4.69515800	2.64274800	2.25794500
C	3.19155600	-1.90821400	0.18308600
C	4.52192800	-1.35622500	0.23349100
S	5.52934400	-1.69568900	1.59312800
N	5.12150500	-0.58144000	-0.66211200
C	6.40592000	-0.16610900	-0.29282800
C	6.80196500	-0.70388500	0.92985300
C	7.24669500	0.67982400	-1.01171900
C	8.05514900	-0.42962100	1.46653100
C	8.48943300	0.95506300	-0.47351200
C	8.89153100	0.40606000	0.74995200
C	4.58767600	-0.24736300	-1.98663500
H	-2.50484100	0.53457900	-1.77864900
H	-4.76013500	-0.14951000	-1.46945800
H	-6.51318100	-1.00424900	-1.51439500
H	-7.59784100	-2.08063300	-0.70256100
H	-5.95743700	0.09127000	0.70781700
H	-7.68545200	0.18933000	0.33830300
H	-7.09342000	-1.00542200	1.50192600
H	-5.26231800	-4.45496100	0.08165600
H	-5.48258200	-3.46730700	1.52239200
H	-7.35195800	-5.00248200	1.12536600
H	-7.94347700	-3.34700500	1.04394500
H	-7.69324200	-4.27882700	-0.44544300
H	-4.42486800	1.62233000	2.53853700
H	-5.16063500	3.11867200	3.12153000
H	-5.44168400	2.58402500	1.46300900
H	-2.77064600	7.45950000	0.17976000
H	-1.39642700	8.05623600	1.11374200
H	-1.18492900	7.60438500	-0.58013400
H	-1.88211400	5.67054900	1.69481600
H	-2.74434000	3.50256300	2.60291700
H	-1.17450000	5.12078500	-1.22230700
H	-2.80630800	5.04007700	-0.60066700
H	-3.52242000	2.70135900	-0.23815100
H	-2.56676200	1.70801300	0.85704800
H	-3.76518900	4.43688400	1.52146100
H	-0.30028000	5.81736200	0.96051500
H	-0.78551200	3.40506900	0.90580400
H	1.10654100	0.68782500	-1.76469600
H	-0.50544600	0.64025200	-2.44386300
H	-3.37485800	-3.75791400	0.51294200
H	2.34423000	-0.23479400	-0.70279100
H	3.06022400	-2.86222100	0.67054900
H	6.93891500	1.11844000	-1.95040000
H	8.35903000	-0.85246400	2.41416400
H	9.87138000	0.64047700	1.14332100
H	9.16151400	1.61221000	-1.00835200
H	5.40374900	-0.30975500	-2.70106800
H	3.82481400	-0.97073800	-2.25430500
H	4.17576600	0.76026500	-1.98057600

Excited (S_1) state

C	5.30624300	-1.21728500	0.25402300
C	4.32083000	-2.17015200	-0.15055500
C	2.97833700	-1.93287500	0.10840500
O	2.08150700	-2.90871800	-0.31350900
C	0.69302900	-2.80076700	-0.18498100
C	0.16108100	-1.58370500	0.46429300
C	1.07560500	-0.64498000	1.01946700
C	2.49169500	-0.77470700	0.77527600
C	3.48514900	0.18615400	1.14823800
C	4.83224500	-0.01878200	0.90025500
O	0.03882900	-3.75568600	-0.62590700
C	-1.26061900	-1.35766800	0.53202500
C	0.55723300	0.47653000	1.88156300
N	6.64620400	-1.43826600	0.02313500
C	7.05908300	-2.67939500	-0.68982400

C	7.65678000	-0.41886200	0.37748300
C	7.83380700	0.66915600	-0.70060900
C	8.56436100	-2.91691800	-0.81705800
O	-0.11843100	1.53261200	1.07683200
C	0.51771400	2.74025200	0.87297600
C	-0.30629300	3.65998100	-0.00682400
C	0.07892300	5.15105500	0.19674200
C	-0.16675700	3.17001900	-1.48482700
O	1.63617500	2.98457300	1.34845300
C	-1.08848200	3.89585900	-2.48313100
C	-1.13420200	6.09354300	0.34489000
C	-0.72624300	7.56563200	0.52595900
C	-0.96124700	3.33682600	-3.91175900
C	-2.27037500	-2.23770100	0.12101700
C	-3.66777200	-2.05467400	0.12312300
S	-4.69410600	-3.25150900	-0.78564800
N	-4.45641700	-1.07582700	0.69725900
C	-5.82671700	-1.15436100	0.39373100
C	-6.16381000	-2.28159800	-0.39709700
C	-6.83806900	-0.25469300	0.79255100
C	-7.48004200	-2.53987600	-0.78434000
C	-8.15849400	-0.51238800	0.40094600
C	-8.48406100	-1.64508100	-0.37783800
C	-3.93422400	-0.07188600	1.64226200
H	3.17769500	1.12999900	1.60194500
H	5.52930500	0.76171000	1.19909600
H	7.37954600	0.03471800	1.34218300
H	8.61156700	-0.93312800	0.54971400
H	6.89237600	1.22036100	-0.87766100
H	8.60115300	1.39311900	-0.36954200
H	8.16412100	0.23247000	-1.66043600
H	6.60995700	-3.53749600	-0.15594900
H	6.60494200	-2.66243600	-1.70120300
H	8.71051500	-3.86343900	-1.36814900
H	9.07749500	-2.12278100	-1.38706100
H	9.05749300	-3.02959900	0.16499900
H	-1.22358500	2.26124000	-3.94645900
H	-1.63225000	3.87028100	-4.61128500
H	0.07378300	3.44180000	-4.29212200
H	-0.15274900	7.93287400	-0.34787600
H	-1.61240600	8.21762700	0.64436700
H	-0.08904900	7.69656100	1.42277300
H	-1.79444600	5.99943100	-0.53941900
H	-2.13919000	3.80543800	-2.14090500
H	0.71387500	5.24651200	1.09686200
H	0.70848000	5.48962100	-0.65038100
H	0.88988000	3.29320100	-1.79807100
H	-0.38701100	2.08570300	-1.51896100
H	-0.85551300	4.97803500	-2.49443200
H	-1.73867600	5.76673900	1.21547200
H	-1.36076700	3.50463800	0.28989000
H	-0.22409800	0.11622700	2.56673600
H	1.35105600	0.95125500	2.47014400
H	4.57449300	-3.09365500	-0.66453500
H	-1.54104700	-0.36846400	0.88682800
H	-1.94329200	-3.18667000	-0.31111100
H	-6.60869900	0.63393600	1.38177000
H	-7.72510300	-3.41570700	-1.39026000
H	-9.52098400	-1.82707900	-0.67110000
H	-8.94713200	0.18177100	0.70258700
H	-4.75536900	0.28570000	2.27812100
H	-3.18147200	-0.53862000	2.29425500
H	-3.49058300	0.78873100	1.11152800

First transition state (TS_1)

C	4.15018700	-1.76613600	0.64490200
C	3.10048200	-2.32977800	-0.13437000
C	1.78760400	-2.02946600	0.16590500
O	0.81942300	-2.60807200	-0.63284600
C	-0.54135700	-2.38151600	-0.51644400
C	-0.96735100	-1.37770000	0.46822100
C	0.00214700	-0.82469300	1.33899700
C	1.39286600	-1.16436200	1.21725500
C	2.44875600	-0.65978000	2.03207600
C	3.76804300	-0.94342100	1.76315900
O	-1.27285400	-3.02916500	-1.26623500
C	-2.34274700	-0.94226500	0.47828000
C	-0.37174600	0.16701700	2.31479000
N	5.45432500	-1.99180300	0.33221800

C	5.76342100	-2.81508000	-0.85900300
C	6.52907700	-1.31153400	1.07355000
C	6.73919200	0.13211100	0.60504900
C	7.24736300	-3.01832900	-1.14174900
O	-0.10616600	1.87272700	0.60065200
C	1.09481600	2.31479900	0.88288100
C	1.90445200	2.77949800	-0.33131700
C	2.88708500	3.91087900	0.02400000
C	2.60548400	1.49674200	-0.84618000
O	1.62340400	2.27703500	2.02371400
C	3.49174500	1.67548700	-2.08327800
C	3.05981800	4.94316000	-1.10039800
C	4.10550000	6.00888000	-0.75559900
C	4.12837100	0.34410900	-2.50214600
C	-3.35069500	-1.38060600	-0.33883200
C	-4.69086300	-0.86349900	-0.32984400
S	-5.62101800	-0.87232400	-1.84332000
N	-5.39129800	-0.36174000	0.69519900
C	-6.68273800	0.10904000	0.35936400
C	-6.99762600	-0.09953900	-0.99135500
C	-7.60172000	0.71976900	1.22472000
C	-8.23224200	0.27927700	-1.51662400
C	-8.83651900	1.10274300	0.70005900
C	-9.15260600	0.88286500	-0.65574700
C	-4.95126100	-0.37530700	2.10148800
H	2.23110400	0.00183900	2.86864000
H	4.52634500	-0.50386100	2.40689700
H	6.29922000	-1.34868400	2.14903600
H	7.45244000	-1.88981800	0.94899800
H	5.80952900	0.72156000	0.70211400
H	7.52283100	0.62062600	1.20801700
H	7.05109400	0.15596600	-0.45323800
H	5.28511900	-3.80174500	-0.72638600
H	5.28744700	-2.34393500	-1.73964200
H	7.34104500	-3.61132300	-2.06595800
H	7.77646700	-2.06455200	-1.30209800
H	7.75127200	-3.57644800	-0.33601000
H	3.35278800	-0.40542100	-2.74433500
H	4.77981400	0.46288000	-3.38495300
H	4.74221600	-0.06283000	-1.67583400
H	5.09858700	5.54906800	-0.60241800
H	4.19911000	6.75948900	-1.55897100
H	3.83303500	6.53853800	0.17519900
H	3.34081600	4.43476900	-2.04036800
H	2.89406000	2.09196700	-2.91582800
H	2.53778900	4.43355500	0.93278700
H	3.87037700	3.47239800	0.28726700
H	3.22606900	1.09744100	-0.01919100
H	1.83109500	0.73310500	-1.06236500
H	4.28998700	2.40888000	-1.86551200
H	2.08239300	5.42528200	-1.29129300
H	1.19194400	3.12577200	-1.10032300
H	-1.41377700	0.38788700	2.54999300
H	0.38195800	0.62731000	2.95838300
H	3.28325000	-2.98715900	-0.97979800
H	-2.57157200	-0.12946200	1.16722200
H	-3.13415000	-2.11509200	-1.11636500
H	-7.36209000	0.90542200	2.27144900
H	-8.47358400	0.11151300	-2.56743600
H	-10.12553900	1.19017300	-1.04145500
H	-9.56585600	1.58359900	1.35311700
H	-5.82646000	-0.57400800	2.73427600
H	-4.22565700	-1.18667700	2.24643400
H	-4.50748500	0.59355600	2.37802700

Second transition state (TS_2)

C	5.27706800	-1.30291900	-0.33868600
C	4.28829800	-0.60752200	-1.08776200
C	2.94703400	-0.85188600	-0.85908000
O	2.05230000	-0.14395900	-1.63068600
C	0.67727000	-0.23337100	-1.50449600
C	0.16050500	-1.19379500	-0.53245900
C	1.05802100	-1.91945100	0.26333600
C	2.46403700	-1.77165900	0.10715600
C	3.46728900	-2.45226200	0.86049100
C	4.81048500	-2.23409800	0.65514200
O	0.01225100	0.51734800	-2.22716200
C	-1.26646300	-1.33525100	-0.35999700
C	0.51249800	-2.80012900	1.36630700

N	6.60499500	-1.08922800	-0.55523400
C	7.00078200	-0.05925000	-1.54048500
C	7.61743600	-1.75135700	0.28169400
C	7.82773700	-1.04779500	1.62606300
C	8.50275300	0.09334900	-1.75422800
O	-0.59874500	0.60532400	2.05227500
C	0.28974700	1.39992900	1.42534200
C	-0.22659800	2.80282700	1.19443300
C	0.86693600	3.73070800	0.63412200
C	-1.48290200	2.71866300	0.28828600
O	1.40222900	0.98622100	1.06059300
C	-2.12894100	4.08010500	0.00524600
C	0.85363600	5.14163300	1.24143900
C	1.91796800	6.04969600	0.61657700
C	-3.42579200	3.95078100	-0.80100800
C	-2.25974900	-0.68008300	-1.04183000
C	-3.65693000	-0.82874800	-0.77594900
S	-4.74778700	0.52394500	-1.16343600
N	-4.31713600	-1.87751200	-0.26020500
C	-5.70598800	-1.67465200	-0.06924800
C	-6.13548500	-0.41985500	-0.52412700
C	-6.61494900	-2.57914200	0.49682100
C	-7.47250800	-0.03536200	-0.44370500
C	-7.95472400	-2.19496700	0.58196900
C	-8.38308700	-0.93772300	0.11423900
C	-3.71707300	-3.19684600	-0.00135000
O	-0.13471700	-1.91517300	2.32111400
H	3.18355000	-3.17099800	1.62886500
H	5.51935700	-2.77991000	1.27348900
H	7.32393500	-2.80208800	0.42870600
H	8.55913500	-1.78504500	-0.28000700
H	6.88922000	-1.00953500	2.20602700
H	8.58364700	-1.58296500	2.22494900
H	8.17688800	-0.01224300	1.47236400
H	6.52970700	-0.31041100	-2.50721800
H	6.57720700	0.91180700	-1.22132800
H	8.66481000	0.90128200	-2.48617700
H	9.03185500	0.37133200	-0.82795500
H	8.95746300	-0.82339100	-2.16387200
H	-4.18425900	3.37907100	-0.23725100
H	-3.85138200	4.94078100	-1.03874100
H	-3.24505100	3.41987300	-1.75381700
H	1.73905200	6.17549900	-0.46659900
H	1.91843000	7.05140500	1.07925100
H	2.92621600	5.61534400	0.74241900
H	-0.14409100	5.59986700	1.11977300
H	-2.33608200	4.59204900	0.96432900
H	1.85667100	3.27785300	0.81690800
H	0.76123300	3.79672700	-0.46717500
H	-1.19156200	2.24222400	-0.66960800
H	-2.22200800	2.05125000	0.76905000
H	-1.41733500	4.72102000	-0.54662000
H	1.02496300	5.05885400	2.33153500
H	-0.55647600	3.17345800	2.18498700
H	-0.21539600	-3.52543200	0.96807500
H	1.30710600	-3.36169500	1.86887900
H	4.53746500	0.12793200	-1.84729000
H	-1.56272100	-1.99777200	0.45396000
H	-1.99503500	0.07892200	-1.77953900
H	-6.29359000	-3.54955500	0.87450300
H	-7.80044600	0.94157800	-0.80260600
H	-9.43516500	-0.66008700	0.19086100
H	-8.67765000	-2.88255100	1.02288200
H	-4.47403800	-3.96663300	-0.20047500
H	-2.87569700	-3.35045700	-0.69089000
H	-3.37722300	-3.27554800	1.04314500
H	-0.45232500	-2.37870500	3.12655000
H	-0.32719900	-0.37453600	2.16411100

Z-form S_1 state

C	1.39359900	3.98883200	-0.11323400
C	0.40345200	3.22406200	0.53380400
C	0.17985100	1.91971800	0.14934600
O	-0.78879800	1.25602600	0.84078400
C	-1.14896400	-0.02959100	0.58274600
C	-0.47767700	-0.70363900	-0.52480900
C	0.55027500	-0.07425100	-1.18367700
C	0.89630000	1.27275000	-0.86724500
C	1.91214900	2.03807500	-1.48122400

C	2.15937600	3.33341200	-1.12461700
O	-1.99362500	-0.51201800	1.30320200
C	-0.86974200	-2.07969400	-0.84615300
C	1.28112900	-0.82754700	-2.27815400
N	1.60609900	5.29237700	0.21321500
C	0.89239500	5.83669700	1.37705200
C	2.80641200	5.97906400	-0.26253800
C	4.05398300	5.59005600	0.52260900
C	1.09761500	7.32272300	1.61199400
O	1.97225300	-1.95719100	-1.72914900
C	3.10639800	-1.69570500	-1.04628400
C	3.59360400	-2.91448200	-0.30661600
C	5.09640400	-2.83917800	-0.00892600
C	2.72049100	-3.01222900	0.96278700
O	3.59715900	-0.59711300	-1.01011700
C	3.01810400	-4.23196900	1.82715500
C	5.80415900	-4.18508400	-0.15508200
C	7.27623900	-4.09698700	0.23000200
C	2.06321900	-4.31884900	3.01319900
C	-2.09931400	-2.60437900	-0.81845100
C	-3.31673100	-1.83009700	-0.61220300
S	-3.70796600	-0.48073600	-1.60378400
N	-4.24433100	-2.09994200	0.27980400
C	-5.32850300	-1.21954200	0.26630200
C	-5.19509200	-0.24317700	-0.71895400
C	-6.43546100	-1.25117800	1.10982900
C	-6.16905300	0.73362500	-0.89882100
C	-7.39904700	-0.27747300	0.93079400
C	-7.26856000	0.70295800	-0.06145600
C	-4.11456100	-3.13272200	1.30902200
H	2.53706300	1.59151200	-2.24002100
H	2.96254000	3.85132800	-1.62380100
H	2.93102500	5.77705300	-1.32428700
H	2.63290900	7.04840600	-0.18937300
H	4.24452100	4.51856700	0.44450000
H	4.92482400	6.12434100	0.14170700
H	3.93287700	5.83852100	1.57847800
H	-0.17349700	5.66392700	1.22109800
H	1.18267500	5.27870500	2.27544200
H	0.46624800	7.62064900	2.44865200
H	2.12766500	7.56669700	1.87079100
H	0.80240300	7.90980900	0.74201600
H	1.02866500	-4.39904300	2.67290100
H	2.28196300	-5.18643000	3.63637700
H	2.14100800	-3.42659400	3.63784200
H	7.38490400	-3.79967400	1.27525600
H	7.78130900	-5.05443900	0.09825800
H	7.79337300	-3.35535700	-0.38260600
H	5.30692000	-4.94020800	0.45782900
H	2.93806700	-5.13628400	1.21682000
H	5.56854900	-2.12197400	-0.68398900
H	5.24671100	-2.44882800	1.00282900
H	2.86887700	-2.10321700	1.55621700
H	1.66638200	-3.03383600	0.67002400
H	4.04776100	-4.18495600	2.19043000
H	5.71120200	-4.51940100	-1.19239800
H	3.37821600	-3.78336400	-0.93549000
H	0.56741300	-1.24540300	-2.98462100
H	1.98081100	-0.19781500	-2.81591700
H	-0.19657900	3.61201700	1.34094500
H	-0.06518400	-2.75304300	-1.11628800
H	-2.23926700	-3.66158000	-1.01132400
H	-6.53825400	-2.00757900	1.87564900
H	-6.06480000	1.48971300	-1.66464700
H	-8.04170000	1.45073400	-0.17516500
H	-8.27113300	-0.27341300	1.57031500
H	-4.94303700	-3.83160600	1.21917400
H	-3.17046200	-3.64898300	1.17589700
H	-4.12850300	-2.64705900	2.28294500

E-form TS_1 state

C	3.01123000	2.56502200	-0.36898200
C	1.83285700	2.20255900	0.33939600
C	0.96413800	1.27439000	-0.20511000
O	-0.16377900	0.96629700	0.53986400
C	-1.12997200	0.05479600	0.16862300
C	-0.92837400	-0.63880500	-1.10901700
C	0.20027600	-0.33523500	-1.88631500

C	1.17217600	0.63226800	-1.44731600
C	2.35286100	1.00028300	-2.14792500
C	3.23898000	1.92206500	-1.63682700
O	-2.07065800	-0.12384500	0.94927800
C	-1.79311900	-1.77605300	-1.42833800
C	0.40011700	-1.04604400	-3.12268400
N	3.88727900	3.47700400	0.14031400
C	3.62045700	4.03354100	1.48413400
C	5.15230300	3.76840600	-0.55140900
C	6.23082900	2.71505900	-0.27821400
C	4.62595400	5.07179300	1.96953100
O	0.90439500	-3.13079800	-1.92878900
C	2.04559300	-2.75466800	-1.40394400
C	2.27081100	-3.24514800	0.03483100
C	3.73579100	-3.09515200	0.47560700
C	1.27609800	-2.41138700	0.88074600
O	2.84646100	-1.95531000	-1.94696000
C	1.33337700	-2.62232400	2.39745100
C	4.19932600	-4.18210400	1.45687500
C	5.63934300	-3.95719500	1.93049700
C	0.27298900	-1.77491500	3.11181000
C	-3.11704700	-1.90327500	-1.17347800
C	-3.97811000	-0.82370100	-0.71866200
S	-4.01864700	0.74428400	-1.53689600
N	-4.81856700	-0.89711900	0.30136400
C	-5.54782500	0.28464000	0.56006200
C	-5.23569300	1.31206500	-0.34127200
C	-6.47895800	0.47942200	1.58851000
C	-5.84418100	2.56318800	-0.24974300
C	-7.08727500	1.73155900	1.68251700
C	-6.77457200	2.76315600	0.77385900
C	-4.89831800	-2.04560800	1.21837000
H	2.58883200	0.53328300	-3.10321400
H	4.13425500	2.14444500	-2.21267600
H	4.95486200	3.86003800	-1.63041600
H	5.49911700	4.75851900	-0.23079500
H	5.89765500	1.71226000	-0.59789900
H	7.15638200	2.96309500	-0.82428800
H	6.46525600	2.66987000	0.79922900
H	2.61617300	4.49282200	1.47153400
H	3.58078300	3.19761000	2.20821900
H	4.32976400	5.38911000	2.98258600
H	5.64919100	4.66665800	2.03263900
H	4.63579100	5.96872300	1.32893400
H	-0.74163600	-2.03494100	2.75979900
H	0.30591900	-1.92165600	4.20485000
H	0.42960000	-0.69991300	2.90586000
H	5.72904500	-2.99680000	2.46975100
H	5.97311000	-4.76038100	2.60952800
H	6.33381600	-3.92638600	1.07151400
H	3.52329600	-4.22344000	2.32935300
H	1.17996400	-3.69376000	2.62556900
H	4.38713100	-3.12110200	-0.41566100
H	3.88095200	-2.09219200	0.92524700
H	1.46619700	-1.33591900	0.66837000
H	0.25073400	-2.63463500	0.52910300
H	2.33665300	-2.35557800	2.77626500
H	4.11879600	-5.16631600	0.95753400
H	1.96769900	-4.30649300	0.08378500
H	-0.43036600	-1.56719200	-3.60562300
H	1.33175600	-0.94155200	-3.68258900
H	1.57537600	2.62141000	1.30801500
H	-1.26747400	-2.64550800	-1.84175100
H	-3.60029900	-2.86886200	-1.35545500
H	-6.72317200	-0.31720400	2.29155300
H	-5.60248200	3.36256500	-0.95184700
H	-7.26393600	3.73333400	0.86916300
H	-7.81646100	1.91229900	2.47320200
H	-5.92581300	-2.43770400	1.22672600
H	-4.20253000	-2.82628900	0.88854600
H	-4.61029300	-1.70631900	2.22529800

E-form TS_2 state

C	-2.91240200	1.48626900	1.35854500
C	-1.72301300	1.56454000	0.58551500
C	-0.73984200	0.60227900	0.73694600
O	0.38144500	0.73402300	-0.06235500
C	1.42068900	-0.17963200	-0.10319600
C	1.33086600	-1.31833800	0.80705400

C	0.21870200	-1.46547900	1.62684600
C	-0.84176400	-0.50197100	1.61547600
C	-2.03087000	-0.55782000	2.39613000
C	-3.02213700	0.39266900	2.28384500
O	2.32422100	0.04551000	-0.91821100
C	2.37256900	-2.35262400	0.75637900
C	0.12699400	-2.69088800	2.51512500
N	-3.91149900	2.40447300	1.20739700
C	-3.78687700	3.40453300	0.12690100
C	-5.13703200	2.31249700	2.01400800
C	-6.15163600	1.31026700	1.45531900
C	-4.96774200	4.35924200	-0.01228000
O	-1.16721500	-3.17772900	-0.56700300
C	-2.40680300	-2.66017900	-0.44310500
C	-2.86155100	-1.86621000	-1.65687500
C	-4.07088600	-0.97394500	-1.31562700
C	-1.65645300	-1.09639100	-2.24343400
O	-3.08345900	-2.84096700	0.58018200
C	-1.96455700	-0.11954100	-3.38216700
C	-5.08860600	-0.84904300	-2.45951700
C	-6.22126200	0.12709900	-2.12790600
C	-0.68657900	0.57758200	-3.86654800
C	3.69005500	-2.20205200	0.48333600
C	4.37392600	-0.92498800	0.36548100
S	4.28089600	0.30145200	1.63913300
N	5.14717700	-0.55962000	-0.64524700
C	5.70127000	0.73546200	-0.53766800
C	5.32778400	1.37833800	0.65044800
C	6.52229400	1.36972800	-1.47874000
C	5.76462000	2.67005400	0.94052800
C	6.95886000	2.66357600	-1.19075000
C	6.58553000	3.30795800	0.00603000
C	5.30603800	-1.34588100	-1.87828000
H	-2.19262600	-1.38493400	3.08668400
H	-3.91521800	0.27554500	2.89332900
H	-4.85743100	2.05287900	3.04707600
H	-5.58652300	3.31209900	2.07276800
H	-5.69860700	0.31012600	1.33422000
H	-7.01771600	1.22066900	2.13286700
H	-6.51730900	1.63642600	0.46729300
H	-2.86934700	3.99620000	0.29816800
H	-3.64264400	2.86389000	-0.82885600
H	-4.78461400	5.01017400	-0.88263400
H	-5.91611700	3.82488600	-0.18727800
H	-5.08233200	5.00935500	0.87023500
H	0.04201700	-0.16015500	-4.24835300
H	-0.89821400	1.29863100	-4.67434700
H	-0.20444100	1.12351800	-3.03493000
H	-5.82100700	1.14040600	-1.93641100
H	-6.94839300	0.20000600	-2.95476600
H	-6.76537000	-0.19581400	-1.22225700
H	-4.58431900	-0.52785700	-3.38735600
H	-2.44226300	-0.65767900	-4.22251800
H	-4.58488500	-1.38750100	-0.43013400
H	-3.70679200	0.03289000	-1.02086300
H	-1.18072700	-0.52780300	-1.42405600
H	-0.90499400	-1.82818200	-2.58565200
H	-2.68861700	0.64327700	-3.03995100
H	-5.50781300	-1.85190100	-2.66791700
H	-3.18428700	-2.62303300	-2.40296500
H	1.07676500	-2.83550100	3.05404100
H	-0.67820800	-2.59835700	3.25355600
H	-1.54215900	2.35536300	-0.13756900
H	2.02069800	-3.37677700	0.91630500
H	4.31581600	-3.09537600	0.38417200
H	6.81126300	0.87261300	-2.40501900
H	5.47446300	3.16975700	1.86596500
H	6.93953300	4.31981300	0.20777100
H	7.59889000	3.18219200	-1.90561300
H	6.37152900	-1.56624800	-2.03915500
H	4.73997200	-2.28077400	-1.78835900
H	4.90575100	-0.75952000	-2.71965400
O	-0.12026500	-3.83721400	1.65350500
H	-0.17847800	-4.68719200	2.14208000
H	-0.79825600	-3.57496000	0.30305900

5d(PP) ground state

C	-5.11674400	0.09054100	-0.29802600
C	-4.16924100	-0.90830600	0.00733800

C	-2.82431500	-0.62031900	-0.03322900
O	-1.99133000	-1.64783900	0.27911000
C	-0.63123500	-1.53514700	0.30509000
C	-0.05850100	-0.24285200	-0.05591500
C	-0.89402400	0.81568300	-0.36840600
C	-2.30446200	0.64684500	-0.35392900
C	-3.26964300	1.64660200	-0.62806300
C	-4.60968700	1.39358400	-0.59613200
O	-0.01885300	-2.52694400	0.62074100
C	1.37517200	-0.07990500	-0.07103900
C	-0.29105700	2.15991700	-0.71169000
N	-6.44533100	-0.17720100	-0.30690100
C	-6.89145500	-1.49658600	0.16665000
C	-7.41587700	0.91357800	-0.39913800
C	-7.60353200	1.64203700	0.92665900
C	-8.38126000	-1.75447400	0.03000300
C	2.32458200	-0.99148200	0.23347200
C	3.72856600	-0.69191700	0.14715200
S	4.83647300	-1.92645400	-0.33470500
N	4.33428500	0.46375900	0.39855100
C	5.71479700	0.44934800	0.16747100
C	6.17001200	-0.80609000	-0.22982100
C	6.59342600	1.52144400	0.30026600
C	7.51642300	-1.03750200	-0.48715200
C	7.93072800	1.29054000	0.03733100
C	8.38935700	0.02605700	-0.34966200
C	3.69036900	1.64982000	0.97409100
O	0.41956900	2.62599800	0.42895500
H	-2.95714600	2.65402300	-0.85961000
H	-5.28439700	2.20983800	-0.79846600
H	-7.10192600	1.59824500	-1.18424500
H	-8.35919600	0.49610800	-0.73719700
H	-6.66265700	2.07424100	1.27047500
H	-8.33327800	2.44478100	0.81770200
H	-7.96180800	0.95291700	1.69320400
H	-6.36172300	-2.25331800	-0.41406000
H	-6.59135200	-1.62197400	1.21338200
H	-8.57504100	-2.77477700	0.35976100
H	-8.97963100	-1.08655000	0.64911700
H	-8.71048100	-1.66829700	-1.00560600
H	0.37733400	2.04059300	-1.57037300
H	-1.06026600	2.87011500	-0.99888600
H	-4.45181000	-1.91153300	0.28234600
H	1.70892900	0.89862800	-0.38383800
H	2.07105100	-2.01286800	0.47341400
H	6.24625300	2.50438500	0.58554400
H	7.86343700	-2.01554300	-0.79063800
H	9.44237700	-0.12228200	-0.54689700
H	8.63421100	2.10684100	0.12883100
H	4.37659900	2.08469100	1.69530200
H	3.46348800	2.37164400	0.19103700
H	2.77750100	1.35220300	1.47926500
H	0.71381600	3.52703100	0.25380600

Valproic acid ground state

O	2.73361000	-0.63010800	-0.83608000
C	1.76427900	-0.93643100	0.04202800
C	0.45763200	-0.26060800	-0.29292900
C	-0.73806900	-0.98882100	0.33484000
C	0.58707500	1.20823200	0.16306200
O	1.97142800	-1.64072200	0.99884700
C	-0.63922500	2.05978100	-0.14646600
C	-1.95542300	-1.04925800	-0.58635900
C	-3.15113100	-1.70452000	0.09487900
C	-0.42358600	3.51588000	0.25257100
H	0.42036800	3.94628300	-0.29014400
H	-1.30564000	4.12050300	0.03932700
H	-0.21014000	3.59635300	1.32048500
H	-3.45457000	-1.13395300	0.97536400
H	-4.00805800	-1.76783600	-0.57663400
H	-2.90391500	-2.71647400	0.42296200
H	-2.22607000	-0.04505700	-0.92000100
H	-0.86358500	1.99585100	-1.21532600
H	-0.44960600	-2.00891000	0.59697100
H	-1.00952500	-0.49875300	1.27558700
H	0.77108500	1.22105400	1.24298700
H	1.46184600	1.65441000	-0.31756700
H	-1.50886800	1.66300000	0.38322900
H	-1.68707700	-1.61125200	-1.48563400

H	0.36579800	-0.26839700	-1.38333200
H	3.55834100	-1.04570300	-0.54016700

Coordinates for 5d in Methanol/ glycerol

Ground state

C	-4.30709100	-2.03301600	-0.46153800
C	-3.23141500	-2.81851700	0.00219200
C	-1.93675000	-2.38880700	-0.18173600
O	-0.95882000	-3.21087600	0.28474000
C	0.37070400	-2.92036600	0.20357100
C	0.75374000	-1.62407000	-0.36623600
C	-0.22990200	-0.80767600	-0.88953700
C	-1.59891500	-1.18510800	-0.81514200
C	-2.68569300	-0.40695800	-1.27815800
C	-3.98107000	-0.80043000	-1.10924500
O	1.12771200	-3.76144000	0.62130400
C	2.14296500	-1.22715300	-0.32719600
C	0.06180400	0.53058800	-1.52353800
N	-5.59300900	-2.43688600	-0.30827100
C	-5.84986500	-3.63599500	0.50400900
C	-6.68944400	-1.50252000	-0.56531400
C	-6.86775200	-0.49167300	0.56158100
C	-7.30221300	-4.07519900	0.55310100
O	-0.34304200	1.52480900	-0.57483600
C	-1.00121400	2.60816900	-1.03498100
C	-1.52489800	3.44763200	0.10206500
C	-1.75783000	4.90315900	-0.32320400
C	-2.80536900	2.73968500	0.59632400
O	-1.18954200	2.80510200	-2.20809900
C	-3.46361300	3.41560200	1.79344400
C	-1.37099100	5.91040100	0.75838500
C	-1.70082200	7.34039000	0.34699100
C	-4.68762500	2.63824000	2.26574700
C	3.19094600	-1.90799700	0.18265800
C	4.52117000	-1.35619400	0.23323700
S	5.52937100	-1.69724200	1.59192300
N	5.12047300	-0.58040200	-0.66190200
C	6.40509200	-0.16550300	-0.29276300
C	6.80174500	-0.70478900	0.92905700
C	7.24554300	0.68133100	-1.01096200
C	8.05524600	-0.43137400	1.46537400
C	8.48860700	0.95578200	-0.47306600
C	8.89133600	0.40515100	0.74942700
C	4.58655800	-0.24613600	-1.98630200
H	-2.50495400	0.53466400	-1.78076800
H	-4.76033300	-0.14892300	-1.47100900
H	-6.51333700	-1.00470300	-1.51660200
H	-7.59809500	-2.07999800	-0.70344900
H	-5.95857800	0.09426000	0.70435900
H	-7.68647700	0.19154700	0.33392700
H	-7.09474000	-1.00137700	1.49952400
H	-5.26308700	-4.45341100	0.08209200
H	-5.48256200	-3.46542200	1.52273700
H	-7.35252300	-4.99990400	1.12729000
H	-7.94367900	-3.34436600	1.04492000
H	-7.69414100	-4.27734700	-0.44388500
H	-4.41632800	1.61752700	2.54427400
H	-5.15070500	3.11298500	3.13125900
H	-5.43648100	2.58037700	1.47295200
H	-2.77421600	7.45707100	0.18281800
H	-1.39776200	8.05578700	1.11218600
H	-1.19124300	7.60396000	-0.58230500
H	-1.87827300	5.66956000	1.69528300
H	-2.73638000	3.49884100	2.60635900
H	-1.17975600	5.11994500	-1.22413800
H	-2.80924700	5.03746300	-0.59700000
H	-3.52120800	2.69856500	-0.23313200
H	-2.56163400	1.70566200	0.85908300
H	-3.76042400	4.43329800	1.52808400
H	-0.29909700	5.81840200	0.95573900
H	-0.78235900	3.40539200	0.90411300
H	1.10646100	0.68633400	-1.76835200
H	-0.50645100	0.64054800	-2.44521800
H	-3.37542900	-3.75628900	0.51368500
H	2.34331200	-0.23368200	-0.70153400
H	3.05932700	-2.86229700	0.66950300
H	6.93735000	1.12124100	-1.94891300
H	8.35962800	-0.85553500	2.41226000
H	9.87143900	0.63891600	1.14253800

H	9.16043800	1.61359000	-1.00740200
H	5.40287800	-0.30716500	-2.70059900
H	3.82461200	-0.97029300	-2.25458100
H	4.17347500	0.76103400	-1.98001100

Excited (S_1) state

C	5.58429800	-0.73724900	0.29836000
C	4.66784100	-1.46049500	-0.49058500
C	3.31870800	-1.37662300	-0.23330200
O	2.51536700	-2.08731600	-1.06769300
C	1.15515200	-2.09244400	-0.97888000
C	0.54471200	-1.32219600	0.10337900
C	1.35336700	-0.58399000	0.95359800
C	2.76419000	-0.59141100	0.79295400
C	3.69555700	0.16081400	1.54972600
C	5.03811800	0.10049100	1.32036900
O	0.57186100	-2.76808700	-1.79170300
C	-0.89029400	-1.34393800	0.25953900
C	0.73433900	0.22101800	2.08133500
N	6.91975700	-0.83250900	0.08924800
C	7.37923700	-1.58205500	-1.09194100
C	7.83481900	0.10851800	0.73948600
C	7.85796400	1.46892800	0.05201000
C	8.88329600	-1.77028400	-1.19418300
O	-0.29137500	1.11253200	1.63393700
C	0.12015600	2.10980400	0.82182500
C	-1.03729600	2.96013300	0.34367800
C	-0.55831300	4.07143100	-0.59937500
C	-2.10837800	2.04400100	-0.27293000
O	1.28145700	2.24904000	0.53953400
C	-3.33670600	2.79444100	-0.77500100
C	-1.27620600	5.40326600	-0.38463200
C	-0.81912500	6.46046800	-1.38323200
C	-4.43455500	1.83553000	-1.21635700
C	-1.80645100	-1.95228900	-0.52901200
C	-3.22294400	-1.81842900	-0.31804900
S	-4.28489200	-1.87450200	-1.67984400
N	-3.87359100	-1.64070600	0.82675100
C	-5.25732400	-1.48570700	0.68131000
C	-5.66458500	-1.60857600	-0.64636100
C	-6.17981500	-1.22824500	1.69170300
C	-7.00347200	-1.49704000	-1.00431500
C	-7.50928700	-1.10879900	1.33183100
C	-7.91903500	-1.24328900	0.00033300
C	-3.25920600	-1.69814500	2.15710800
H	3.35348500	0.83048800	2.32453600
H	5.68313300	0.71906100	1.92354400
H	7.55650700	0.20253400	1.78700900
H	8.82784700	-0.32951700	0.74246300
H	6.86715300	1.92628800	0.05796400
H	8.55123300	2.13884700	0.56155800
H	8.17977500	1.36613400	-0.98593400
H	6.91636300	-2.56966400	-1.05640500
H	7.00909000	-1.08438200	-1.99581100
H	9.08526600	-2.39703100	-2.06267300
H	9.41355000	-0.82861200	-1.33503400
H	9.28423400	-2.27596100	-0.31536800
H	-4.79608300	1.25179800	-0.36682500
H	-5.28764600	2.37204300	-1.63378700
H	-4.06588400	1.14023200	-1.97479700
H	-1.04590400	6.14940700	-2.40558200
H	-1.31163500	7.41735900	-1.20551000
H	0.25968500	6.61758500	-1.31466100
H	-2.35726300	5.26944600	-0.46113300
H	-3.71591700	3.44253000	0.02080100
H	0.51239200	4.22922200	-0.45818600
H	-0.68548500	3.74500200	-1.63713400
H	-1.66348900	1.47815600	-1.10065800
H	-2.41922900	1.31256700	0.47749700
H	-3.06086700	3.44537100	-1.60860100
H	-1.07828400	5.74595400	0.63554500
H	-1.46360000	3.40733800	1.25003200
H	0.24112200	-0.45030600	2.78341800
H	1.48054000	0.79323800	2.61949800
H	4.97679600	-2.07774900	-1.31924600
H	-1.26144600	-0.76335100	1.09257900
H	-1.51464300	-2.47558900	-1.42658200
H	-5.87189700	-1.10806300	2.72044900
H	-7.31292100	-1.59602500	-2.03584900

H	-8.96748300	-1.14659600	-0.24741900
H	-8.24495700	-0.90096900	2.09700500
H	-3.93943000	-2.22879200	2.81869600
H	-2.32528800	-2.24835000	2.09069900
H	-3.08474000	-0.69028700	2.53449500

First transition state (TS_1)

C	4.15334000	-1.76364200	0.64281700
C	3.10360700	-2.32848700	-0.13557200
C	1.79063800	-2.03051200	0.16634500
O	0.82265700	-2.61024400	-0.63164000
C	-0.53837200	-2.38515900	-0.51427800
C	-0.96456600	-1.38207800	0.47101600
C	0.00480000	-0.82891900	1.34207600
C	1.39574300	-1.16661600	1.21881800
C	2.45185500	-0.66080600	2.03272300
C	3.77122200	-0.94217400	1.76206500
O	-1.26983200	-3.03331600	-1.26352300
C	-2.33977200	-0.94673300	0.48082000
C	-0.36992700	0.16078400	2.31961900
N	5.45737700	-1.98694800	0.32832900
C	5.76638300	-2.80809800	-0.86442500
C	6.53204900	-1.30611900	1.06928400
C	6.73997500	0.13819000	0.60189700
C	7.25030200	-3.00808700	-1.14965500
O	-0.11233900	1.86849600	0.60625500
C	1.08889300	2.31126000	0.88692900
C	1.89552200	2.77877100	-0.32825000
C	2.87605300	3.91216700	0.02653400
C	2.59890100	1.49783500	-0.84444400
O	1.62003600	2.27153800	2.02631800
C	3.48277100	1.67907100	-2.08289100
C	3.04438400	4.94581000	-1.09727200
C	4.08854900	6.01338100	-0.75361200
C	4.12246200	0.34946100	-2.50273200
C	-3.34718100	-1.38398300	-0.33782900
C	-4.68685000	-0.86615900	-0.32995900
S	-5.61502000	-0.87164400	-1.84470500
N	-5.38849300	-0.36570900	0.69510800
C	-6.67905800	0.10687200	0.35827500

C	-6.99224600	-0.09924900	-0.99321900
C	-7.59871600	0.71709000	1.22325300
C	-8.22577200	0.28151400	-1.51958900
C	-8.83244000	1.10203900	0.69747400
C	-9.14682100	0.88458900	-0.65908200
C	-4.95056000	-0.38336700	2.10192400
H	2.23423200	0.00059000	2.86946200
H	4.52957900	-0.50136200	2.40487400
H	6.30319100	-1.34457100	2.14493600
H	7.45594500	-1.88325600	0.94330400
H	5.80978800	0.72650600	0.70064300
H	7.52383200	0.62705900	1.20428900
H	7.05052800	0.16336700	-0.45675000
H	5.29020700	-3.79587800	-0.73241400
H	5.28814800	-2.33668500	-1.74368900
H	7.34377500	-3.59940000	-2.07496100
H	7.77742900	-2.05309400	-1.30925600
H	7.75648000	-3.56661000	-0.34562500
H	3.34856200	-0.40222000	-2.74364900
H	4.77209000	0.47002700	-3.38663000
H	4.73879400	-0.05572100	-1.67738400
H	5.08289100	5.55551100	-0.60279000
H	4.17901600	6.76488600	-1.55649600
H	3.81686700	6.54164900	0.17819200
H	3.32454300	4.43881600	-2.03826300
H	2.88267400	2.09385600	-2.91453300
H	2.52722400	4.43305300	0.93651500
H	3.86077900	3.47571700	0.28773500
H	3.22172500	1.10007100	-0.01839400
H	1.82610900	0.73228200	-1.05968000
H	4.27925800	2.41472700	-1.86635300
H	2.06559900	5.42608400	-1.28582200
H	1.18112600	3.12372000	-1.09606000
H	-1.41218600	0.37887100	2.55640600
H	0.38354000	0.62176000	2.96301200
H	3.28633000	-2.98505700	-0.98163100
H	-2.56897300	-0.13418300	1.17003800
H	-3.12977900	-2.11749200	-1.11607000
H	-7.36042500	0.90098200	2.27060800

H	-8.46579200	0.11574700	-2.57102300
H	-10.11890000	1.19343200	-1.04570300
H	-9.56223400	1.58260700	1.35023200
H	-5.82716800	-0.58085900	2.73316000
H	-4.22755000	-1.19725600	2.24599400
H	-4.50432800	0.58355800	2.38137300

Second transition state (TS_2)

C	5.27706800	-1.30291900	-0.33868600
C	4.28829800	-0.60752200	-1.08776200
C	2.94703400	-0.85188600	-0.85908000
O	2.05230000	-0.14395900	-1.63068600
C	0.67727000	-0.23337100	-1.50449600
C	0.16050500	-1.19379500	-0.53245900
C	1.05802100	-1.91945100	0.26333600
C	2.46403700	-1.77165900	0.10715600
C	3.46728900	-2.45226200	0.86049100
C	4.81048500	-2.23409800	0.65514200
O	0.01225100	0.51734800	-2.22716200
C	-1.26646300	-1.33525100	-0.35999700
C	0.51249800	-2.80012900	1.36630700
N	6.60499500	-1.08922800	-0.55523400
C	7.00078200	-0.05925000	-1.54048500
C	7.61743600	-1.75135700	0.28169400
C	7.82773700	-1.04779500	1.62606300
C	8.50275300	0.09334900	-1.75422800
O	-0.59874500	0.60532400	2.05227500
C	0.28974700	1.39992900	1.42534200
C	-0.22659800	2.80282700	1.19443300
C	0.86693600	3.73070800	0.63412200
C	-1.48290200	2.71866300	0.28828600
O	1.40222900	0.98622100	1.06059300
C	-2.12894100	4.08010500	0.00524600
C	0.85363600	5.14163300	1.24143900
C	1.91796800	6.04969600	0.61657700
C	-3.42579200	3.95078100	-0.80100800
C	-2.25974900	-0.68008300	-1.04183000
C	-3.65693000	-0.82874800	-0.77594900
S	-4.74778700	0.52394500	-1.16343600
N	-4.31713600	-1.87751200	-0.26020500
C	-5.70598800	-1.67465200	-0.06924800
C	-6.13548500	-0.41985500	-0.52412700
C	-6.61494900	-2.57914200	0.49682100
C	-7.47250800	-0.03536200	-0.44370500
C	-7.95472400	-2.19496700	0.58196900
C	-8.38308700	-0.93772300	0.11423900
C	-3.71707300	-3.19684600	-0.00135000
O	-0.13471700	-1.91517300	2.32111400
H	3.18355000	-3.17099800	1.62886500
H	5.51935700	-2.77991000	1.27348900
H	7.32393500	-2.80208800	0.42870600
H	8.55913500	-1.78504500	-0.28000700
H	6.88922000	-1.00953500	2.20602700
H	8.58364700	-1.58296500	2.22494900
H	8.17688800	-0.01224300	1.47236400
H	6.52970700	-0.31041100	-2.50721800
H	6.57720700	0.91180700	-1.22132800
H	8.66481000	0.90128200	-2.48617700
H	9.03185500	0.37133200	-0.82795500
H	8.95746300	-0.82339100	-2.16387200
H	-4.18425900	3.37907100	-0.23725100
H	-3.85138200	4.94078100	-1.03874100
H	-3.24505100	3.41987300	-1.75381700
H	1.73905200	6.17549900	-0.46659900
H	1.91843000	7.05140500	1.07925100
H	2.92621600	5.61534400	0.74241900
H	-0.14409100	5.59986700	1.11977300
H	-2.33608200	4.59204900	0.96432900
H	1.85667100	3.27785300	0.81690800
H	0.76123300	3.79672700	-0.46717500
H	-1.19156200	2.24222400	-0.66960800
H	-2.22200800	2.05125000	0.76905000
H	-1.41733500	4.72102000	-0.54662000
H	1.02496300	5.05885400	2.33153500
H	-0.55647600	3.17345800	2.18498700
H	-0.21539600	-3.52543200	0.96807500
H	1.30710600	-3.36169500	1.86887900
H	4.53746500	0.12793200	-1.84729000
H	-1.56272100	-1.99777200	0.45396000

H	-1.99503500	0.07892200	-1.77953900
H	-6.29359000	-3.54955500	0.87450300
H	-7.80044600	0.94157800	-0.80260600
H	-9.43516500	-0.66008700	0.19086100
H	-8.67765000	-2.88255100	1.02288200
H	-4.47403800	-3.96663300	-0.20047500
H	-2.87569700	-3.35045700	-0.69089000
H	-3.37722300	-3.27554800	1.04314500
H	-0.45232500	-2.37870500	3.12655000
H	-0.32719900	-0.37453600	2.16411100

Z-form S_1 state

C	1.41476700	3.98215800	-0.11400500
C	0.41912200	3.22314900	0.53144700
C	0.18856700	1.92018400	0.14665800
O	-0.78477800	1.26203300	0.83666000
C	-1.15156500	-0.02170000	0.57853300
C	-0.48268600	-0.69945300	-0.52829300
C	0.54939100	-0.07554700	-1.18624000
C	0.90253200	1.26931500	-0.86934700
C	1.92375600	2.02881500	-1.48177700
C	2.17800100	3.32253700	-1.12459300
O	-1.99950200	-0.49955600	1.29804700
C	-0.88035400	-2.07391700	-0.84872400
C	1.27738700	-0.83295000	-2.27984800
N	1.63472200	5.28417600	0.21322300
C	0.92258200	5.83252000	1.37612700
C	2.83949000	5.96399100	-0.26113500
C	4.08403300	5.56728100	0.52496600
C	1.13634400	7.31721600	1.61186100
O	1.96326600	-1.96492200	-1.72934400
C	3.10026400	-1.70766800	-1.04898400
C	3.58117700	-2.92683600	-0.30584500
C	5.08440900	-2.85851600	-0.00865000
C	2.70777500	-3.01649500	0.96395500
O	3.59754100	-0.61203300	-1.01773200
C	2.99869100	-4.23575200	1.83125900
C	5.78527600	-4.20836400	-0.15163400
C	7.25789400	-4.12677600	0.23281800
C	2.04353300	-4.31443700	3.01764600
C	-2.11159000	-2.59481100	-0.81877600
C	-3.32660700	-1.81713000	-0.61170600
S	-3.71573700	-0.46750500	-1.60385300
N	-4.25416400	-2.08429200	0.28117800
C	-5.33623500	-1.20127000	0.26824900
C	-5.20150400	-0.22585500	-0.71774600
C	-6.44232400	-1.22961300	1.11299300
C	-6.17329200	0.75314000	-0.89720900
C	-7.40375400	-0.25371700	0.93436700
C	-7.27198400	0.72570300	-0.05866300
C	-4.12554000	-3.11644000	1.31112900
H	2.54735300	1.57859000	-2.23947300
H	2.98516300	3.83574100	-1.62218000
H	2.96382100	5.76167900	-1.32286900
H	2.67219500	7.03430600	-0.18772800
H	4.26811700	4.49466900	0.44691600
H	4.95843300	6.09633600	0.14491200
H	3.96361300	5.81629100	1.58078700
H	-0.14408900	5.66614900	1.21852600
H	1.20817000	5.27249000	2.27474300
H	0.50569200	7.61865500	2.44779800
H	2.16747100	7.55498600	1.87213000
H	0.84577300	7.90639600	0.74174300
H	1.00847200	-4.38979800	2.67775400
H	2.25760300	-5.18160700	3.64300300
H	2.12625500	-3.42105600	3.64001700
H	7.36840600	-3.82751400	1.27731400
H	7.75811600	-5.08705100	0.10320500
H	7.77856700	-3.38920800	-0.38167500
H	5.28439700	-4.95945000	0.46329300
H	2.91364100	-5.14113700	1.22316600
H	5.55997800	-2.14524900	-0.68545900
H	5.23691900	-2.46638000	1.00206900
H	2.86139100	-2.10685600	1.55506300
H	1.65346900	-3.03277000	0.67148700
H	4.02863800	-4.19359000	2.19428400
H	5.69037500	-4.54483100	-1.18809200
H	3.36114000	-3.79636300	-0.93226600
H	0.56219900	-1.24833700	-2.98628800

H	1.97993800	-0.20663300	-2.81787700
H	-0.17959700	3.61433400	1.33799400
H	-0.07841300	-2.75021300	-1.11939100
H	-2.25486900	-3.65185900	-1.01011700
H	-6.54603500	-1.98507200	1.87962400
H	-6.06801600	1.50854700	-1.66356300
H	-8.04340000	1.47531000	-0.17198700
H	-8.27507100	-0.24706000	1.57491600
H	-4.95564300	-3.81362500	1.22289800
H	-3.18265800	-3.63479300	1.17747600
H	-4.13718400	-2.62988100	2.28464000

Z-form TS_1 state

C	3.01123000	2.56502200	-0.36898200
C	1.83285700	2.20255900	0.33939600
C	0.96413800	1.27439000	-0.20511000
O	-0.16377900	0.96629700	0.53986400
C	-1.12997200	0.05479600	0.16862300
C	-0.92837400	-0.63880500	-1.10901700
C	0.20027600	-0.33523500	-1.88631500
C	1.17217600	0.63226800	-1.44731600
C	2.35286100	1.00028300	-2.14792500
C	3.23898000	1.92206500	-1.63682700
O	-2.07065800	-0.12384500	0.94927800
C	-1.79311900	-1.77605300	-1.42833800
C	0.40011700	-1.04604400	-3.12268400
N	3.88727900	3.47700400	0.14031400
C	3.62045700	4.03354100	1.48413400
C	5.15230300	3.76840600	-0.55140900
C	6.23082900	2.71505900	-0.27821400
C	4.62595400	5.07179300	1.96953100
O	0.90439500	-3.13079800	-1.92878900
C	2.04559300	-2.75466800	-1.40394400
C	2.27081100	-3.24514800	0.03483100
C	3.73579100	-3.09515200	0.47560700
C	1.27609800	-2.41138700	0.88074600
O	2.84646100	-1.95531000	-1.94696000
C	1.33337700	-2.62232400	2.39745100
C	4.19932600	-4.18210400	1.45687500
C	5.63934300	-3.95719500	1.93049700
C	0.27298900	-1.77491500	3.11181000
C	-3.11704700	-1.90327500	-1.17347800
C	-3.97811000	-0.82370100	-0.71866200
S	-4.01864700	0.74428400	-1.53689600
N	-4.81856700	-0.89711900	0.30136400
C	-5.54782500	0.28464000	0.56006200
C	-5.23569300	1.31206500	-0.34127200
C	-6.47895800	0.47942200	1.58851000
C	-5.84418100	2.56318800	-0.24974300
C	-7.08727500	1.73155900	1.68251700
C	-6.77457200	2.76315600	0.77385900
C	-4.89831800	-2.04560800	1.21837000
H	2.58883200	0.53328300	-3.10321400
H	4.13425500	2.14444500	-2.21267600
H	4.95486200	3.86003800	-1.63041600
H	5.49911700	4.75851900	-0.23079500
H	5.89765500	1.71226000	-0.59789900
H	7.15638200	2.96309500	-0.82428800
H	6.46525600	2.66987000	0.79922900
H	2.61617300	4.49282200	1.47153400
H	3.58078300	3.19761000	2.20821900
H	4.32976400	5.38911000	2.98258600
H	5.64919100	4.66665800	2.03263900
H	4.63579100	5.96872300	1.32893400
H	-0.74163600	-2.03494100	2.75979900
H	0.30591900	-1.92165600	4.20485000
H	0.42960000	-0.69991300	2.90586000
H	5.72904500	-2.99680000	2.46975100
H	5.97311000	-4.76038100	2.60952800
H	6.33381600	-3.92638600	1.07151400
H	3.52329600	-4.22344000	2.32935300
H	1.17996400	-3.69376000	2.62556900
H	4.38713100	-3.12110200	-0.41566100
H	3.88095200	-2.09219200	0.92524700
H	1.46619700	-1.33591900	0.66837000
H	0.25073400	-2.63463500	0.52910300
H	2.33665300	-2.35557800	2.77626500
H	4.11879600	-5.16631600	0.95753400
H	1.96769900	-4.30649300	0.08378500

H	-0.43036600	-1.56719200	-3.60562300
H	1.33175600	-0.94155200	-3.68258900
H	1.57537600	2.62141000	1.30801500
H	-1.26747400	-2.64550800	-1.84175100
H	-3.60029900	-2.86886200	-1.35545500
H	-6.72317200	-0.31720400	2.29155300
H	-5.60248200	3.36256500	-0.95184700
H	-7.26393600	3.73333400	0.86916300
H	-7.81646100	1.91229900	2.47320200
H	-5.92581300	-2.43770400	1.22672600
H	-4.20253000	-2.82628900	0.88854600
H	-4.61029300	-1.70631900	2.22529800

Z-form TS_2 state

C	-2.91240200	1.48626900	1.35854500
C	-1.72301300	1.56454000	0.58551500
C	-0.73984200	0.60227900	0.73694600
O	0.38144500	0.73402300	-0.06235500
C	1.42068900	-0.17963200	-0.10319600
C	1.33086600	-1.31833800	0.80705400
C	0.21870200	-1.46547900	1.62684600
C	-0.84176400	-0.50197100	1.61547600
C	-2.03087000	-0.55782000	2.39613000
C	-3.02213700	0.39266900	2.28384500
O	2.32422100	0.04551000	-0.91821100
C	2.37256900	-2.35262400	0.75637900
C	0.12699400	-2.69088800	2.51512500
N	-3.91149900	2.40447300	1.20739700
C	-3.78687700	3.40453300	0.12690100
C	-5.13703200	2.31249700	2.01400800
C	-6.15163600	1.31026700	1.45531900
C	-4.96774200	4.35924200	-0.01228000
O	-1.16721500	-3.17772900	-0.56700300
C	-2.40680300	-2.66017900	-0.44310500
C	-2.86155100	-1.86621000	-1.65687500
C	-4.07088600	-0.97394500	-1.31562700
C	-1.65645300	-1.09639100	-2.24343400
O	-3.08345900	-2.84096700	0.58018200
C	-1.96455700	-0.11954100	-3.38216700
C	-5.08860600	-0.84904300	-2.45951700
C	-6.22126200	0.12709900	-2.12790600
C	-0.68657900	0.57758200	-3.86654800
C	3.69005500	-2.20205200	0.48333600
C	4.37392600	-0.92498800	0.36548100
S	4.28089600	0.30145200	1.63913300
N	5.14717700	-0.55962000	-0.64524700
C	5.70127000	0.73546200	-0.53766800
C	5.32778400	1.37833800	0.65044800
C	6.52229400	1.36972800	-1.47874000
C	5.76462000	2.67005400	0.94052800
C	6.95886000	2.66357600	-1.19075000
C	6.58553000	3.30795800	0.00603000
C	5.30603800	-1.34588100	-1.87828000
H	-2.19262600	-1.38493400	3.08668400
H	-3.91521800	0.27554500	2.89332900
H	-4.85743100	2.05287900	3.04707600
H	-5.58652300	3.31209900	2.07276800
H	-5.69860700	0.31012600	1.33422000
H	-7.01771600	1.22066900	2.13286700
H	-6.51730900	1.63642600	0.46729300
H	-2.86934700	3.99620000	0.29816800
H	-3.64264400	2.86389000	-0.82885600
H	-4.78461400	5.01017400	-0.88263400
H	-5.91611700	3.82488600	-0.18727800
H	-5.08233200	5.00935500	0.87023500
H	0.04201700	-0.16015500	-4.24835300
H	-0.89821400	1.29863100	-4.67434700
H	-0.20444100	1.12351800	-3.03493000
H	-5.82100700	1.14040600	-1.93641100
H	-6.94839300	0.20000600	-2.95476600
H	-6.76537000	-0.19581400	-1.22225700
H	-4.58431900	-0.52785700	-3.38735600
H	-2.44226300	-0.65767900	-4.22251800
H	-4.58488500	-1.38750100	-0.43013400
H	-3.70679200	0.03289000	-1.02086300
H	-1.18072700	-0.52780300	-1.42405600
H	-0.90499400	-1.82818200	-2.58565200
H	-2.68861700	0.64327700	-3.03995100
H	-5.50781300	-1.85190100	-2.66791700

H	-3.18428700	-2.62303300	-2.40296500
H	1.07676500	-2.83550100	3.05404100
H	-0.67820800	-2.59835700	3.25355600
H	-1.54215900	2.35536300	-0.13756900
H	2.02069800	-3.37677700	0.91630500
H	4.31581600	-3.09537600	0.38417200
H	6.81126300	0.87261300	-2.40501900
H	5.47446300	3.16975700	1.86596500
H	6.93953300	4.31981300	0.20777100
H	7.59889000	3.18219200	-1.90561300
H	6.37152900	-1.56624800	-2.03915500
H	4.73997200	-2.28077400	-1.78835900
H	4.90575100	-0.75952000	-2.71965400
O	-0.12026500	-3.83721400	1.65350500
H	-0.17847800	-4.68719200	2.14208000
H	-0.79825600	-3.57496000	0.30305900

5d(PP) ground state

C	-5.11645400	0.09039500	-0.29776000
C	-4.16898100	-0.90819500	0.00849500
C	-2.82404800	-0.62038200	-0.03222300
O	-1.99129800	-1.64771700	0.28089800
C	-0.63112000	-1.53537900	0.30639000
C	-0.05833500	-0.24307900	-0.05517500
C	-0.89376800	0.81547000	-0.36809900
C	-2.30411900	0.64662800	-0.35389700
C	-3.26931200	1.64604800	-0.62932300
C	-4.60932300	1.39308400	-0.59743300
O	-0.01864600	-2.52691700	0.62199100
C	1.37513300	-0.08051100	-0.07024400
C	-0.29081900	2.15944300	-0.71264100
N	-6.44500100	-0.17720700	-0.30607700
C	-6.89116100	-1.49613300	0.16879600
C	-7.41546900	0.91347500	-0.40007000
C	-7.60299200	1.64423400	0.92447700
C	-8.38110000	-1.75384300	0.03335900
C	2.32454500	-0.99334100	0.23124700
C	3.72820800	-0.69332200	0.14558700
S	4.83698200	-1.92617200	-0.33875700
N	4.33335300	0.46230800	0.39949500
C	5.71384400	0.44929700	0.16808500
C	6.16980900	-0.80507400	-0.23177600
C	6.59185200	1.52167700	0.30259800
C	7.51631100	-1.03514100	-0.48968100
C	7.92927700	1.29214000	0.03901600
C	8.38863900	0.02869400	-0.35034800
C	3.68868300	1.64589600	0.97917500
O	0.42293400	2.62571600	0.42599300
H	-2.95686200	2.65321900	-0.86206800
H	-5.28401600	2.20906600	-0.80091300
H	-7.10148800	1.59678000	-1.18637000
H	-8.35883500	0.49551900	-0.73740300
H	-6.66200300	2.07668300	1.26766700
H	-8.33248600	2.44702200	0.81414700
H	-7.96153100	0.95658000	1.69220100
H	-6.36188600	-2.25344600	-0.41158900
H	-6.59042400	-1.62063700	1.21544500
H	-8.57493200	-2.77368800	0.36450300
H	-8.97894500	-1.08504800	0.65204700
H	-8.71100100	-1.66894000	-1.00213300
H	0.37551400	2.03962800	-1.57292200
H	-1.06046500	2.86984900	-0.99804500
H	-4.45153300	-1.91119100	0.28436400
H	1.70901100	0.89893600	-0.38006000
H	2.07075600	-2.01539400	0.46809100
H	6.24415900	2.50390900	0.58968200
H	7.86398300	-2.01237800	-0.79500600
H	9.44172200	-0.11865000	-0.54797800
H	8.63224000	2.10873700	0.13187700
H	4.37603000	2.08083500	1.69926500
H	3.45750300	2.36890300	0.19847700
H	2.77786500	1.34525500	1.48635400
H	0.70774500	3.53036200	0.25417300

Valproic acid ground state

O	-2.73431200	-0.62892500	0.83508100
C	-1.76424100	-0.93674800	-0.04199400
C	-0.45768100	-0.26082600	0.29317300
C	0.73799400	-0.98903100	-0.33460000
C	-0.58713900	1.20800800	-0.16272900

O	-1.97086300	-1.64187000	-0.99810500
C	0.63908000	2.05968900	0.14678900
C	1.95568000	-1.04889500	0.58619700
C	3.15138700	-1.70396900	-0.09526100
C	0.42354300	3.51572500	-0.25258000
H	-0.42037000	3.94633300	0.29000600
H	1.30558300	4.12042100	-0.03952700
H	0.21005700	3.59601000	-1.32048700
H	3.45441100	-1.13356600	-0.97598000
H	4.00857800	-1.76695400	0.57593700
H	2.90435800	-2.71600800	-0.42313600
H	2.22621000	-0.04456400	0.91961000
H	0.86337000	1.99602400	1.21568800
H	0.44946900	-2.00912000	-0.59657200
H	1.00896700	-0.49930800	-1.27563400
H	-0.77115200	1.22070400	-1.24264200
H	-1.46200700	1.65403700	0.31785200
H	1.50882100	1.66277000	-0.38265600
H	1.68786800	-1.61078200	1.48571300
H	-0.36595000	-0.26853800	1.38361300
H	-3.55855000	-1.04482300	0.53843000
